# ATR-dependent phosphorylation of the histone acetyltransferase HBO1 suppresses chromatin binding and promotes replication stress responses

**DOI:** 10.1016/j.jbc.2026.113419

**Published:** 2026-08-10

**Authors:** Chunyan Zong, Jiamin Zhang, Zhe Zhang, Yujuan Cai, Yiran Wang, Yan Fang, Guopei Zheng, Qian Li, Lulu Wang, Jianfeng Shen

**Affiliations:** 1 Department of Ophthalmology, Ninth People’s Hospital, Shanghai Jiao Tong University School of Medicine, Shanghai, China; 2 Shanghai Key Laboratory of Orbital Diseases and Ocular Oncology, Shanghai, China; 3 Institute of Translational Medicine, National Facility for Translational Medicine, Shanghai Jiao Tong University, Shanghai, China; 4 Department of Ophthalmology, Shanghai General Hospital, Shanghai Jiao Tong University School of Medicine, Shanghai, China; 5 Department of Chemical Biology, School of Pharmacy, Tianjin Medical University, Tianjin, China

**Keywords:** HBO1, phosphorylation, histone acetyltransferase, replication stress, ATR

## Abstract

Mounting evidence has shown that histone acetyltransferase binding to ORC1 (HBO1) serves as an oncoprotein, warranting the use of the small molecule inhibitor WM-3835 for cancer therapy. However, HBO1 is ubiquitously expressed in both tumor and normal tissues, with potential to increase the risk of systemic toxicity. This unmet need highlights the importance of identifying suitable biomarkers to predict the sensitivity to HBO1 inhibitor. Here, we show that ATR, a key regulator of DNA replication stress, is a novel interacting partner of HBO1. In addition, we reveal a regulatory function of HBO1 in DNA replication stress responses, in an ATR-dependent manner. Mechanistically, ATR mediated HBO1 Ser50/53 phosphorylation interferes with the genomic binding of HBO1 and regulates gene expression. Notably, overexpression of HBO1 mutated at the ATR phosphorylation site (S50/53A) dampens the expression of DNA repair related genes and suppresses tumor colony formation, consistent with the observations of WM-3835 treatment. Inhibition of ATR significantly antagonized the sensitivity to WM-3835 treatment. Collectively, our findings uncovered a previously unidentified role of HBO1 in the regulation of replication stress and discovered ATR as a potential biomarker for WM-3835 treatment.

The histone acetyltransferase binding to ORC1 (HBO1, also termed KAT7 or MYST2) is a key member of the MYST family of acetyltransferases. Functioning as the core catalytic subunit of the HBO1 complex, which incorporates various cofactors and co-proteins, HBO1 specifically acetylates histones H3 at lysine 14 (H3K14ac) and H4 at lysine 5, 8, and 12 (H4K5, H4K8, H4K12ac) (1, 2, 3, 4). By modulating histone acetylation, HBO1 induces chromatin relaxation, facilitating the recruitment of transcription factors and thereby regulating transcriptional initiation (5, 6, 7). Consequently, HBO1 plays pivotal roles in diverse biological processes, including chromatin remodeling (4), DNA replication (8, 9), transcriptional regulation (10), protein ubiquitination (11), immune response modulation (12), stem cell pluripotency, self-renewal maintenance (13), and embryonic development (1).

Emerging evidence highlights the oncogenic role of HBO1 in multiple malignancies, where its overexpression promotes cancer cell proliferation, cell cycle progression, and drug resistance (14, 15, 16, 17). For instance, HBO1-mediated H3K14ac enhances the processivity of RNA polymerase II, sustaining the expression of oncogenes (*e.g.*, *HOXA9* and *HOXA10*) in leukemia stem cells (18). In addition, HBO1 facilitates gastric cancer progression by activating YAP1 (19) and drives bladder cancer proliferation through the Wnt/β-catenin pathway (20). Despite these findings, the precise mechanisms underlying the role of HBO1 in tumorigenesis remain incompletely understood, warranting further investigation.

Given its oncogenic properties, targeting HBO1 has emerged as a promising therapeutic strategy. Genetic knockdown or pharmacological inhibition of HBO1 effectively suppresses tumor growth (21, 22). In 2019, MacPherson *et al.* developed WM-3835, a potent small-molecule inhibitor that competitively binds the acetyl-CoA site of HBO1, selectively depleting H3K14ac levels (18). Preclinical studies demonstrate that WM-3835 exhibits robust antitumor activity in non-small cell lung cancer (23), prostate cancer (24), osteosarcoma (25), and acute lymphoblastic leukemia (26), inducing G1/S cell cycle arrest and apoptosis. However, since HBO1 is ubiquitously expressed in both normal and malignant cells, the potential toxicity of WM-3835 necessitates the identification of predictive biomarkers and patient stratification strategies to optimize its clinical translation.

In this study, we employed immunoprecipitation-mass spectrometry (IP-MS) to identify HBO1-interacting proteins, revealing ATR, a critical kinase involved in DNA damage response (DDR) and replication stress, as a novel binding partner. Mechanistically, we demonstrate that ATR phosphorylates HBO1, modulating its function in gene expression regulation and replication stress management. Our findings elucidate an ATR-HBO1 signaling axis, providing new insights into HBO1 function in cancer progression, and highlighting ATR as a potential predictive biomarker for HBO1 inhibitor therapy.

## Results

### Proteomic analysis indicates that ATR is a key HBO1 interacting protein

**Figure 1:**
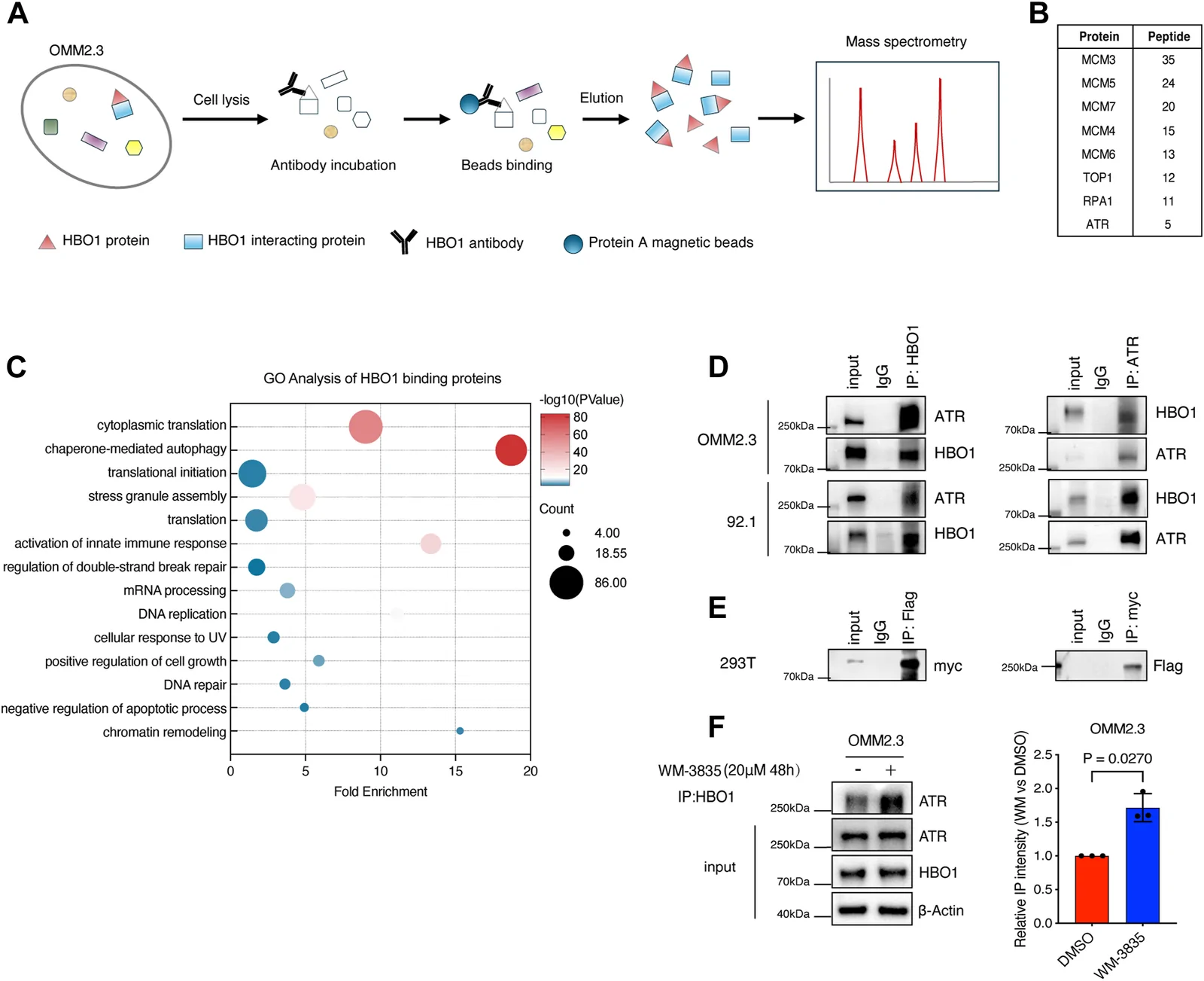
**IP-MS identifies ATR as a HBO1 interacting protein.***A*, schematic representation of the IP-MS experimental workflow. *B*, representative list of proteins identified through HBO1 IP-MS analysis. *C*, GO enrichment analysis of proteins co-immunoprecipitated with HBO1. *D*, co-IP analysis of endogenous HBO1 and ATR interaction. Proteins were detected by western blotting, with 1% cell lysate loaded as input control. Molecular weight markers (kDa) are indicated. *E*, co-IP of exogenously expressed HBO1 and ATR. Myc-tagged HBO1 and Flag-tagged ATR were detected by western blotting. *F*, OMM2.3 cells were treated with DMSO (vehicle control) or WM-3835 (20 μM) for 48 h, followed by co-IP assays using anti-HBO1 antibody. *Left*: representative Western blot images showing endogenous ATR pulled down by HBO1, with ATR expression in input samples examined. *Right*: bar plot of relative IP ATR intensity (WM-3835 normalized to DMSO, DMSO = 1). Statistical comparison of normalized IP signals was performed *via* paired *t* test. Data are presented as mean ± SD. ATR, Ataxia Telangiectasia and Rad3-related; co-IP, co-immunoprecipitation; DMSO, dimethyl sulfoxide; HBO1, histone acetyltransferase binding to ORC1; IP, immunoprecipitation; IP-MS, immunoprecipitation-mass spectrometry.

We here used melanoma as a model to elucidate the biological function of HBO1. To investigate the function of HBO1 in tumor pathogenesis, we first identified the interacting proteins involved. We immunoprecipitated endogenous HBO1 binding proteins from the uveal melanoma (UM) cell line OMM2.3 using anti-HBO1 antibodies, and analyzed the coprecipitated proteins by mass spectrometry (Fig. 1*A*, Table S1). Among 993 identified binding partners (iBAQ intensity > 0 in IP and input), we detected known HBO1-associated proteins (*e.g.*, MCMs) and novel interactors (Fig. 1*B*). Gene Ontology (GO) analysis revealed enrichment in DNA damage repair, replication, and translation pathways (Fig. 1*C*). Strikingly, ATR, a central kinase in replication stress response, emerged as an HBO1 interactor.

We validated this interaction endogenously in OMM2.3 and 92.1 UM cells *via* reciprocal co-immunoprecipitation (Co-IP) with HBO1- and ATR-specific antibodies (Fig. 1*D*) and exogenously in 293T cells overexpressing Flag-ATR and myc-HBO1 (Fig. 1*E*). Intriguingly, pharmacological inhibition of the acetyltransferase activity of HBO1 enhanced its binding to ATR (Fig. 1*F*), suggesting a regulatory crosstalk between HBO1 acetylation and ATR interaction.

### ATR interacts with HBO1 and phosphorylates HBO1 at Ser50 and Ser53

**Figure 2:**
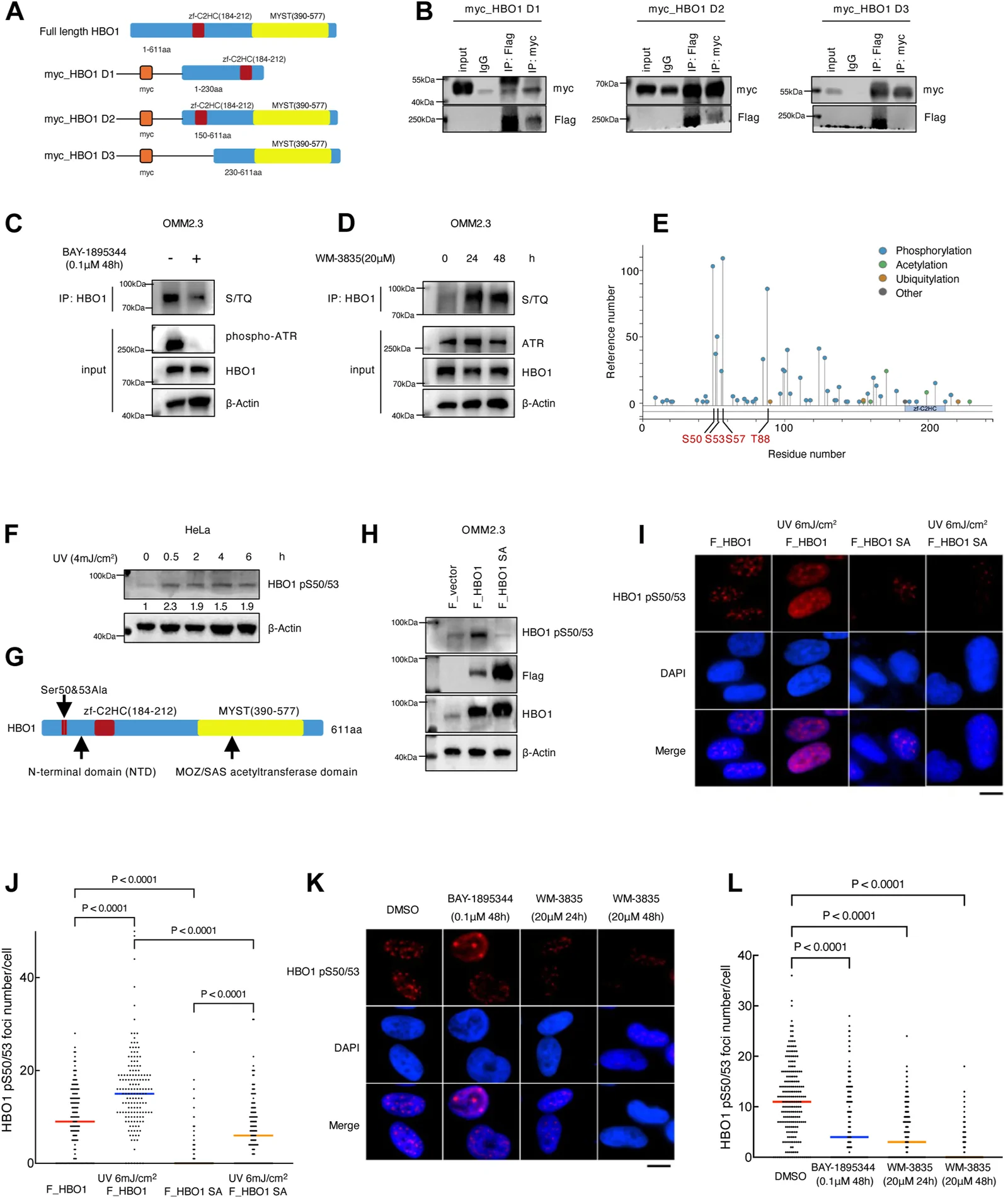
**ATR interacts with HBO1 and phosphorylates HBO1 at Ser50/53.***A*, schematic of myc-tagged HBO1 domain constructs. *B*, co-IP analysis of myc-tagged HBO1 domains or Flag-tagged ATR in 293T cells. *C*, HBO1 immunoprecipitation from OMM2.3 cells pretreated with DMSO or BAY-1895344 (ATR inhibitor). ATR phosphorylation (S/TQ motif) was detected by immunoblot after IP. *D*, time-course analysis of HBO1 immunoprecipitation from OMM2.3 cells treated with WM-3835 for 0, 24, or 48 h. *E*, *in silico* prediction of HBO1 PTM sites. *F*, validation of HBO1 pS50/53 antibody specificity in HeLa cells post-UV (4 mJ/cm^2^). Lysates were collected at indicated timepoints (0–6 h). *G*, schematic of HBO1 Ser50/Ser53-to-Ala (SA) mutant construct. *H*, immunoblot confirmation of Flag-tagged wild-type HBO1 (F_HBO1) and SA mutant overexpression in OMM2.3 cells. *I*, IF of HBO1 pS50/53 staining in OMM2.3 cells expressing F_HBO1 or F_HBO1 SA, with or without UV irradiation (6 mJ/cm^2^). Cells were fixed 1 h post-irradiation. The scale bar represents 10 μm. *J*, scatter plot quantifying the number of HBO1 pS50/53 foci per OMM2.3 cell under indicated conditions in (*I*). Each *dot* represents an individual cell; *horizontal lines* denote the median value (n = 147, 157, 154, and 141 cells per group). Statistical significance was determined by the Kruskal–Wallis test followed by Dunn’s multiple comparisons test. *K*, IF of HBO1 pS50/53 staining in OMM2.3 cells treated with DMSO, BAY-1895344, or WM-3835. *L*, scatter plot quantifying the number of HBO1 pS50/53 foci per OMM2.3 cell under indicated conditions in (*K*). Each *dot* represents an individual cell; *horizontal lines* denote the median value (n = 215, 145, 216, and 132 cells per group). Statistical significance was determined by the Kruskal–Wallis test followed by Dunn’s multiple comparisons test. ATR, Ataxia Telangiectasia and Rad3-related; DMSO, dimethyl sulfoxide; HBO1, histone acetyltransferase binding to ORC1; PTM, posttranslational modification.

ATR is a critical regulator of replication stress response; however, the molecular mechanism underlying its interaction with HBO1 remains poorly understood. Structurally, HBO1 consists of an N-terminal domain (NTD) harboring a zinc finger motif and a C-terminal MYST catalytic domain (27). To map the ATR interaction domain, we generated lentiviral expression vectors encoding myc-tagged HBO1 truncation mutants: D1 (1–230aa), D2 (150–611aa) and D3 (230–611aa) (Fig. 2*A*). Intriguingly, co-IP assays revealed that the N-terminal zinc finger-containing region (encompassing D1 and D2) mediates the interaction with ATR (Fig. 2*B*). Previous studies have established that UV-induced replication stress triggers ATR-dependent phosphorylation of HBO1, facilitating DNA damage repair (28, 29). This prompted us to investigate whether HBO1 undergoes ATR-dependent phosphorylation in OMM2.3 cells. We immunoprecipitated endogenous HBO1 from cells treated with vehicle dimethyl sulfoxide or a selective ATR inhibitor, and immunoblotted precipitates using an anti-phospho-S/TQ antibody that recognizes canonical ATR phosphorylation motifs. We detected robust phospho-S/TQ signals on HBO1, and this phosphorylation signal was drastically reduced upon chemical inhibition of ATR activity (Fig. 2*C*). Moreover, pharmacological suppression of HBO1 acetyltransferase activity elevated S/TQ phosphorylation levels (Fig. 2*D*), corroborating our findings in Figure 1*F*. These results collectively demonstrate that HBO1 is likely targeted by ATR signaling for phosphorylation and warrant further investigation of its phosphorylation sites.

Previous studies have identified critical serine (Ser) and threonine (Thr) phosphorylation sites within the NTD of HBO1 (30, 31). PhosphoSitePlus database annotation highlighted Ser50, Ser53, Ser57, and Thr88 as the most probable phosphorylation sites on HBO1 (Fig. 2*E*). While Ser57 is a known PLK1 target (31) and Thr88 is phosphorylated by CDK1 (32), we hypothesized that ATR preferentially targets Ser50/Ser53. To test this, we generated a polyclonal antibody against phospho-Ser50/53 (HBO1 pS50/53) and validated its specificity under UV irradiation. HBO1 pS50/53 signals increased within 0.5 h post-UV exposure (Fig. 2*F*), confirming antibody efficacy. We subsequently introduced alanine substitutions at Ser50/Ser53 (HBO1 SA) to abrogate phosphorylation and constructed Flag-tagged WT (F_HBO1) and mutant (F_HBO1 SA) expression plasmids (Fig. 2*G*). Western blot analysis of transfected OMM2.3 cells confirmed equivalent expression of both constructs, while HBO1 pS50/53 signals were selectively abolished in the mutant (Fig. 2*H*). Immunofluorescence (IF) further validated the antibody specificity and the phospho-deficient phenotype of HBO1 SA-expressing cells (Figs. 2, *I*–*L* and S1, *A*–*D*). Interestingly, IF revealed that HBO1 acetyltransferase inhibition dramatically reduced HBO1 pS50/53 foci formation-a contrast to Western blot data-suggesting that the S/TQ motif detected in Figure 2*D* encompasses additional phosphorylation sites beyond Ser50/53.

### ATR-mediated HBO1 Ser50/53 phosphorylation regulates gene expression

**Figure 3:**
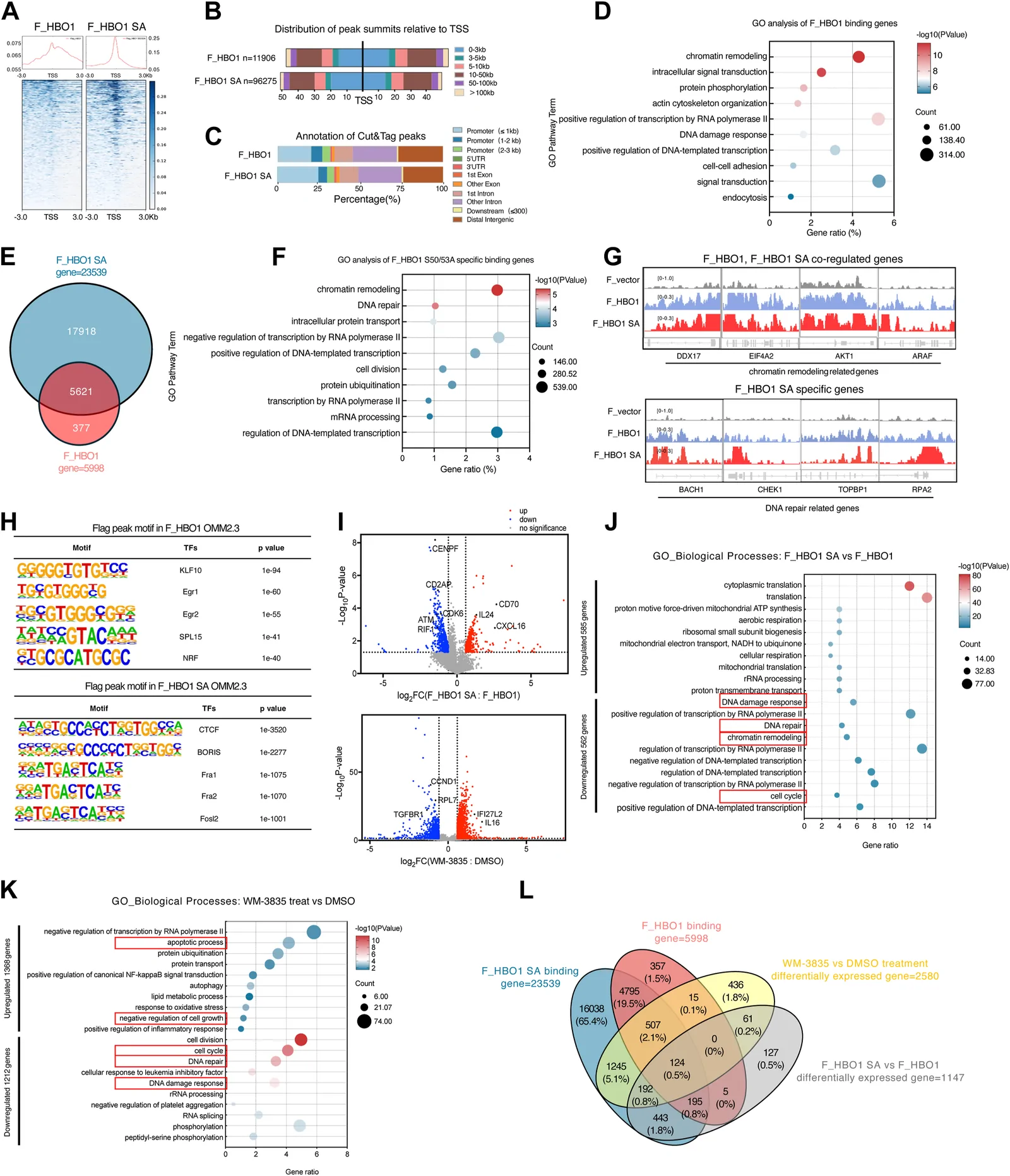
**HBO1 regulates gene expression through Ser50/53 in an ATR dependent manner.***A*, CUT&Tag signal densities (*top*) and heatmap (*bottom*) of Flag-tagged HBO1 and HBO1 SA binding at TSS (±3 kb). *B*, distribution profile of CUT&Tag peaks relative to TSS positions. Color-coding indicates genomic regions as shown in the labels on the *right*. *C*, genomic annotation of Flag-tagged peaks identified by CUT&Tag. *D*, GO analysis of biological processes associated with F_HBO1-bound genes from CUT&Tag data. *E*, Venn diagram comparing F_HBO1 (*red*) and F_HBO1 SA (*blue*) binding genes. *F*, GO analysis of biological processes specifically associated with F_HBO1 SA-bound genes. *G*, genome browser tracks showing: (*top*) coregulated genes by F_HBO1 and F_HBO1 SA, enriched in chromatin remodeling; (*bottom*) F_HBO1 SA-specific target genes involved in DNA repair. *H*, motif enrichment analysis of peak sequences from F_HBO1 and F_HBO1 SA expressed cells. *I*, scatter plots of DEGs: (*top*) F_HBO1 SA *versus* F_HBO1; (*bottom*) WM-3835 (20 μM) *versus* DMSO-treated cells. DEG thresholds: |log2FC| ≥ 0.585, *p* < 0.05. *J*, GO analysis of biological processes for DEGs (F_HBO1 SA *versus* F_HBO1; |log2FC| ≥ 0.585, *p* < 0.0s5). *K*, GO analysis of biological processes for DEGs after 48-h WM-3835 (20 μM) treatment (*versus* DMSO; |log2FC| ≥ 0.585, *p* < 0.05). *L*, overlap between CUT&Tag-identified binding genes and RNA-seq DEGs from (*I*). ATR, Ataxia Telangiectasia and Rad3-related; CUT&Tag, Cleavage Under Targets and Tagmentation; DEG, differentially expressed gene; GO, Gene Ontology; HBO1, histone acetyltransferase binding to ORC1; TSS, transcription start site; UTR: untranslated region.

To elucidate the biological significance of HBO1 phosphorylation at Ser50/53, we investigated how these modifications influence the genomic occupancy of HBO1, given its established role in transcriptional regulation *via* histone acetylation. Using Cleavage Under Targets and Tagmentation (CUT&Tag)-seq with an anti-Flag antibody, we profiled genome-wide binding patterns in OMM2.3 cells expressing Flag-tagged empty vector (control), WT HBO1 (F_HBO1), or the phospho-deficient mutant (F_HBO1 SA, Ser50/53Ala) (Fig. S2*A*). Strikingly, the F_HBO1 SA mutant exhibited markedly enhanced chromatin binding compared to WT HBO1, as evidenced by increased sequencing read intensities and larger DNA fragment sizes (Fig. S2, *B* and *C*). While WT HBO1 occupied 5998 genes, the SA mutant bound to 23,539 genes—a ∼4-fold increase (Fig. S2*D*). These findings demonstrate that ablation of Ser50/53 phosphorylation substantially augments the chromatin affinity of HBO1. Peak distribution analysis revealed that both F_HBO1 and F_HBO1 SA were predominantly localized to transcription start sites (TSSs), consistent with HBO1’s role in transcriptional regulation (Fig. 3, *A* and *B*). Quantitatively, 31.78% of F_HBO1 peaks and 34.29% of F_HBO1 SA peaks mapped to promoter regions (Fig. 3*C*), further supporting HBO1’s functional association with gene regulatory (1).

GO analysis demonstrated that genes bound by HBO1 were significantly enriched in chromatin remodeling-related biological processes (Fig. 3*D*). Comparative genomic localization revealed substantial overlap between WT HBO1 and HBO1 SA binding genes, with 93.7% of HBO1-bound genes also occupied by the SA mutant (Fig. 3*E*). Strikingly, the HBO1 SA mutant uniquely bound to 17,918 additional genes, predominantly involved in chromatin remodeling and DNA repair pathways (Fig. 3*F*), indicating a close correlative association between HBO1 phosphorylation deficiency and expanded genomic binding capacity. Both WT and mutant HBO1 consistently occupied key chromatin regulatory genes including *DDX17*, *EIF4A2*, *AKT1*, and *ARAF* (Fig. 3*G*). However, the SA mutant specifically enriched at DNA repair genes (*e.g.*, *BACH1*, *CHK1*, *TOPBP1*, *RPA2*), indicating a phosphorylation-dependent shift in target preference (Fig. 3*G*). Motif analysis uncovered differential binding patterns: while WT HBO1 preferentially associated with KLF10 motifs (consistent with prior reports (33)), the SA mutant showed strong affinity for CTCF binding sites (Fig. 3*H*). Given the established role of CTCF in chromatin architecture maintenance (34), we propose that phospho-deficient HBO1 may modulate chromatin three-dimensional organization, potentially facilitating its expanded genomic occupancy.

To elucidate the functional consequences of HBO1 Ser50/53 phosphorylation on transcriptional regulation, we performed RNA sequencing analysis in OMM2.3 cells overexpressing either WT F_HBO1 or the phospho-deficient F_HBO1 SA mutant. Comparative transcriptome profiling identified 585 significantly upregulated and 562 downregulated genes in F_HBO1 SA-expressing cells compared to WT controls (|log_2_FC| ≥ 0.585, *p* value<0.05; Fig. 3*I*). GO analysis revealed that the downregulated gene set was remarkably enriched for biological processes related to DNA damage repair and chromatin remodeling (Fig. 3*J*). Intriguingly, this transcriptional repression pattern correlated with the expanded chromatin binding capacity of the SA mutant observed in our CUT&Tag analysis (Fig. 3*F*). These findings support a correlative link that the phospho-deficient HBO1 SA mutant may function as a transcriptional repressor of DNA repair genes through its enhanced chromatin occupancy at these loci.

To investigate the functional significance of HBO1 Ser50/53 phosphorylation in WM-3835-mediated H3K14ac inhibition and tumor suppression, we first conducted transcriptome profiling of control and WM-3835-treated OMM2.3 cells (Fig. 3, *I* and *K*). RNA-seq analysis revealed significant transcriptional changes, with 1368 genes upregulated and 1212 genes downregulated following WM-3835 treatment (Fig. 3*K*). Notably, upregulated genes were enriched in apoptosis and growth inhibition pathways, consistent with the tumoricidal effect of WM-3835, while downregulated genes were associated with DNA damage repair and cell cycle progression, mirroring the transcriptional profile observed with Ser50/53 mutation. Comparative analysis of differentially expressed genes (DEGs) identified 1147 and 2580 genes altered by HBO1 SA mutation and WM-3835 treatment, respectively, with 377 coregulated genes exhibiting largely concordant expression patterns (Fig. S2, *E* and *F*).

To determine whether these transcriptional changes were directly mediated by HBO1 binding, we integrated our CUT&Tag and RNA-seq datasets and stratified target genes according to promoter occupancy (Figs. 3*L* and S3, *A*–*F*). Among 5621 genes bound by both WT and mutant HBO1, 631 showed WM-3835-responsive expression changes, while 319 were affected by Ser50/53 mutation (Fig. S3, *A* and *B*). The phospho-deficient mutant uniquely bound a large number of additional genes, predominantly at promoter regions, indicating that HBO1 SA-mediated transcriptional regulation occurs primarily through direct promoter binding (Fig. S3*E*).

Strikingly, genes specifically bound by HBO1 SA exhibited more pronounced transcriptional alterations following WM-3835 treatment, with upregulation of apoptosis-related genes and suppression of cell division, migration, and DNA repair pathways (Fig. S3*C*). Importantly, HBO1 SA preferentially bound and repressed genes involved in DDR and cell cycle regulation (Fig. S3*D*). Collectively, these findings demonstrate that ATR-mediated phosphorylation of HBO1 at Ser50/53 modulates H3K14ac dynamics and gene expression programs. The convergence of WM-3835-induced transcriptional changes with HBO1 SA-mediated regulation suggests that pharmacological inhibition of H3K14ac may functionally mimic the effects of phospho-deficient HBO1, providing mechanistic insight into the anticancer activity of WM-3835.

### HBO1 regulates replication stress *via* Ser50/53 phosphorylation

**Figure 4:**
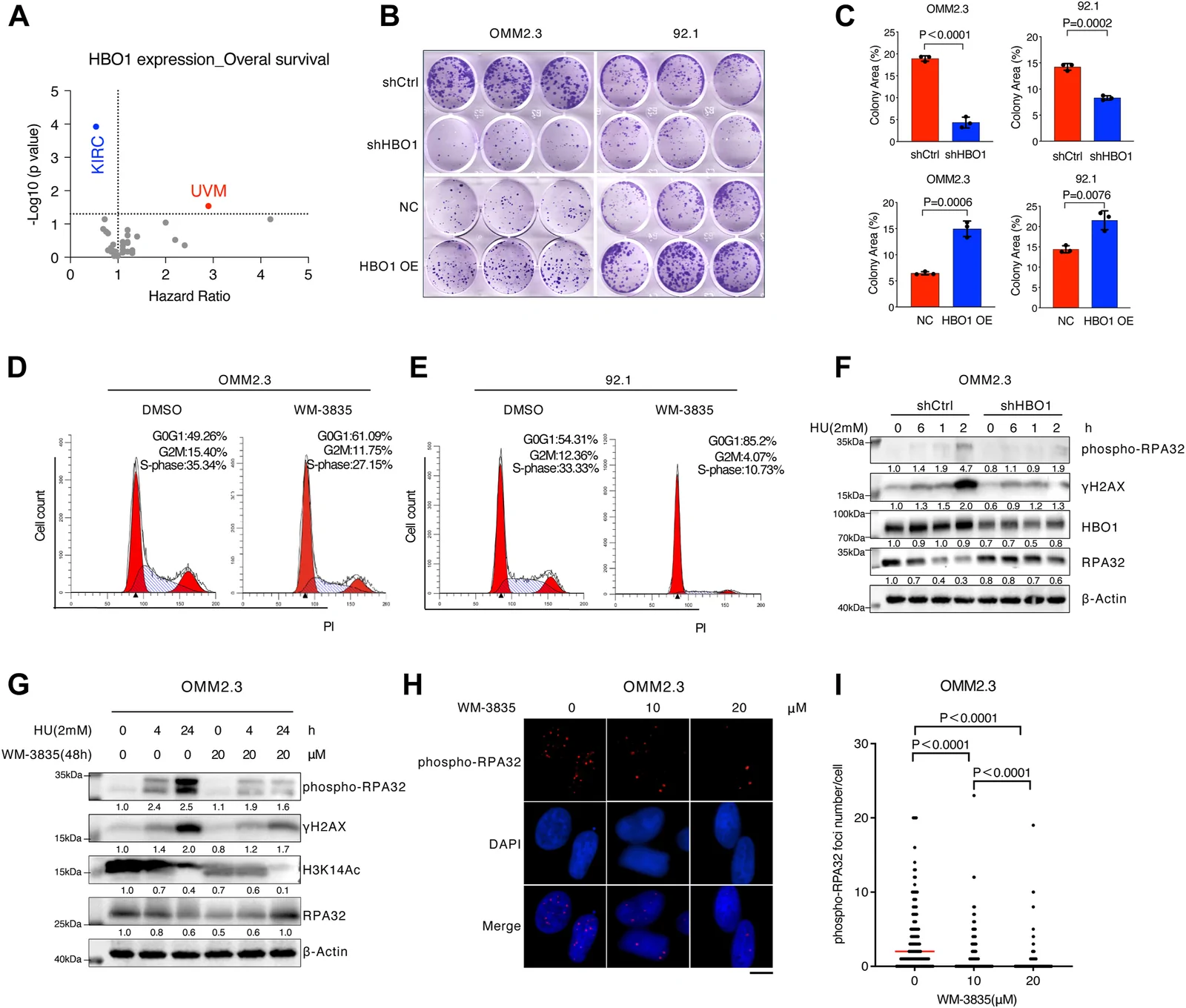
**HBO1 regulates replication stress *via* Ser50/53 phosphorylation.***A*, scatter plot showing the association between HBO1 expression and OS in 33 TCGA cohorts. Hazard ratios and -Log10(*p* value) were calculated using Cox proportional hazards model *via* the GEPIA website. Each *dot* represents one tumor cohort. *Dashed lines* indicate hazard ratios (HR) = 1 and -Log10(*p* value) = 1.301 (*p* = 0.05, statistical significance). *Red dot*: HR > 1 (high HBO1 associated with poor OS); *blue dot*: HR < 1 (high HBO1 associated with favorable OS). *B*, representative images of colony formation assays in OMM2.3 and 92.1 UM cell lines. *C*, quantitative analysis of relative colony area (n = 3 biological replicates; mean ± SD; unpaired two-tailed *t* test). *D* and *E*, cell cycle distribution analyzed by flow cytometry in OMM2.3 (*D*) and 92.1 (*E*) cells treated with 20 μM WM-3835 or vehicle control for 48 h. *F*, immunoblot analysis of RSR proteins in HBO1-knockdown *versus* control OMM2.3 cells treated with 2 mM HU for indicated durations. *G*, immunoblots of RSR pathway proteins in OMM2.3 cells treated with HU (2 mM) and/or WM-3835 (20 μM for 48 h). *H*, IF staining of phospho-RPA32 (S4/S8) in OMM2.3 cells treated with WM-3835 (0–20 μM for 48 h). The scale bar represents 10 μm. *I*, scatter plot quantifying the number of phospho-RPA32 foci per OMM2.3 cell under indicated conditions in (*H*). Each *dot* represents an individual cell; *horizontal lines* denote the median value (n = 142, 195 and 203 cells per group). Statistical significance was determined by the Kruskal–Wallis test followed by Dunn’s multiple comparisons test. HBO1, histone acetyltransferase binding to ORC1; HU, hydroxyurea; KIRC, Kidney Renal Clear Cell Carcinoma; RSR, replication stress response; TCGA, The Cancer Genome Atlas.

We then examined the expression of HBO1 and found that it is ubiquitously expressed in both normal (PIG1 and ARPE-19), and melanoma cell lines (Fig. S4*A*). Importantly, higher HBO1 expression levels were associated with poor overall survival (OS) in UM patients (Fig. 4*A*, Table S2). To study its function, we constructed HBO1 knockdown and overexpression stable OMM2.3 and 92.1 cell lines (Fig. S4, *B* and *C*). Compared with control cells, HBO1-depleted cells showed a remarkable decrease of ∼three-fourths in OMM2.3 and half in 92.1 in colony area. Consistently, upon HBO1-overexpression, OMM2.3 (*p* = 0.0006) and 92.1 (*p* = 0.0076) exhibited significantly increased colony formation ability (Fig. 4, *B* and *C*). These results support the oncogenic role of HBO1 in UM, warranting further analysis of cell cycle and replication stress responses.

In accordance with earlier reports (24, 26), flow cytometry analysis also showed G0/G1 cell cycle arrest by WM-3835 (Figs. 4, *D*, *E* and S5*A*). Given the interaction between HBO1 and ATR, we then examined the role of HBO1 in replication stress response. We calculated the replication stress response (RSR) score using the characteristic gene set of replication stress response (35). Pearson correlation analysis showed that HBO1 expression was significantly positively correlated with RSR score in various tissues (Fig. S6*A*, Table S3). ATR, CHK1, and RPA32 phosphorylation are markers of ATR pathway activation, while phosphorylated RPA32 (phospho-RPA32) and γH2AX are established replication stress markers (36). Under treatment with hydroxyurea (HU), phospho-RPA32 and γH2AX levels increased, confirming replication stress activation. Importantly, replication stress was significantly suppressed when HBO1 was knocked down or inhibited (Fig. 4, *F*–*I* and S6*B*), demonstrating the crucial role of HBO1 in replication stress responses.

**Figure 5:**
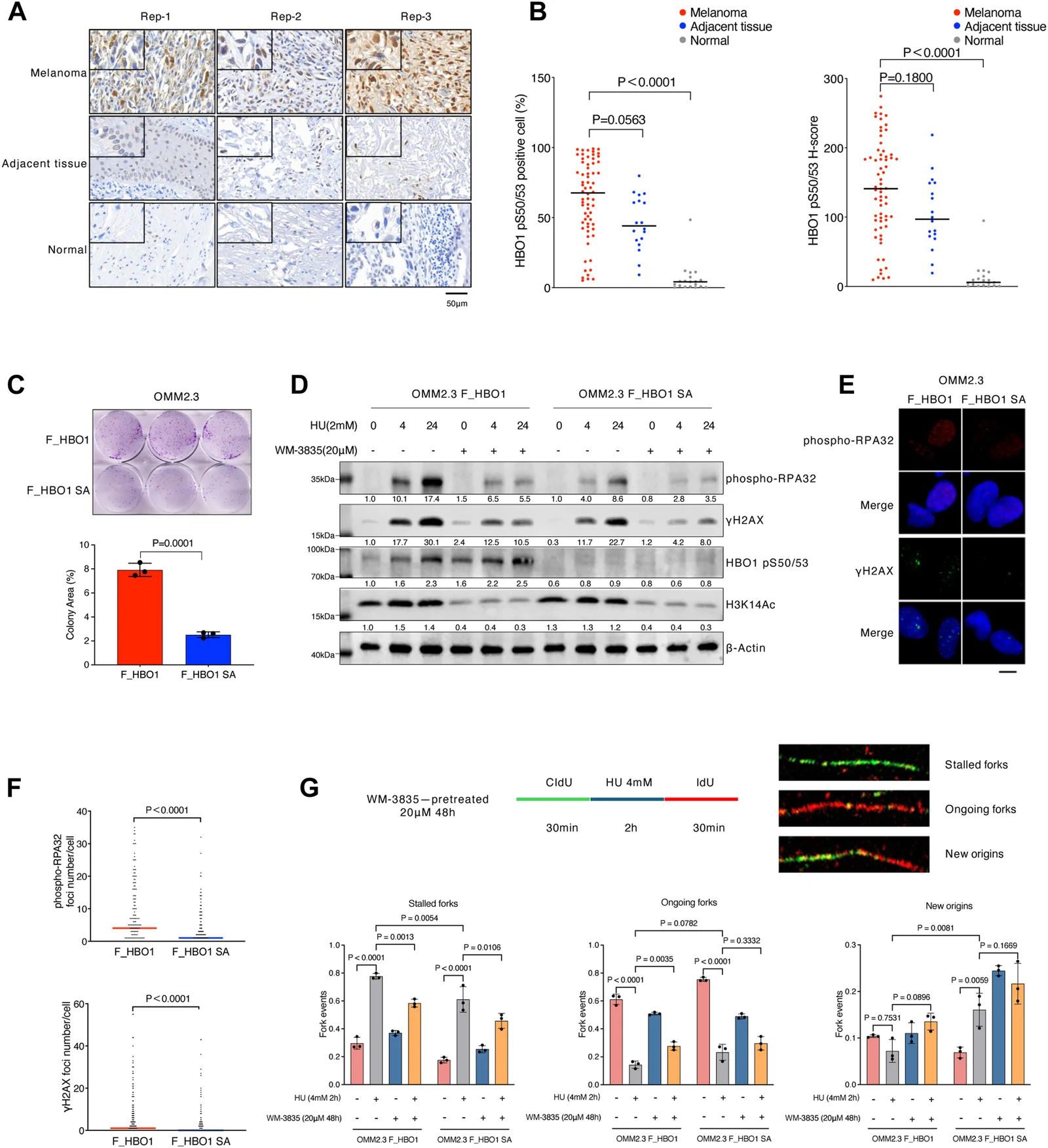
**Phosphorylation of HBO1 Ser50/53 is crucial for ATR signaling activation.***A*, representative IHC images of HBO1 pS50/53 in melanoma specimens, adjacent tissue, and normal skin (The scale bar represents 50 μm; insets: 2× magnification). *B*, scatter plot of HBO1 pS50/53 positivity and H-scores for indicated tissues. Each *dot* represents an individual tissue sample, and the *horizontal line* indicates the median value (n = 66, 18, and 19 samples per group). Statistical significance was determined by the Kruskal–Wallis test followed by Dunn’s multiple comparisons test. *C*, *top*: colony formation assays in OMM2.3 cells overexpressing Flag-tagged HBO1 or HBO1 S50/53A mutant. *Bottom*: quantified colony areas (n = 3; mean ± SD; unpaired two-tailed *t* test). *D*, immunoblot analysis of RSR proteins in OMM2.3 cells expressing F_HBO1 or F_HBO1 SA treated with HU (2 mM) ± WM-3835 (20 μM for 48 h). *E*, representative IF images of phospho-RPA32 and γH2AX in OMM2.3 cells expressing F_HBO1 or F_HBO1 SA (The scale bar represents 10 μm). *F*, quantification of DNA damage foci (phospho-RPA32: n = 156 and 203 cells; γH2AX: n = 186 and 191 cells). The *center line* shows the median. *p* values calculated using Mann–Whitney U test. *G*, DNA fibers from F_HBO1 or F_HBO1 SA expressed OMM2.3 cells. *Top*: schematic diagram of drug administration and staining procedures, and representative images of stalled forks, ongoing forks, and new origins. *Bottom*: quantitative statistics of stalled forks, ongoing forks, and new origins under different conditions (n = 3 technical replicates; mean ± SD; one-way ANOVA followed by *post hoc* multiple comparisons). ATR, Ataxia Telangiectasia and Rad3-related; HBO1, histone acetyltransferase binding to ORC1; HU, hydroxyurea; IHC, immunohistochemistry; RSR, replication stress response.

We next examined the clinical relevance of HBO1 phosphorylation at Ser50/53. Immunohistochemistry (IHC) staining of a melanoma tissue array revealed that HBO1 pS50/53 intensity was markedly elevated in melanoma, compared with adjacent normal and normal skin tissues (Fig. 5, *A* and *B*). Key genes involved in melanoma progression are often associated with cancer stages (Clark level) and Breslow thickness (37). However, in our data, patients diagnosed with advanced stages, greater Breslow thickness, and lymph node metastasis did not show higher HBO1 pS50/53 levels (Fig. S7*A*). Interestingly, inhibition of HBO1 pSer50/53 significantly reduced the colony area of OMM2.3 cells (Fig. 5*C*), without inducing cell cycle arrest (Fig. S7*B*). Both IF staining and Western blot showed remarkable reduction of phospho-RPA32 and γH2AX when the HBO1 Ser50/53 phosphorylation sites were mutated, suggesting that such phosphorylation is required for ATR signaling activation (Fig. 5, *D*–*F*). In addition, WM-3835 inhibited HU-induced replication fork arrest, while HBO1 Ser50/53 phosphorylation defect played a similar role (Fig. 5*G*). These findings are also consistent with the transcriptomic data in Figures 3, *I*–*K* and S2*F*, showing altered gene expression under WM-3835 treatment in cells reconstituted with HBO1 SA.

### Inhibition of ATR activity alters WM-3835 sensitivity

**Figure 6:**
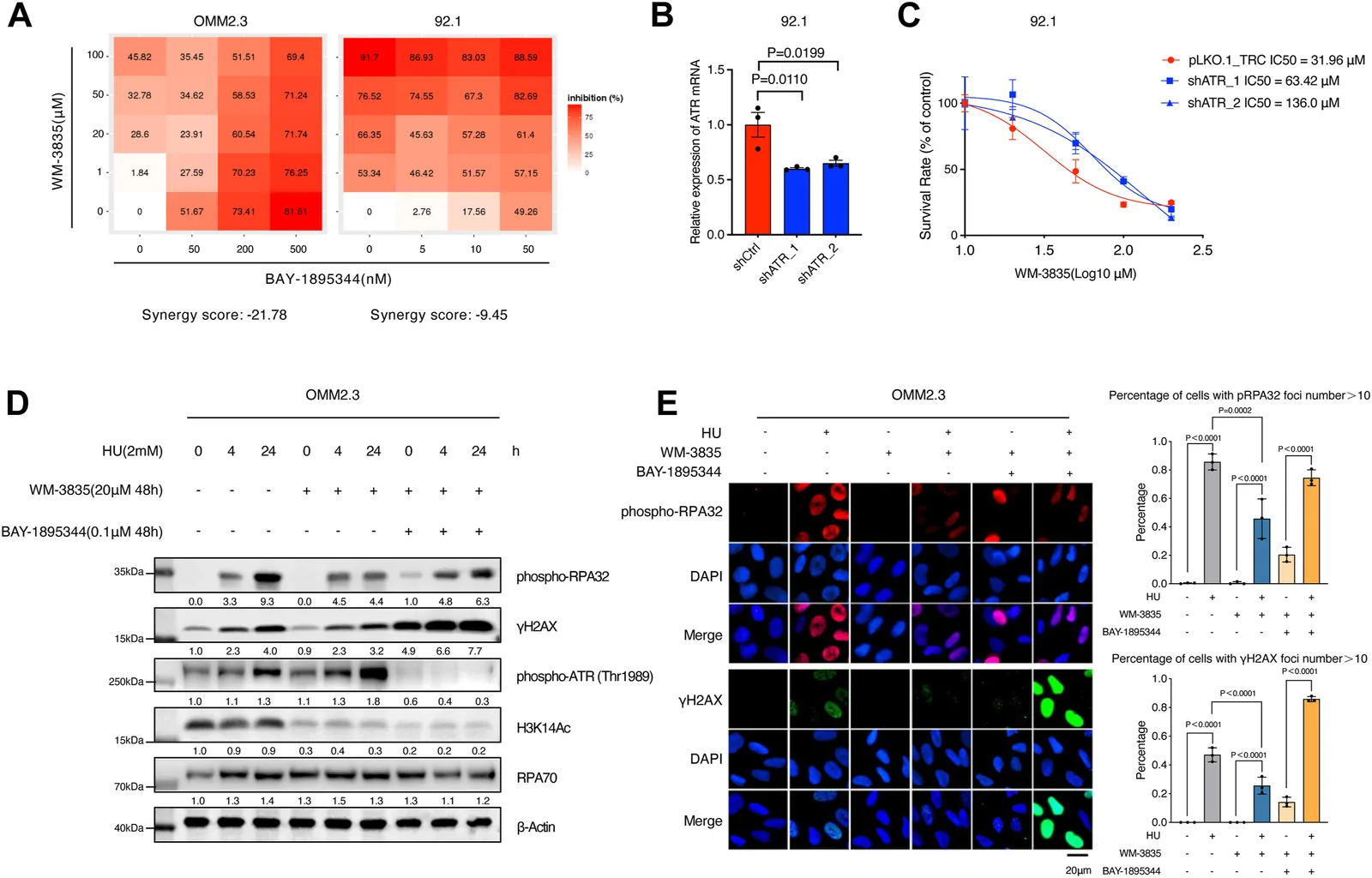
**Inhibition of ATR activity alters WM-3835 mediated phenotype.***A*, synergy analysis of WM-3835 (HBO1 inhibitor) and BAY-1895344 (ATR inhibitor) combination treatment in OMM2.3 and 92.1 cells. Heatmap shows combination inhibition rate. *B*, RT-qPCR validation of ATR knockdown efficiency in 92.1 cells. Gene expression was normalized to β-Actin (n = 3 technical replicates; mean ± SD; one-way ANOVA followed by *post hoc* multiple comparisons). *C*, cell viability assessed by CCK-8 assay in 92.1 cells with or without ATR knockdown treated with increasing concentrations of WM-3835 for 72 h (n = 3 biological replicates; mean ± SD). *D*, immunoblot analysis of RSR proteins in OMM2.3 cells treated with HU (2 mM, 0–24 h), WM-3835 (20 μM, 48 h), and/or BAY-1895344 (0.1 μM, 48 h). *E*, *left*: representative IF images of phospho-RPA32 (S4/S8) and γH2AX in OMM2.3 cells treated with HU (2 mM, 24 h), WM-3835 (20 μM, 48 h), and/or BAY-1895344 (0.1 μM, 48 h). The scale bar represents 20 μm. *Right*: quantification of cells with >10 phospho-RPA32 or γH2AX foci (n = 3 independent experiments; mean ± SD; one-way ANOVA followed by *post hoc* multiple comparisons). ATR, Ataxia Telangiectasia and Rad3-related; HBO1, histone acetyltransferase binding to ORC1; HU, hydroxyurea; RSR, replication stress response; RT-qPCR, real-time quantitative PCR.

Building on the previous results, we speculated that ATR activity may affect the treatment efficacy of WM-3835. We treated OMM2.3 and 92.1 cells with WM-3835 and BAY-1895344, an ATR inhibitor currently under clinical trial evaluation for the treatment of advanced solid tumors (38). Synergy score analysis demonstrated that the effects of WM-3835 were antagonized by the addition of BAY-1895344 (Figs. 6*A* and S8, *A* and *B*). Consistent with these observations, knockdown of ATR impaired the sensitivity of 92.1 cells to WM-3835 treatment (Fig. 6, *B* and *C*). We also examined the level of phospho-RPA32 and γH2AX, to evaluate the impact on replication stress by BAY-1895344 and WM-3835. We found that the inhibition of phospho-RPA32 and γH2AX by WM-3835 was significantly attenuated under BAY-1895344 treatment (Fig. 6, *D* and *E*). These data support the role of ATR as a potential predictive biomarker for WM-3835 sensitivity.

## Discussion

The histone acetyltransferase HBO1 has been predominantly characterized as exerting its biological functions through its catalytic acetylation activity (39, 40, 41). While previous studies have established that HBO1 targeting induces tumor cell death *via* cell cycle arrest (26), our work significantly expands this understanding by identifying ATR as a novel binding partner and critical regulator of HBO1. This discovery functionally links the replication stress regulation function of ATR with the chromatin regulation function of HBO1.

The 611-amino acid HBO1 protein features two principal domains: an N-terminal DNA-binding domain containing a zinc finger motif (zf-C2HC, 184–212aa) that interacts with replication initiation factors MCM2 and ORC1 (42), and a C-terminal MYST acetyltransferase domain (390–577aa) responsible for catalytic activity (27). Despite its functional importance, the NTD remains poorly characterized due to the lack of structural information. Our findings substantially advance the understanding of the HBO1 NTD.

Emerging evidence indicates that posttranslational modifications of the HBO1 NTD serve as molecular switches modulating its activity. For instance, CDK1-mediated phosphorylation at Thr85/Thr88 primes subsequent PLK1-dependent phosphorylation at Ser57, facilitating prereplicative complex formation (32, 43). In addition, CDK11-mediated HBO1 phosphorylation enhances its acetyltransferase activity (30). In line with these findings, our study demonstrates that prevention of Ser50/53 phosphorylation dramatically altered the genomic binding landscape of HBO1 (Fig. 3). The phospho-deficient mutant (HBO1 SA) exhibited substantially increased genomic occupancy (23,539 binding genes *versus* 5998 in WT), suggesting that phosphorylation at these sites normally restricts widespread DNA binding.

Notably, motif analysis of HBO1 SA binding regions revealed strong enrichment for CTCF motifs. Given the established role of CTCF in maintaining chromatin accessibility (34), this observation may explain the expanded genomic binding of the phospho-deficient mutant. Furthermore, while WT HBO1 preferentially associates with TSSs and promoter regions, consistent with reports in human embryonic stem cells (hESCs) (33) and HeLa cells (44), the SA mutant showed increased binding at replication origins (Fig. 3, *A*–*C*) and promoted the formation of nascent replication forks (Fig. 5*G*), suggesting phosphorylation-dependent regulation of replication licensing.

Our findings uncover a novel ATR-HBO1 axis in replication stress management. We demonstrate that ATR-mediated phosphorylation of HBO1 Ser50/53 modulates its binding to DNA damage repair genes, independent of its acetyltransferase activity. Intriguingly, the transcriptional effects of HBO1 SA mutation paralleled those of WM-3835 treatment, both downregulating DNA repair and cell cycle-related genes. Mechanistically, HBO1 SA exhibits a putative physical association with *TOPBP1* and *RPA2*, suggesting it might impair the activity of these essential mediators of ATR signaling (45, 46). This observation explains how ATR-dependent HBO1 phosphorylation promotes replication stress response: 1) RPA-coated ssDNA recruits ATR-ATRIP complexes; 2) TOPBP1 mediates ATR activation; 3) Activated ATR phosphorylates HBO1 Ser50/53; 4) Phospho-HBO1 upregulates DNA repair genes through targeted genomic binding. This feedforward mechanism ensures appropriate cellular responses to replication stress.

While WM-3835 (a first-in-class HBO1 inhibitor) shows promise in inducing G0/G1 arrest in several tumor types (24, 47), our work reveals its functional interplay with ATR signaling. Surprisingly, ATR inhibition attenuated WM-3835 efficacy, implying cellular ATR levels could act as a candidate *in vitro* biomarker to gauge cellular responsiveness toward HBO1-targeted therapy. This observation bears preliminary translational hints: combinations of WM-3835 with established DDR checkpoint inhibitors (targeting ATR, CHK1, WEE1, and MYT1) (48)—a common therapeutic strategy for DDR-dependent malignancies—require extensive preclinical testing to inform patient stratification in future clinical trials.

Taken together, our study establishes ATR as a key regulator of HBO1 through phosphorylation-dependent modulation of its genomic binding. This ATR-HBO1 axis represents a novel mechanism coordinating chromatin regulation with replication stress response. From a translational perspective, our *in vitro* findings nominate ATR expression as a candidate biomarker for cellular sensitivity to HBO1 inhibitors at the preclinical stage, laying the groundwork for follow-up preclinical and translational research toward future precision therapeutic development.

## Experimental procedures

### Cell culture

Human renal epithelial cell line HEK293T and human cutaneous melanoma cell lines A375 and A2058 cells were cultured in Dulbecco’s modified Eagle’s medium (Gibco, #8122522). Human cutaneous melanocyte cell PIG1 and retinal pigment epithelium cell ARPE-19 were cultured in RPMI1640 medium (Gibco, #8122322). Human cervical cancer cell HeLa and Human UM cell lines (OMM1, OMM2.3, MEL202, MEL270, MEL290, 92.1, MUM2B) were cultured in RPMI1640 medium (Gibco, #8122322). All media were supplemented with 10% fetal bovine serum (Gibco, #16140071) and 1% antibiotics (Penicillin-streptomycin, Gibco, #10378016). All cells were cultured in a 37 °C humidified incubator containing 5% CO_2_. The cell lines used in this study were authenticated by short tandem repeat profiling and confirmed mycoplasma-free.

### Western blotting

Total cell lysates were prepared in urea lysis buffer supplemented with protease inhibitor and phosphatase inhibitor. Proteins were separated by sodium dodecyl sulfate-polyacrylamide gel electrophoresis and transferred to polyvinylidene fluoride membranes (Millipore, #ISEQ00010). After blocking with 5% nonfat milk for 1 h at RT, the membranes were incubated with anti-HBO1 (Proteintech, 13751-1-AP, 1:2000), anti-ATR (Abclonal, A21253, 1:1000), anti-myc (Santa cruz, sc-40, 1:1000), anti-Flag (Proteintech, 66008-4-Ig, 1:10,000), anti-Phospho-ATM/ATR Substrate (S/TQ) (CST, 9607S, 1:1000), anti-HBO1 pS50/53 (1:500), anti-phospho-RPA32 (BETHYL, A300-245A, 1:2000), anti-γH2AX (SIGMA, 05-636-I, 1:1000), anti-RPA32 (CST, 35869S, 1:1000), anti-H3K14Ac (Abclonal, A7254, 1:1000), anti-phospho-ATR(CST, #58014, 1:1000), anti- RPA70 (CST, #2267S, 1:1000) antibodies overnight at 4 °C and then with the appropriate secondary antibodies conjugated to a fluorescent tag (CST, #5151 and #5470, 1:10,000) for 1 h at RT. Anti-β-Actin (Proteintech, 66009-1-Ig, 1:20,000), anti-β-tubulin (Abclonal, A12289, 1:10,000) and anti-GAPDH (Proteintech, 60004, 1:20,000) antibodies served as loading controls. The immunoblots were recorded with the Odyssey infrared imaging system (LI-COR Biosciences). Relative band intensity was quantified under each band. Full length uncropped original western blots are provided in supporting Information.

### Co-IP

Cells were pelleted and washed with PBS, then lysed in 500 μl lysis buffer (120 mM NaCl, 20 mM Tris-Cl, 2 mM EDTA, 1% NP-40, and 5% glycerol) supplemented with 1× protease inhibitor cocktail. An aliquot corresponding to 3% of the total cell lysate was reserved as the input sample before immunoprecipitation. Remaining cell lysates were incubated overnight at 4 °C with anti-HBO1 (Proteintech, 13751-1-AP, 4 μl), anti-ATR (CST, 13934S, 2.5 μl), anti-Myc (Santa Cruz, sc-40, 5 μl), anti-Flag (Proteintech, 66008-4-Ig, 5 μl), or normal mouse IgG control (Santa Cruz, sc-2025, 1:10,000). A total of 30 μl Protein A magnetic beads (CST, #73778) were subsequently added for an additional 2 h incubation, followed by three washes with lysis buffer. Protein complexes were eluted from beads using either 100 μl glycine solution for IP-MS analysis or 1× SDS loading buffer (NCM, WB2001) for sodium dodecyl sulfate-polyacrylamide gel electrophoresis.

All subsequent LC-MS/MS detection and proteomic data acquisition services were completed by APTBIO (Shanghai Applied Protein Technology Co., Ltd). For IP-MS sample processing, eluted protein complexes were denatured, reduced with DTT, alkylated with IAA, desalted *via* Zeba Spin columns, and digested with trypsin at a 1:50 enzyme:protein mass ratio at 37 °C for 20 h. Desalted and vacuum-dried peptides were reconstituted in 0.1% formic acid, separated on an Easy-nLC 1000 UHPLC system (Thermo Fisher Scientific) with mobile phase A (0.1% aqueous formic acid) and mobile phase B (0.1% formic acid in 84% acetonitrile), and loaded onto a trap column preequilibrated with 95% mobile phase A. Mass spectrometry detection was performed on a QExactive Orbitrap mass spectrometer (Thermo Fisher Scientific) in positive-ion data-dependent acquisition mode with HCD fragmentation. MS1 full scans covered m/z 300 to 1800 at 70,000 resolution (m/z 200), AGC target 1 × 10^6^, maximum injection time 50 ms, and 30 s dynamic exclusion; each full scan triggered up to 20 MS2 scans with a 2 m/z isolation window, normalized collision energy 27 eV, MS2 resolution 17,500 (m/z 200), and 0.1% underfill threshold. Raw MS files were processed with MaxQuant v1.6.14 to generate peak lists and conduct label-free iBAQ quantification against the UniProt *Homo sapiens* database (version 204,318, released 08 Jan 2024, human taxonomy). Unique and razor peptides were classified and counted during quantitative analysis. The false discovery rate for peptide and protein identifications was limited to 1% using the Benjamini–Hochberg correction as the acceptance threshold for all valid peptide and protein assignments. Functional enrichment analysis of proteins enriched in immunoprecipitation samples was performed using DAVID online tool (https://davidbioinformatics.nih.gov/summary.jsp).

### Transfection and virus packaging

Two shRNA sequences targeting HBO1 and ATR were cloned into the pLKO.1_TRC vector. The HBO1 targeting sequences were: 5′-CCTCTCAAGTAGCTGGGATTA-3′(shHBO1_1); 5′-TGGAACCGAAGATTCCGATTT-3′ (shHBO1_2). The ATR targeting sequences were: 5′-CCGGATACTTACAGATGTAAA-3′(shATR_1); 5′-GTAATGCATTTGGTATGAATC-3′ (shATR_2).

The full length of human HBO1 (isoform NM_007067), myc_HBO1 D1, myc_HBO1 D2 and myc_HBO1 D3 were cloned into the pHAGE-myc-puro vector respectively for overexpression of HBO1 and HBO1 D1/2/3. Primers used for cloning are shown in Table S4. CMV_Flag_ATR (#41909) was purchased from addgene. PolyJet DNA In Vitro Transfection Reagent (SignaGen, SL100688) was used for plasmid transfection following the manufacturer’s instructions. After lentiviral packaging with HEK293T cells, cells were infected with lentiviruses and selected by incubation with 2 μg/ml puromycin for 3 days.

### IF

Cells adhering to a glass slide were fixed with 4% paraformaldehyde (Biosharp, #70071800) for 15 min, permeabilized with 0.1% Triton X-100 (Sigma-Aldrich, T9284) for 15 min and then blocked with 2% bovine serum albumin solution for 1 h at RT. After incubation with primary antibody against HBO1 pS50/53 (1:200), phospho-RPA32 (BETHYL, A300-245A, 1:2000), γH2AX (SIGMA, 05-636-I, 1:500), Flag (ABclonal, AE005, 1:200) and HBO1 (Proteintech, 13751-1-AP, 1:200) overnight, the cells were washed three times with PBS and subsequently incubated with Alexa Fluor 594 secondary antibody (Invitrogen, A11012, 1:1000) or Alexa Fluor 488 secondary antibody (Invitrogen, A11029, 1:1000) for 1 h. The coverslips were then mounted with ProLong Gold mounting medium with DAPI (Invitrogen, P36931) and observed under an inverted fluorescence microscope. Foci number was quantified for each condition. The results presented were obtained from three independent biological replicates; at least 50 cells were measured per replicate.

### RNA extraction and quantification

RNA purification was performed using EZ-press RNA purification kit (EZBioscience, #B0004DP) according to the manufacturer’s guidelines. Then, cDNA synthesis was achieved using the HiScript III RT SuperMix (Vazyme, #R323). Finally, real-time quantitative PCR was carried out using ChamQ Universal SYBR qPCR Master Mix (Vazyme, #Q711). mRNA expression values were calculated using the ΔΔCt method and human β-Actin gene as a control. All primer sequences used for real-time quantitative PCR are listed in Table S5, and raw quantitative PCR data are provided in the supporting information.

### RNA sequencing (RNA-seq)

RNA-seq was performed by Novogene. Total RNA was harvested using Trizol reagent (Invitrogen), and the integrity of the RNA was assessed by Agilent 5400 bioanalyzer (Thermo Fisher Scientific). Only high-quality RNA samples were used for subsequent sequencing, and approximately 1 μg mRNA from each sample was used for RNA sequencing on the Illumina HiSeq PE150 platform with a sequencing depth of 6 G. Each experimental group was performed with 2 biological replicates.

Raw sequencing reads were filtered to remove low-quality reads (Q-score < 20), adapter sequences, and contaminating reads. Clean reads were mapped to the reference genome using Hisat2 (v2.2.1), and gene expression levels were quantified as fragments per kilobase of transcript per million mapped reads using featureCounts (v1.5.0-p3). Differential expression analysis was performed using DESeq2 v1.20.0 with a custom group-based design matrix. Raw counts were normalized by the median-of-ratios approach, and *p*-values were adjusted *via* the Benjamini-Hochberg false discovery rate method. Genes with |log2(fold change)| ≥ 0.585 and adjusted *p* < 0.05 were identified as DEGs. GO enrichment of DEGs was analyzed using the DAVID online tool (https://davidbioinformatics.nih.gov/summary.jsp).

### CUT&Tag

CUT&Tag assay was performed using Hyperactive In-Situ ChIP Library Prep Kit for Illumina (Vazyme, TD904) following the manufacturer’s protocol. Briefly, 1 × 10^5^ OMM2.3 cells transfected with F_HBO1 or F_HBO1 SA were collected. After binding to concanavalin A–coated magnetic beads (ConA beads), bead-bound cells were incubated with anti-Flag antibody (CST, #14793, 1:50) or normal rabbit IgG (CST, 2729, 1:100) for 2 h at RT. After brief wash with dig-wash buffer, cells were then incubated with goat anti rabbit secondary antibody (Abcam, ab6702, 1:100) for 1 h at RT. When antibody binding procedures were finished, the bead-bound cells were then mixed with hyperactive pA-Tn5 transposon and tagmentated with tagmentation buffer. Tagmentated DNA was then extracted and amplified to form the sequencing-ready libraries. After the PCR reaction, libraries were purified with the DNA clean beads (Vazyme, N411) and library quality was assessed on the Agilent Bioanalyzer 2100 system. The clustering of the index-coded samples was performed on a cBot Cluster Generation System using TruSeq PE Cluster Kit v3-cBot-HS (Illumina).

The library sequencing was performed by Novogene. The sequencing depth was 6 G per sample on Illumina Novaseq platform. Raw reads were first quality-checked using FastQC (v0.11.3), then trimmed with fastp (0.20.0) (49) with the following parameters: discarding reads with >40% bases of quality <15, >6 N bases, residual adapter sequences, or length <15 bp after trimming; clean reads were reevaluated by FastQC. Clean reads were aligned to the reference genome using BWA (0.7.12) (50), and only reads with MAPQ (Mapping Quality) >13 (uniquely mapped reads) were retained for subsequent analysis. The density of total mapped reads on each chromosome (stratified by strand) was counted, and CUT&Tag signals 3 kb upstream and downstream of TSS were analyzed using deepTools (3.0.2) with 50 bp bins to calculate average signal intensity; alignment results were visualized *via* IGV_2.19.1. Fragment sizes were predicted using ATACseqQC (https://bioconductor.org/packages/release/bioc/vignettes/ATACseqQC/inst/doc/ATACseqQC.html). Peak calling was performed on uniquely mapped reads using MACS2 (2.1.0) (51), with q values calculated *via* Poisson distribution to filter false positives; peaks were statistically analyzed (number, width, genomic distribution) and associated genes were annotated. Peak functional annotation was conducted using ChIPseeker (52), with priority assignment to functional regions as promoter > 5′UTR > 3′UTR > Exon > Intron > Downstream > Intergenic (53). Differential peaks were defined as those with RPM ratio >2 between groups, and GO enrichment analysis was performed on their associated genes. Motif sequences were identified using Homer (findMotifsGenome.pl v4.9.1) (50) based on 500 bp sequences (250 bp upstream and downstream of peak summits). GO analysis was implemented by DAVID online tool (https://davidbioinformatics.nih.gov/). In Venn diagrams, numbers represent genes co-occurring between conditions.

### Colony formation

A volume of 3 ml of complete medium containing 2000 cells was seeded in each well of a 6-well plate and incubated for about 2 weeks. Once colonies formed in control conditions, plates were fixed with 4% paraformaldehyde, stained with crystal violet. Colony formation efficiency is indicated by colony area, and for quantitative analysis, the colony area was measured using ImageJ software, and the relative colony area percentage (colony area/total plate area × 100%) was calculated. The quantifications presented were obtained from three independent biological replicates.

### Cell viability assay

Cell viability was determined using a CCK-8 assay to evaluate the sensitivity of cells to WM-3835 (Selleck, S9805) and BAY-1895344 (Selleck, S8666) treatment. Briefly, cells were seeded into 96-well plates at a density of 1000 cells per well, with three technical replicates set for each treatment condition. After seeding, cells were allowed to adhere overnight. Subsequently, cells were treated with different concentrations of WM-3835 and BAY-1895344 (either alone or in combination, as per experimental design) and incubated under the same culture conditions. At the end of the incubation period, CCK-8 reagent (NCM Biotech) was added to each well following the manufacturer’s instructions, and the plates were further incubated for an appropriate time to allow color development. The absorbance at 450 nm was measured. All data were recorded and analyzed, and the quantifications presented were derived from three independent biological replicates to ensure experimental reproducibility. The synergistic effect between WM-3835 and BAY-1895344 was evaluated by calculating the synergy score using SynergyFinder software (https://synergyfinder.aittokallio.group/20250411123730439716/); a synergy score less than −10 indicates a likely antagonistic interaction between the two drugs.

### Cell cycle assay

Cell cycle distribution of melanoma cells with or without WM-3835 treatment was detected by flow cytometry with propidium iodide staining. Briefly, melanoma cells were seeded at a density of 2 × 10^5^ cells per sample in each group, with 2 biological replicates. After treatment with WM-3835 (or dimethyl sulfoxide as the vehicle control), cells were harvested by digestion with 0.25% trypsin-EDTA (Gibco), followed by two washes with ice-cold phosphate-buffered saline (PBS, pH 7.4; Gibco) *via* centrifugation at 1000 rpm for 5 min. Cells were then fixed with 75% cold ethanol (precooled at −20 °C) at 4 °C in the dark overnight. Prior to staining, fixed cells were washed twice with PBS to remove residual ethanol, and then incubated with PI/RNase Staining Buffer (Multi sciences, CCS012) in the dark at RT for 30 min. The cell cycle distribution (G0/G1, G2/M and S phases) was analyzed using a flow cytometer (BD FACS calibur), and DNA content distribution was analyzed with FlowJo software.

### IHC

Tissue microarrays (ZL-MEL361 and ZL-MEL963) for IHC staining were bought from Weiaobio. The information of tissue microarrays is provided in Table S6. IHC staining of tissue microarrays was performed by Servicebio. Tissues were deparaffinized and rehydrated through an alcohol series, followed by antigen retrieval with sodium citrate buffer. Then tissue sections were blocked with 3% bovine serum albumin 30 min at RT and then incubated with anti-HBO1 pS50/53 (1:200) antibody at 4 °C overnight. Phospho Ser50 and Ser53 HBO1 rabbit polyclonal antibodies were generated by HUABIO. Finally, the tissues were covered with horseradish peroxidase-labeled secondary antibody and incubated at RT for 50 min. All immunostained slides were scanned on 3D Histech Quant Center (3D Histech), and computerized image analysis was performed by Halo (Indica labs). Immunostaining for HBO1 pS50/53 was analyzed in melanoma tissues, adjacent normal tissues and normal skin tissues using percentage of positive cells and histochemistry score (H-score). H-score was determined based on the proportion of positive cells and the intensity of nuclear staining as previously described (54). H-score = ∑(pi × i)= (percentage of cells of weak intensity × 1) + (percentage of cells of moderate intensity × 2)+ (percentage of cells of strong intensity × 3). In the formula, pi represents the percentage of positive cells in the slide; i represents the intensity of HBO1 pS50/53 staining.

### DNA fibers

Cells were seeded in 6-well plates for routine culture. For drug intervention, cells in the WM-3835 treatment group were pretreated with 20 μM WM-3835 for 48 h before the replication fork tracking assay. Subsequently, the cultured cells were sequentially labeled with different thymidine analogs to mark newly synthesized DNA strands. First, cells were incubated with 20 μM CldU (Sigma-Aldrich, C6891) for 30 min. For HU treatment groups, after CldU labeling, cells were further treated with 4 mM HU and incubated for 2 h to induce replication stress. Thereafter, cells were pulse-labeled with 100 μM IdU (MCE, HY-B0307) for another 30 min. After the completion of dual labeling, all cells were harvested, and DNA fiber spreads were prepared as previously described (55). CldU was detected first with the rat anti-BrdU antibody (Abcam, ab6326, 1:200) and IdU with the mouse anti-BrdU antibody (BD Biosciences Pharmingen, 347580, 1:100). Secondary antibodies were FITC-conjugated goat anti-rat IgG (H + L) (ABclonal, AS019) and Cy3-conjugated goat anti-mouse IgG (H + L) (ABclonal, AS008), respectively. Fluorescent signals of DNA fibers were visualized and imaged *via* fluorescence microscope (Nikon). Three types of replication forks were quantified based on fluorescent signals: ongoing forks (dual CldU/IdU signals), stalled forks (only CldU signals), and new replication origins (only IdU signals). More than 70 valid replication forks were counted per group per independent experiment, with all analyses performed in three biological replicates.

### Statistical analysis

GraphPad Prism 11.0 was used for statistical analyses. Descriptive values are presented as mean ± SD unless stated elsewhere. Statistical differences between two groups were assessed using the unpaired Student’s *t* test for normally distributed data, while the Mann–Whitney U-test was applied when the data did not pass the normality test. For multiple group comparisons, if the data were normally distributed, ANOVA followed by Tukey’s *post hoc* multiple comparison test was performed; if the data did not follow a normal distribution, the Kruskal–Wallis test was used for overall comparison among multiple groups, followed by Dunn’s multiple comparisons test as a *post hoc* analysis to assess pairwise differences. Detailed information of statistical tests is described in the figure legends. All statistical *p*-values are directly marked in the figures and presented with four decimal places. Results were considered statistically significant when *p* < 0.05. TCGA OS analysis and Pearson correlation analysis (HBO1 *versus* RSR signature score) was performed on GEPIA (http://gepia.cancer-pku.cn/). RSR signature gene set was shown in Table S7.

## Ethics statement

The study was performed in accordance with World Medical Association Declaration of Helsinki and was approved by the Ethics Committee of Shanghai Jiao Tong University.

## Data availability

The mass spectrometry proteomics data are provided in Table S1 and deposited to the PRIDE repository (ProteomeXchange Consortium) under accession PXD081136. RNA-seq and CUT&Tag data have been deposited to Gene Expression Omnibus with accession number GSE299365 and GSE299366.

## Supporting information

This article contains supporting information.

## Conflict of interest

The authors declare that they have no conflicts of interest with the contents of this article.
