# Supplementary material for: ATR-dependent phosphorylation of the histone acetyltransferase HBO1 suppresses chromatin binding and promotes replication stress responses
Source: J Biol Chem. 2026 Aug 10;302(10):113419. doi: 10.1016/j.jbc.2026.113419 (PMC13570308; doi:10.1016/j.jbc.2026.113419)
Supplement: Supplementary file 1 [file mmc1.pdf]

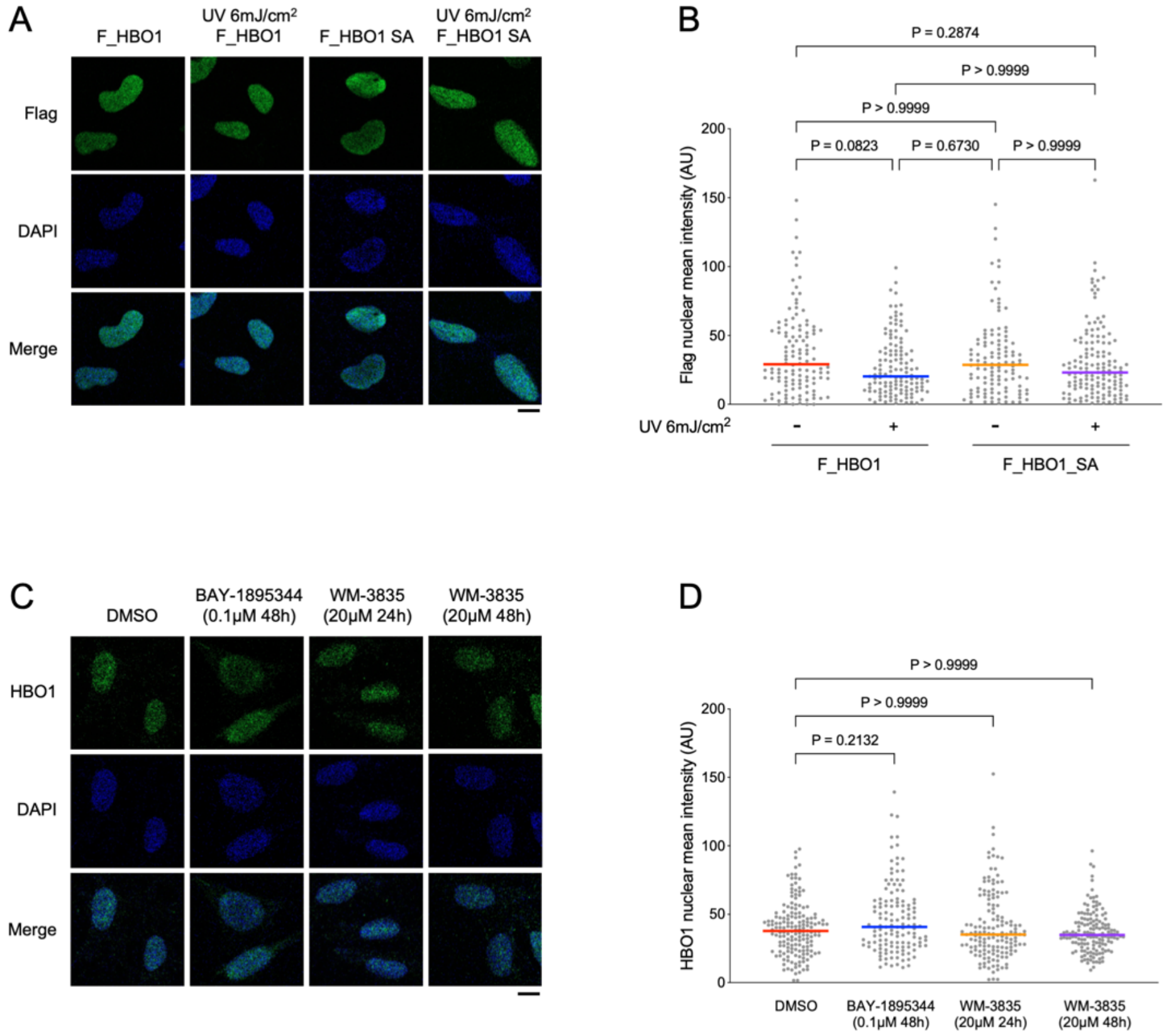

Fig. S1 IF staining in OMM2.3 cells.

(A) IF of Flag staining in OMM2.3 cells expressing F_HBO1 or F_HBO1 SA, with or without UV irradiation (6 mJ/cm^2). Cells were fixed 1 h post-irradiation. Scale bar: 10 µm. **(B)** Scatter plot quantifying the mean nuclear Flag intensity per OMM2.3 cell under indicated conditions in **(A)**. Each dot represents an individual cell; horizontal lines denote the median value (n = 122, 129, 117 and 143 cells per group). **(C)** IF of HBO1 staining in OMM2.3 cells treated with DMSO, BAY-1895344, or WM-3835. **(D)** Scatter plot quantifying the mean nuclear HBO1 intensity per OMM2.3 cell under indicated conditions in **(C)**. Each dot represents an individual cell; horizontal lines denote the median value (n = 167, 133, 150 and 150 cells per group). Statistical significance was determined by the Kruskal-Wallis test followed by Dunn's multiple comparisons test. AU, arbitrary units.

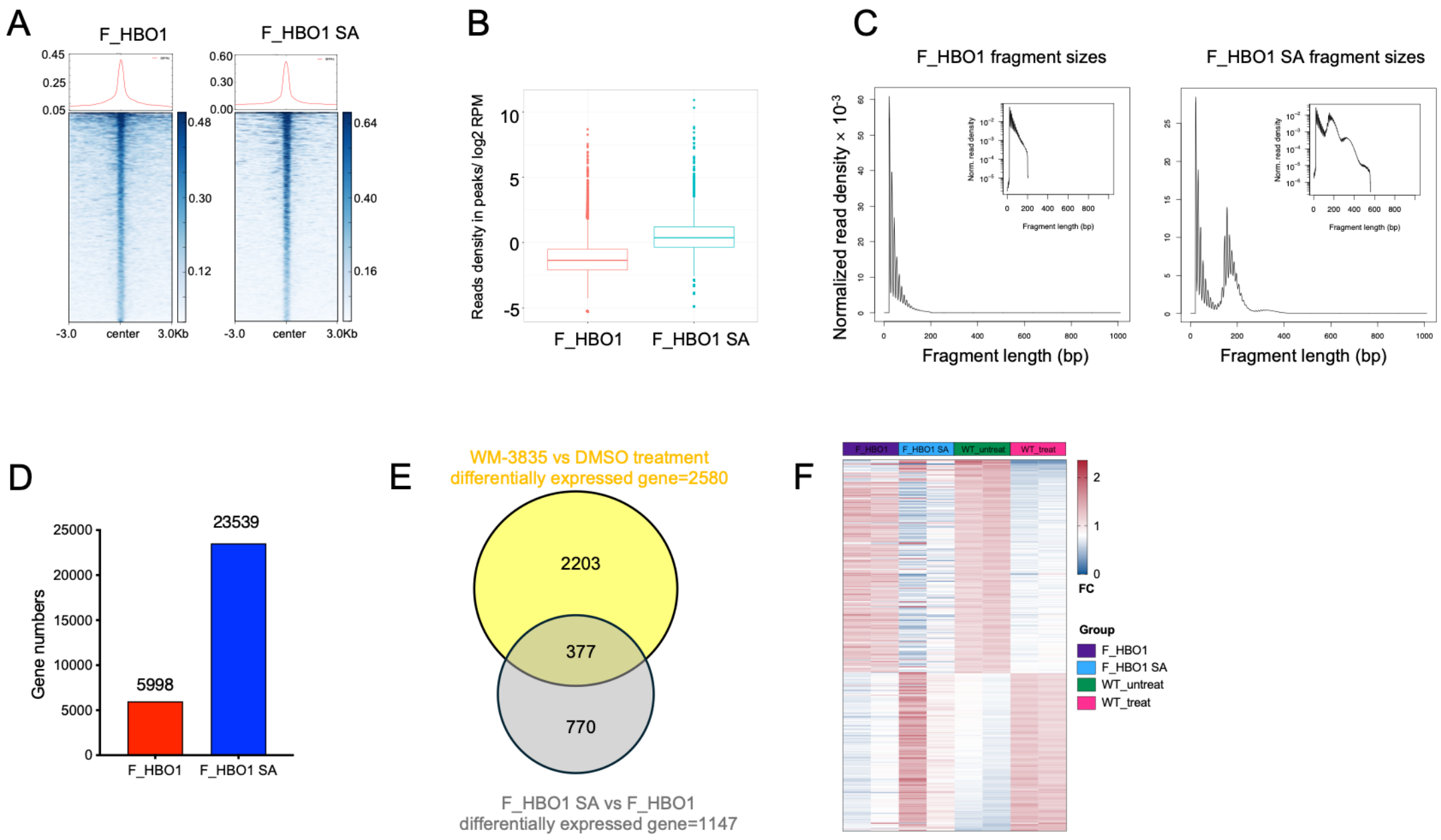

Fig. S2 CUT&Tag and RNA-seq analysis of F_HBO1 and F_HBO1 SA expressed OMM2.3 cells.

(A) Signal intensities and heatmap analysis of Flag-tagged HBO1 and HBO1 SA at the peak center (± 3 kb) in OMM2.3 cells. **(B)** Read density distribution of CUT&Tag signals for F_HBO1 and F_HBO1 SA. **(C)** Fragment size distribution of F_HBO1 and F_HBO1 SA samples. **(D)** Bar graph showing the number of genes associated with F_HBO1 and F_HBO1 SA in OMM2.3 cells. **(E)** Venn diagram illustrating the overlap between differentially expressed genes in F_HBO1 SA vs. F_HBO1 (gray) and differentially expressed genes after 48 hours of treatment with 20 μM WM-3835 (yellow). **(F)** Heatmap of gene expression for the 377 overlapping differentially expressed genes identified in **(E)**. Data were normalized to pairwise WT_untreated or F_HBO1 cells and represent the average of two replicates per condition.

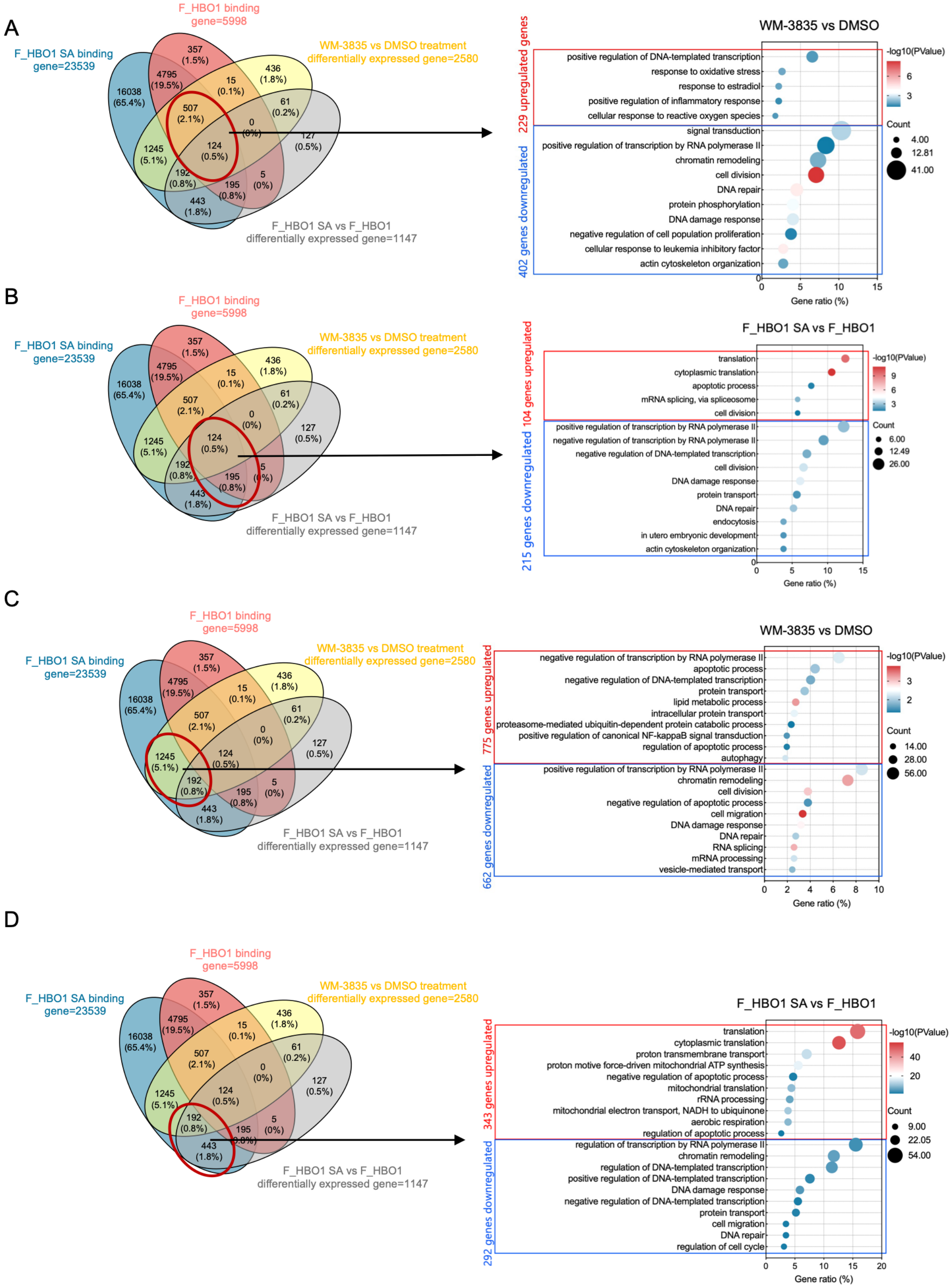
A
F_HBO1 binding gene=5998
F_HBO1 SA binding gene=23539
WM-3835 vs DMSO treatment differentially expressed gene=2580
F_HBO1 SA vs F_HBO1 differentially expressed gene=1147
16038 (65.4%)
4795 (19.5%)
357 (1.5%)
15 (0.1%)
436 (1.8%)
507 (2.1%)
0 (0%)
61 (0.2%)
1245 (5.1%)
124 (0.5%)
127 (0.5%)
192 (0.8%)
195 (0.8%)
5 (0%)
443 (1.8%)
WM-3835 vs DMSO
229 upregulated genes
402 genes downregulated
positive regulation of DNA-templated transcription
response to oxidative stress
response to estradiol
positive regulation of inflammatory response
cellular response to reactive oxygen species
signal transduction
positive regulation of transcription by RNA polymerase II
chromatin remodeling
cell division
DNA repair
protein phosphorylation
DNA damage response
negative regulation of cell population proliferation
cellular response to leukemia inhibitory factor
actin cytoskeleton organization
-log10(PValue)
6
3
Count
4.00
12.81
41.00
0 5 10 15
Gene ratio (%)
B
F_HBO1 SA vs F_HBO1
104 genes upregulated
215 genes downregulated
translation
cytoplasmic translation
apoptotic process
mRNA splicing, via spliceosome
cell division
positive regulation of transcription by RNA polymerase II
negative regulation of transcription by RNA polymerase II
negative regulation of DNA-templated transcription
cell division
DNA damage response
protein transport
DNA repair
endocytosis
in utero embryonic development
actin cytoskeleton organization
-log10(PValue)
9
6
3
Count
6.00
12.49
26.00
0 5 10 15
Gene ratio (%)
C
WM-3835 vs DMSO
775 genes upregulated
662 genes downregulated
negative regulation of transcription by RNA polymerase II
apoptotic process
negative regulation of DNA-templated transcription
protein transport
lipid metabolic process
intracellular protein transport
proteasome-mediated ubiquitin-dependent protein catabolic process
positive regulation of canonical NF-kappaB signal transduction
regulation of apoptotic process
autophagy
positive regulation of transcription by RNA polymerase II
chromatin remodeling
cell division
negative regulation of apoptotic process
cell migration
DNA damage response
DNA repair
RNA splicing
mRNA processing
vesicle-mediated transport
-log10(PValue)
3
2
Count
14.00
28.00
56.00
0 2 4 6 8 10
Gene ratio (%)
D
F_HBO1 SA vs F_HBO1
343 genes upregulated
292 genes downregulated
translation
cytoplasmic translation
proton transmembrane transport
proton motive force-driven mitochondrial ATP synthesis
negative regulation of apoptotic process
mitochondrial translation
rRNA processing
mitochondrial electron transport, NADH to ubiquinone
aerobic respiration
regulation of apoptotic process
regulation of transcription by RNA polymerase II
chromatin remodeling
regulation of DNA-templated transcription
positive regulation of DNA-templated transcription
DNA damage response
negative regulation of DNA-templated transcription
protein transport
cell migration
DNA repair
regulation of cell cycle
-log10(PValue)
40
20
Count
9.00
22.05
54.00
0 5 10 15 20
Gene ratio (%)

E

Gene Category	Comparison	Expression Change	Total Gene Number	Promoter-Bound Gene Number	Proportion of Promoter Occupancy
F_HBO1 & F_HBO1 SA Co-Bound Genes	WM-3835 vs DMSO	Upregulated	229	157	68.6%
		Downregulated	402	202	50.2%
	F_HBO1 SA vs F_HBO1	Upregulated	104	55	52.9%
		Downregulated	215	104	48.4%
F_HBO1 SA-specific Bound Genes	WM-3835 vs DMSO	Upregulated	775	717	92.5%
		Downregulated	662	596	90.0%
	F_HBO1 SA vs F_HBO1	Upregulated	343	298	86.9%
		Downregulated	292	272	93.2%

F

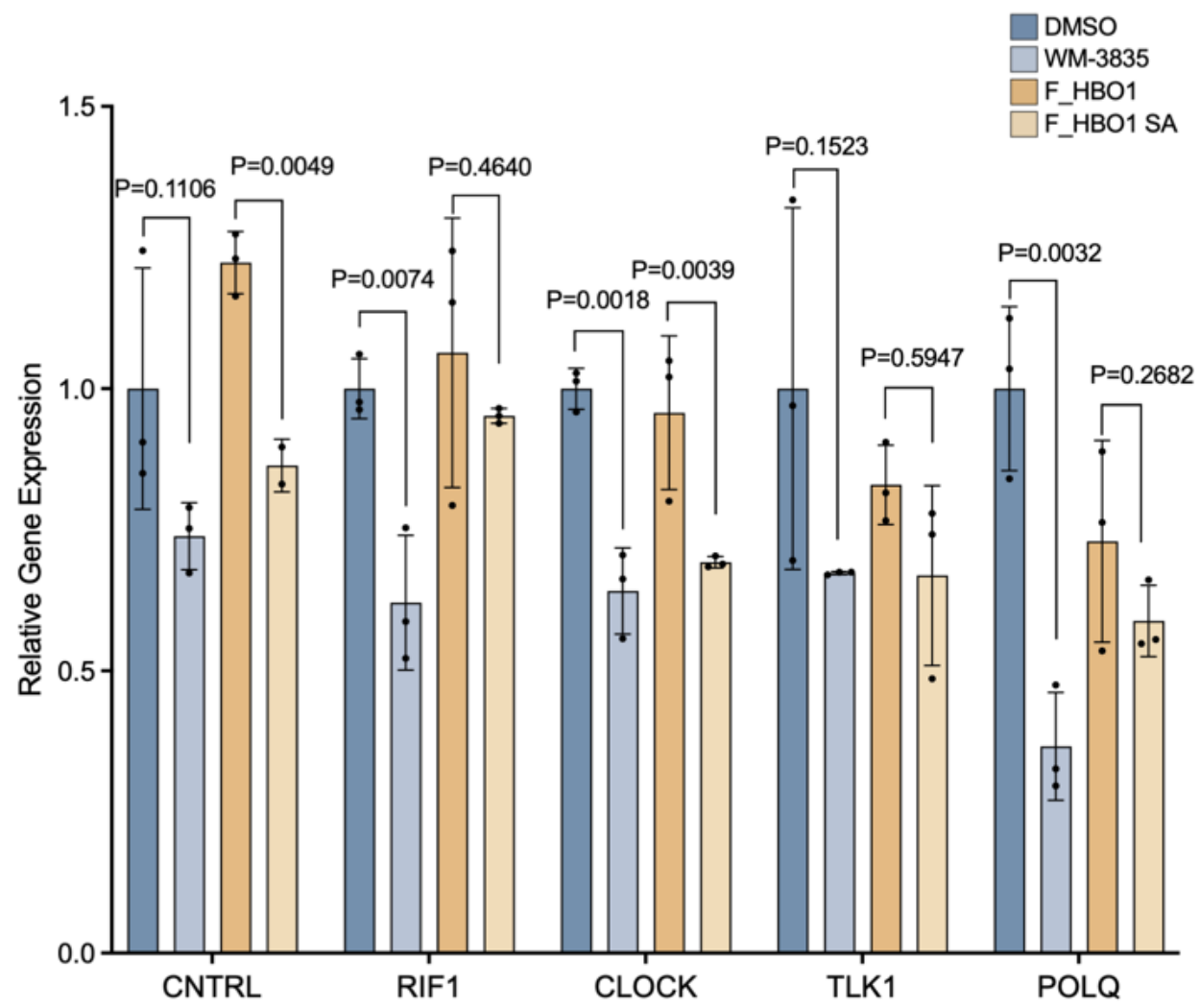

Fig. S3 Analysis of differentially expressed genes regulated by HBO1 or HBO1 SA.

(A) GO enrichment analysis of 631 co-bound genes (HBO1 and HBO1 SA) showing differential expression following WM-3835 treatment. **(B)** GO enrichment analysis of 319 co-bound genes (HBO1 and HBO1 SA) exhibiting differential expression upon Ser50/53 mutation. **(C)** GO enrichment analysis of 1,437 genes specifically bound by HBO1 SA and differentially expressed after WM-3835 treatment. **(D)** GO enrichment analysis of 635 genes specifically bound by HBO1 SA and differentially expressed upon Ser50/53 mutation. **(E)** Promoter occupancy analysis of HBO1 and HBO1 SA

bound genes with differential expression. **(F)** qPCR validation of RNA-seq results in OMM2.3 cells. Genes *CNTRL* and *RIF1* were commonly bound by F_HBO1 and F_HBO1 SA, while *CLOCK*, *TLK1* and *POLQ* were specifically bound by F_HBO1 SA. Gene expression was normalized to β-Actin (n=3 technical replicates; mean ± SD; one-way ANOVA followed by post-hoc multiple comparisons).

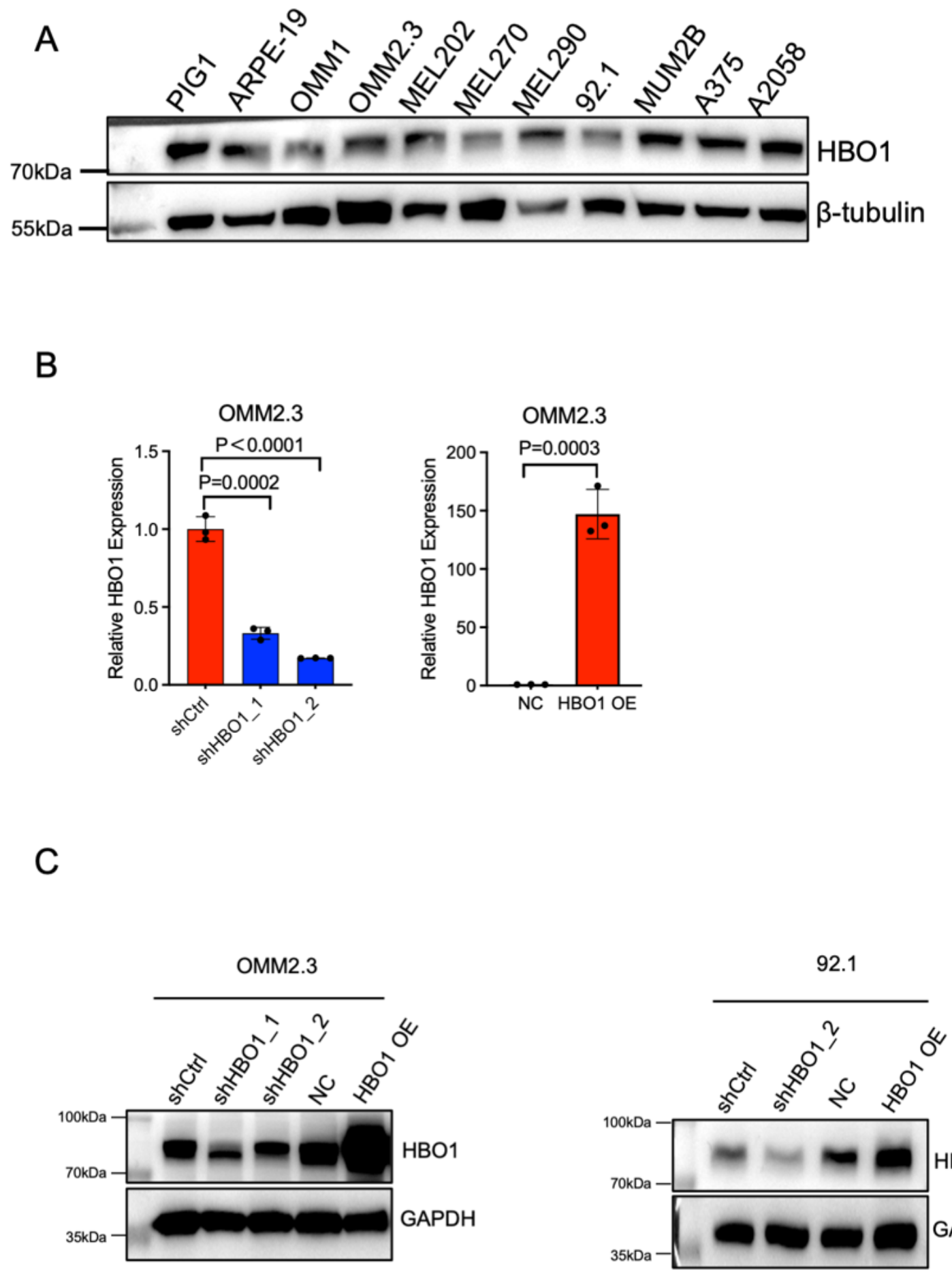

Fig. S4 HBO1 expression in indicated cell lines.

(A) Immunoblot analysis of endogenous HBO1 protein levels across multiple cell lines. **(B)** RT-qPCR validation of HBO1 knockdown and overexpression efficiency in OMM2.3 cells. **(C)** WB confirmation of HBO1 knockdown and overexpression efficiency in OMM2.3 and 92.1 cell lines.

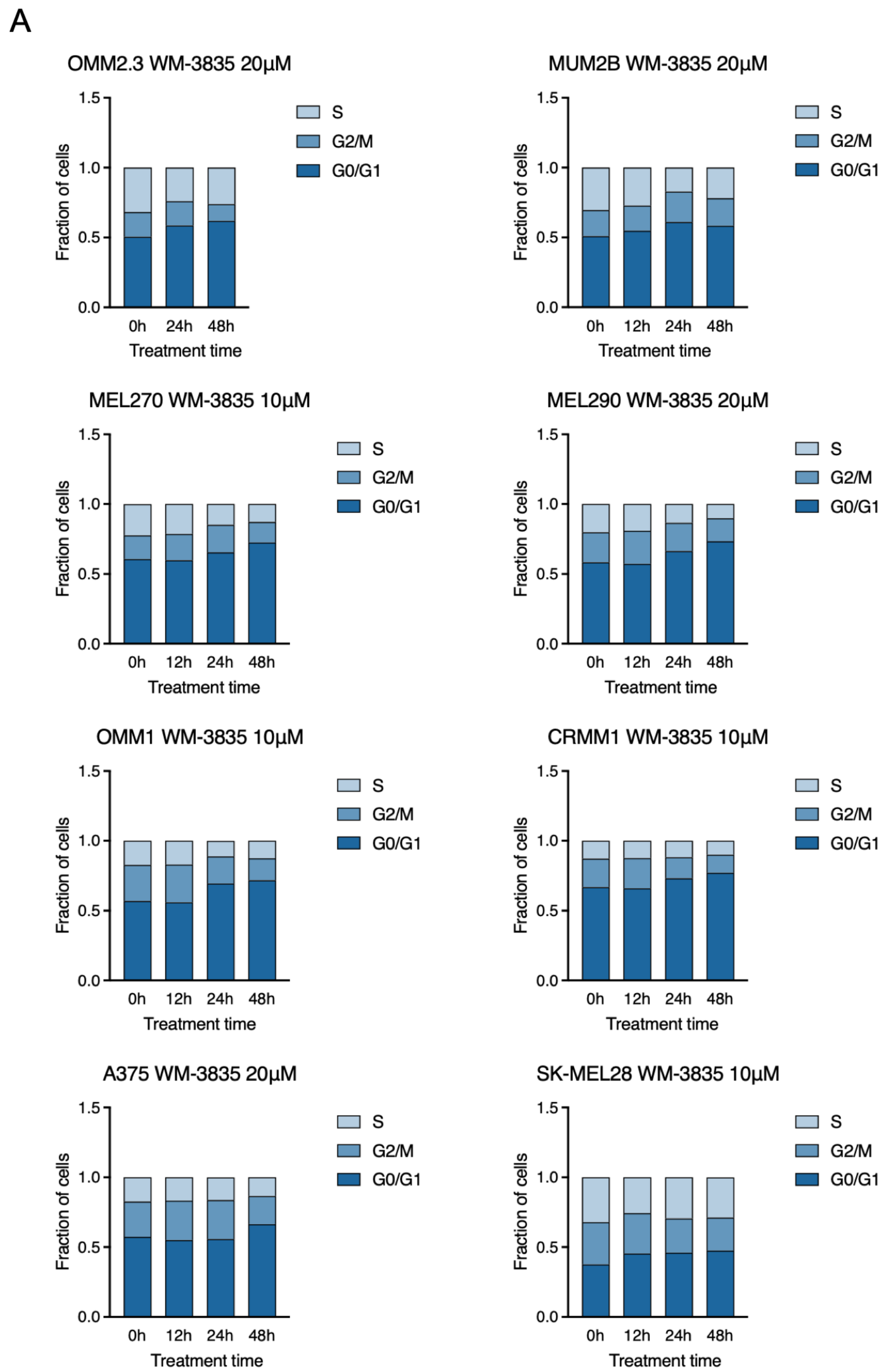

Fig. S5 Cell cycle analysis of melanoma cell lines treated with WM-3835.

(A) Flow cytometry analysis of cell cycle distribution in melanoma cell lines treated with WM-3835 for 0, 12, 24, and 48 hours. Cells were stained with propidium iodide (PI) and analyzed for DNA content to determine the fraction of cells in G0/G1, S, and G2/M phases.

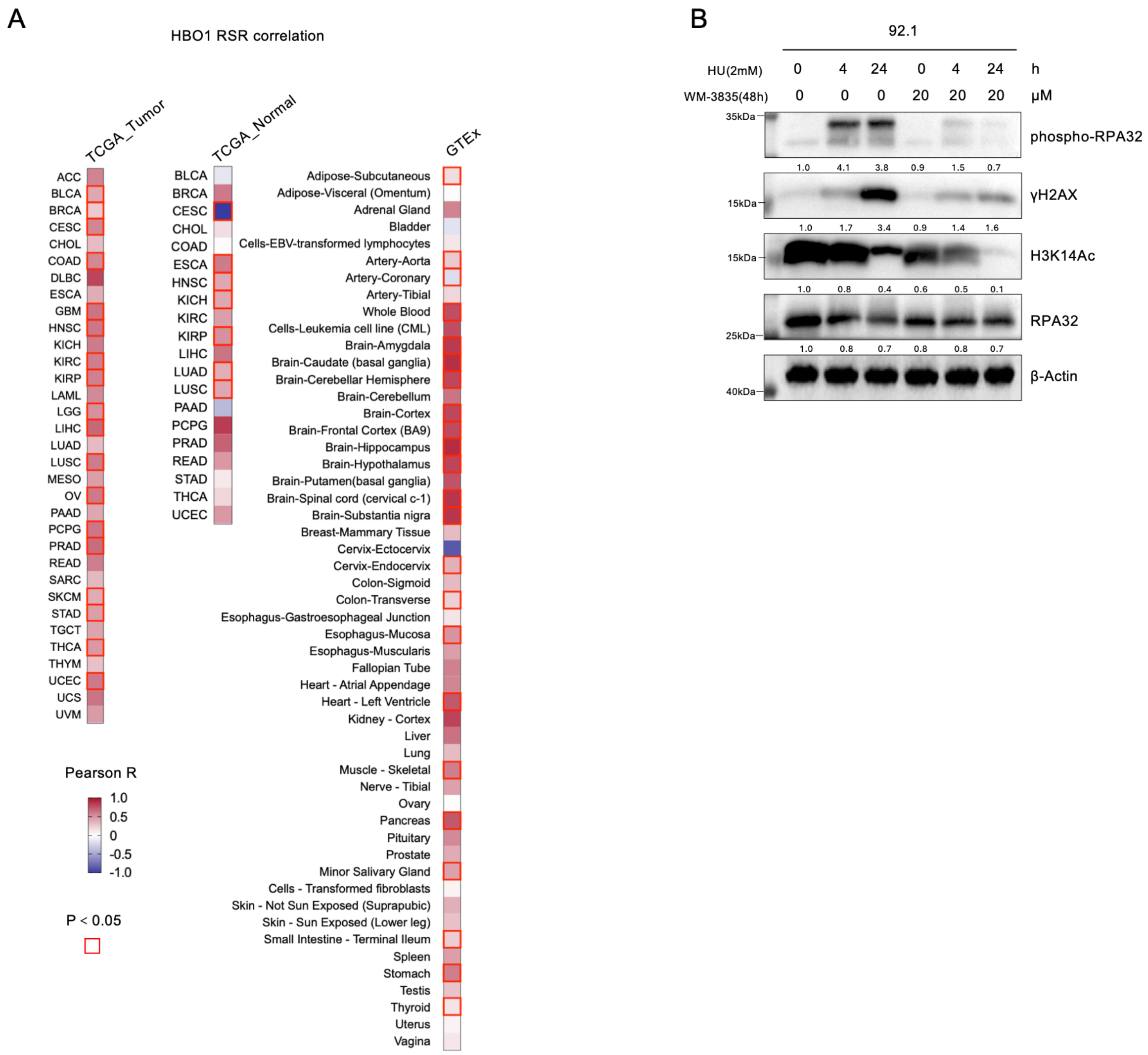

Fig. S6 HBO1 regulates replication stress response.

(A) Correlation analysis between HBO1 expression and RSR signature scores across TCGA tumor (n=33), TCGA normal (n=23), and GTEx (n=52) cohorts. Pearson correlation coefficient (R) and corresponding P values are shown (red box highlights significant correlations with P < 0.05). **(B)** Western blot analysis of key replication stress response pathway proteins in 92.1 cells treated with HU (2mM for 0, 4, and 24 hours) in combination with WM-3835 (0 or 20μM for 48 hours).

A

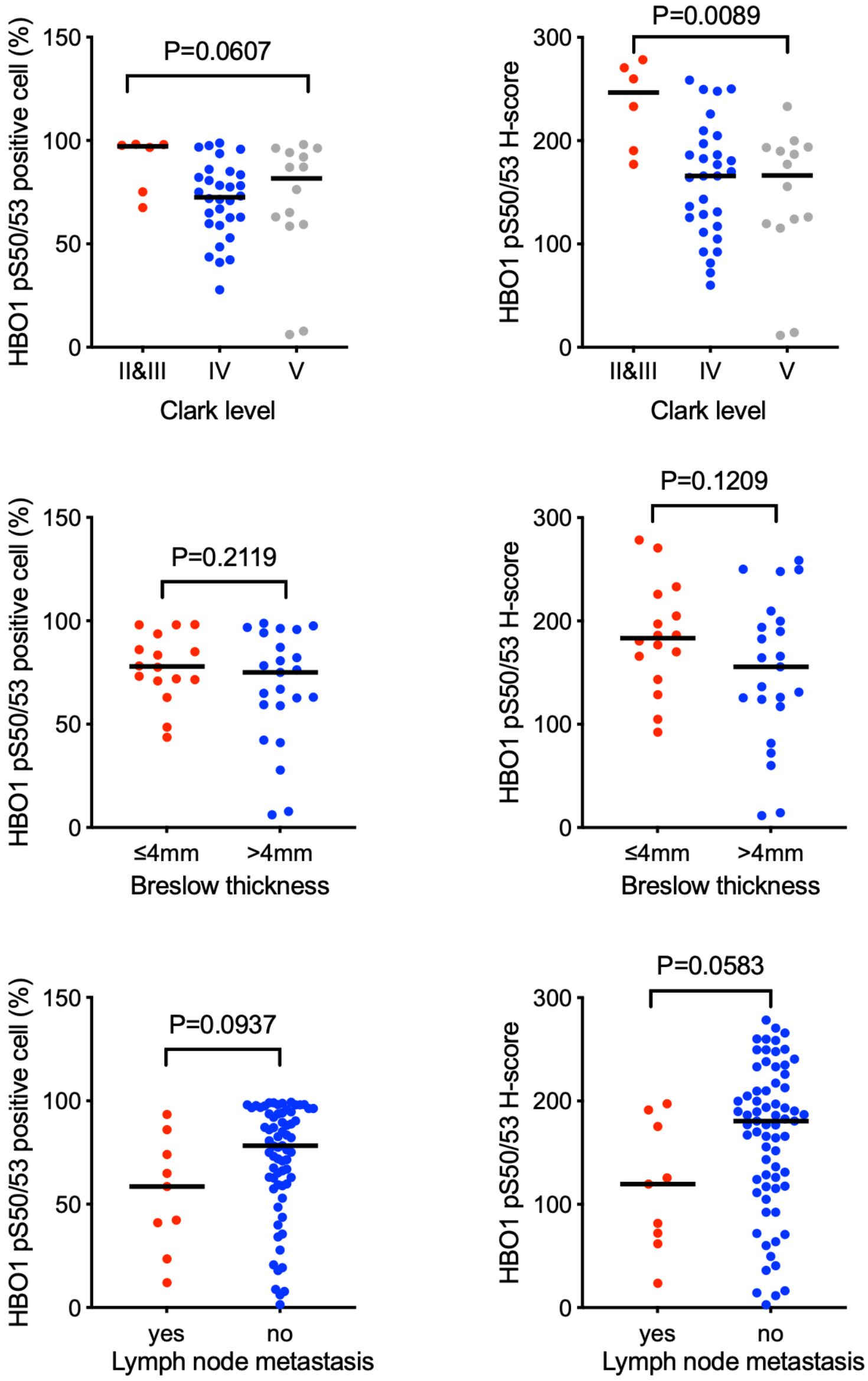
HBO1 pS50/53 positive cell (%)
P=0.0607
II&III
IV
V
Clark level
HBO1 pS50/53 H-score
P=0.0089
II&III
IV
V
Clark level
HBO1 pS50/53 positive cell (%)
P=0.2119
≤4mm
>4mm
Breslow thickness
HBO1 pS50/53 H-score
P=0.1209
≤4mm
>4mm
Breslow thickness
HBO1 pS50/53 positive cell (%)
P=0.0937
yes
no
Lymph node metastasis
HBO1 pS50/53 H-score
P=0.0583
yes
no
Lymph node metastasis

B

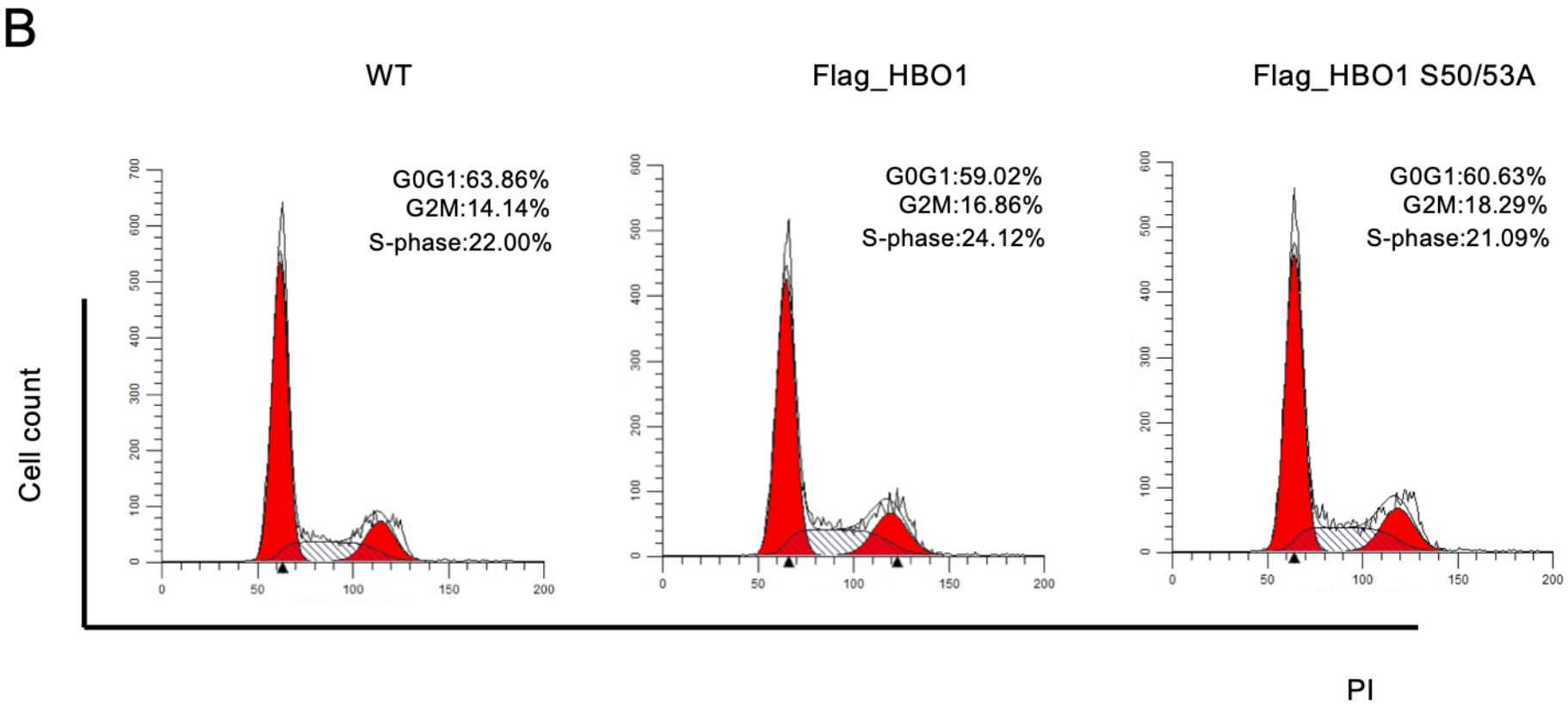
WT
Flag_HBO1
Flag_HBO1 S50/53A
G0G1:63.86%
G2M:14.14%
S-phase:22.00%
G0G1:59.02%
G2M:16.86%
S-phase:24.12%
G0G1:60.63%
G2M:18.29%
S-phase:21.09%
Cell count
PI

Fig. S7 Clinical correlation and functional analysis of HBO1 phosphorylation in melanoma.

(A) Quantitative analysis of HBO1 pS50/53-positive cells in melanoma specimens. Scatter plots show HBO1 pS50/53 positivity and H-scores stratified by: (i) Clark level (I-V), (ii) Breslow thickness (≤4 mm vs >4 mm), and (iii) lymph node metastasis status (yes/no). **(B)** Cell cycle profiling by flow cytometry in OMM2.3 cells. Comparative analysis of cell cycle distribution (G0/G1, S, G2/M phases) among WT, F_HBO1 and F_HBO1 SA overexpressed OMM2.3 cells.

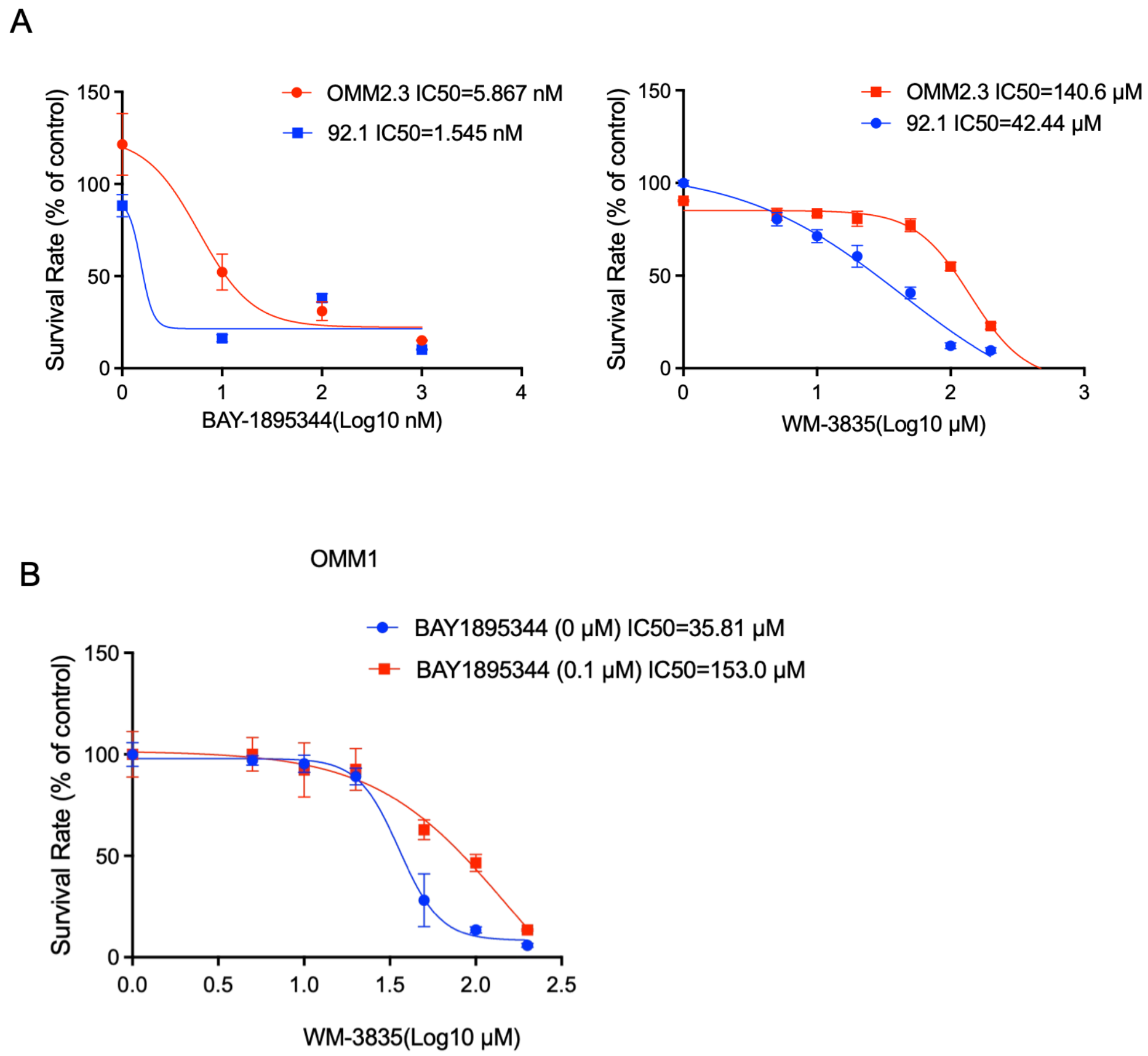

Fig. S8 Drug sensitivity analysis of BAY-1895344 and WM-3835.

(A) Left: Cell viability assessed by CCK-8 assay in OMM2.3 and 92.1 cells treated with BAY-1895344 monotherapy. Right: Cell viability assessed by CCK-8 assay in OMM2.3 and 92.1 cells treated with WM-3835 monotherapy. **(B)** IC50 values of WM-3835 in OMM1 cells incubated with 0 μM or 0.1 μM BAY-1895344. Data are presented as mean ± SD from three independent biological replicates.
