# Supplementary material for: ATR-dependent phosphorylation of the histone acetyltransferase HBO1 suppresses chromatin binding and promotes replication stress responses
Source: J Biol Chem. 2026 Aug 10;302(10):113419. doi: 10.1016/j.jbc.2026.113419 (PMC13570308; doi:10.1016/j.jbc.2026.113419)
Supplement: Supplementary file 2 [file mmc3.pdf]

Table S2_HBO1-survival

TCGA Tumor	OS-HR	OS-p(HR)	-Log10 p value
ACC	2	9.20E-02	1.04
BLCA	1.2	0.15	0.82
BRCA	1.1	0.57	0.24
CESC	0.9	0.65	0.19
CHOL	1.2	0.67	0.17
COAD	0.69	0.14	0.85
DLBC	2.2	0.3	0.52
ESCA	1.2	0.37	0.43
GBM	0.79	0.19	0.72
HNSC	0.9	0.42	0.38
KICH	4.2	0.072	1.14
KIRC	0.54	0.00012	3.92
KIRP	1.4	0.24	0.62
LAML	1.1	0.72	0.14
LGG	0.72	7.30E-02	1.14
LIHC	1.2	0.22	0.66
LUAD	0.99	0.94	0.03
LUSC	0.95	0.68	0.17
MESO	0.85	0.52	0.28
OV	1.1	0.46	0.34
PAAD	1	0.88	0.06
PCPG	1.3	0.75	0.12
PRAD	1.3	0.67	0.17
READ	0.79	0.62	0.21
SARC	0.75	0.17	0.77
SKCM	1.1	0.35	0.46
STAD	1.2	0.35	0.46
TGCT	2.4	0.44	0.36
THCA	1.3	0.61	0.21
THYM	0.92	0.91	0.04
UCEC	0.86	0.67	0.17
UCS	0.94	0.86	0.07
UVM	2.9	0.029	1.54

Table S3_HBO1-RSR pearson correlation

TCGA Tumor	P value	R	TCGA Normal	P value	R
ACC	5.2e−08	0.57			
BLCA	0.00	0.41	BLCA	0.53000	-0.15
BRCA	0.00	0.26	BRCA	7.1e−13	0.61
CESC	0.00	0.56	CESC	0.03000	-1
CHOL	0.06	0.32	CHOL	0.68000	0.16
COAD	0.00	0.55	COAD	0.91000	0.019
DLBC	5.9e−12	0.81			
ESCA	4.1e−08	0.39	ESCA	0.02900	0.6
GBM	0.00	0.63			
HNSC	0.00	0.62	HNSC	0.00600	0.41
KICH	1.3e−07	0.60	KICH	0.04400	0.41
KIRC	0.00	0.59	KIRC	6.4e−05	0.45
KIRP	0.00	0.58	KIRP	0.00300	0.51
LAML	2.3e−14	0.54			
LGG	0.00	0.51			
LIHC	0.00	0.68	LIHC	8.8e−07	0.63
LUAD	2e−13	0.33	LUAD	0.00330	0.38
LUSC	0.00	0.60	LUSC	0.00280	0.41
MESO	1.3e−05	0.45			
OV	0.00	0.61			
PAAD	1.3e−05	0.41	PAAD	0.60000	-0.4
PCPG	0.00	0.62	PCPG	0.37000	0.84
PRAD	0.00	0.66	PRAD	5.5e−09	0.7
READ	6e−10	0.59	READ	0.16000	0.48
SARC	1.9e−08	0.34	SARC		
SKCM	0.00	0.40	SKCM		
STAD	0.00	0.45	STAD	0.52000	0.11
TGCT	1.9e−07	0.43			
THCA	0.00	0.48	THCA	0.13000	0.2
THYM	9e−04	0.30	THYM		
UCEC	0.00	0.61	UCEC	0.09600	0.48
UCS	2e−07	0.63			
UVM	1.3e−05	0.47			

GTEx	P value	R
Adipose-Subcutaneous	0.00250	0.17
Adipose-Visceral (Omentum)	0.80000	0.018
Adrenal Gland	1.9e−12	0.57
Bladder	0.66000	-0.17
Cells-EBV-transformed lymphocytes	0.22000	0.12
Artery-Aorta	0.00013	0.26
Artery-Coronary	0.03100	-0.2
Artery-Tibial	9e−04	0.2
Whole Blood	0.00000	0.78
Cells-Leukemia cell line (CML)	2.4e−15	0.78
Brain-Amygdala	0.00000	0.85
Brain-Anterior cingulate cortex (BA24)		
Brain-Caudate (basal ganglia)	0.00000	0.89
Brain-Cerebellar Hemisphere	0.00000	0.8
Brain-Cerebellum	1.3e−14	0.63
Brain-Cortex	0.00000	0.8
Brain-Frontal Cortex (BA9)	0.00000	0.79
Brain-Hippocampus	0.00000	0.9
Brain-Hypothalamus	0.00000	0.81
Brain-Nucleus accumbens (basal ganglia)		
Brain-Putamen (basal ganglia)	4.4e−16	0.76
Brain-Spinal cord (cervical c-1)	0.00000	0.87
Brain-Substantia nigra	0.00000	0.87
Breast-Mammary Tissue	1.1e−05	0.32
Cervix-Ectocervix	0.02300	-0.87
Cervix-Endocervix	0.63000	−0.37
Colon-Sigmoid	6.6e−05	0.33
Colon-Transverse	0.00240	0.23
Esophagus-Gastroesophageal Junction	0.12000	0.13
Esophagus-Mucosa	0.00000	0.5
Esophagus-Muscularis	6.7e−14	0.45
Fallopian Tube	0.30000	−0.58
Heart - Atrial Appendage	8.9e−16	0.56
Heart - Left Ventricle	0.00000	0.74
Kidney - Cortex	9.7e−08	0.82
Liver	4.1e−14	0.64
Lung	3.3e−08	0.32
Muscle - Skeletal	0.00000	0.59
Nerve - Tibial	2.6e−14	0.44
Ovary	0.96000	−0.0048
Pancreas	0.00000	0.74
Pituitary	1.8e−09	0.54
Prostate	3.3e−05	0.4
Minor Salivary Gland	0.00071	0.44
Cells - Transformed fibroblasts	0.31000	0.063
Skin - Not Sun Exposed (Suprapubic)	2.1e−09	0.38
Skin - Sun Exposed (Lower leg)	2.4e−08	0.3
Small Intestine - Terminal Ileum	0.02100	0.24
Spleen	2.7e−06	0.45
Stomach	0.00000	0.59
Testis	2e−04	0.29
Thyroid	0.02800	0.13
Uterus	0.61000	0.059
Vagina	0.29000	0.12

Table S4 Primers used for cloning.

Name	Sequence (5' – 3')
HBO1 D1 Fwd	ATAGAAGACACCGGCGGCCACGCGTATGCCGCGAAGGAAGAGGAAT
HBO1 D1 Rev	ATCGAATTCCTGCAGCCCGGGGGATCCCTGCTTATCCCGGCTCTGTGC
HBO1 D2 Fwd	ATAGAAGACACCGGCGGCCACGCGTATGGAGAGCATTGCCAAGGACATGT
HBO1 D2 Rev	CGTCGTCATCCTTGTAATCCTCGAGAGTGCCCTTGGGAGGGGTCC
HBO1 D3 Fwd	ATAGAAGACACCGGCGGCCACGCGTATGATAGAAGAAAGGATGCTGTCTC ACAGGC

Table S5 Primers used for RT-PCR.

Gene	Forward sequence	Reverse sequence
HBO1	ATTCTGGACTGAGCAAAGAACAG	GTCATACTCGCTTGTCAGGTTTT
ATR	GGCCAAAGGCAGTTGTATTGA	GTGAGTACCCCAAAAATAGCAGG
β-Actin	CCTGTACGCCAACACAGTGC	ATACTCCTGCTTGCTGATCC

Table S6_Tissue Array information

Cat No.	ZL-MEL361
Diagram	

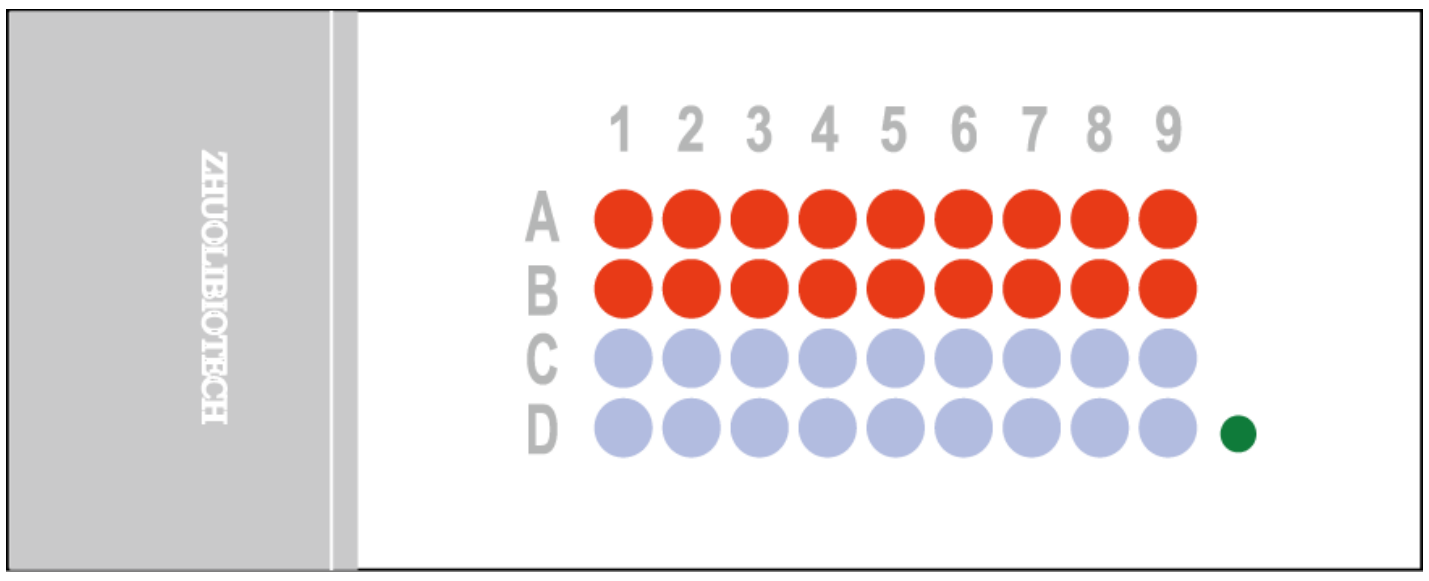

position	gender	Path.No	Pathological Diagnosis	
A01	Female	13-02564	left foot, malignant melanoma	
A02	Male	15-11262	small intestine, malignant melanoma metastasis	
A03	Female	15-15082	foot, malignant melanoma	
A04	Male	16-07641	rectal, malignant melanoma	
A05	Male	16-08920	inner eyelid, malignant melanoma	
A06	Female	16-14316	rectal, malignant melanoma	
A07	Female	13-15766	foot, malignant melanoma	
A08	Female	14-26231	orbital, malignant melanoma	
A09	Female	11-12436	foot, malignant melanoma	
B01	Female	13-02564	left foot, malignant melanoma	
B02	Male	15-11262	small intestine, malignant melanoma metastasis	
B03	Female	15-15082	foot, malignant melanoma	
B04	Male	16-07641	rectal, malignant melanoma	
B05	Male	16-08920	inner eyelid, malignant melanoma	
B06	Female	16-14316	rectal, malignant melanoma	
B07	Female	13-15766	foot, malignant melanoma	
B08	Female	14-26231	orbital, malignant melanoma	
B09	Female	11-12436	foot, malignant melanoma	
C01	Female	16-00466A	normal skin	
C02	Female	17-06580D	normal skin	
C03	Female	17-00524A	normal skin	
C04	Female	17-04120A	normal skin	
C05	Female	14-22927A	normal skin	
C06	Female	17-03301E	normal skin	
C07	Female	17-04121A	normal skin	
C08	Female	14-20552A	normal skin	
C09	Female	14-20553A	normal skin	
D01	Female	16-00466A	normal skin	
D02	Female	17-06580D	normal skin	
D03	Female	17-00524A	normal skin	
D04	Female	17-04120A	normal skin	
D05	Female	14-22927A	normal skin	
D06	Female	17-03301E	normal skin	
D07	Female	17-04121A	normal skin	
D08	Female	14-20552A	normal skin	
D09	Female	14-20553A	normal skin	
D10	Positioning point			

ZL-MEL963

Diagram

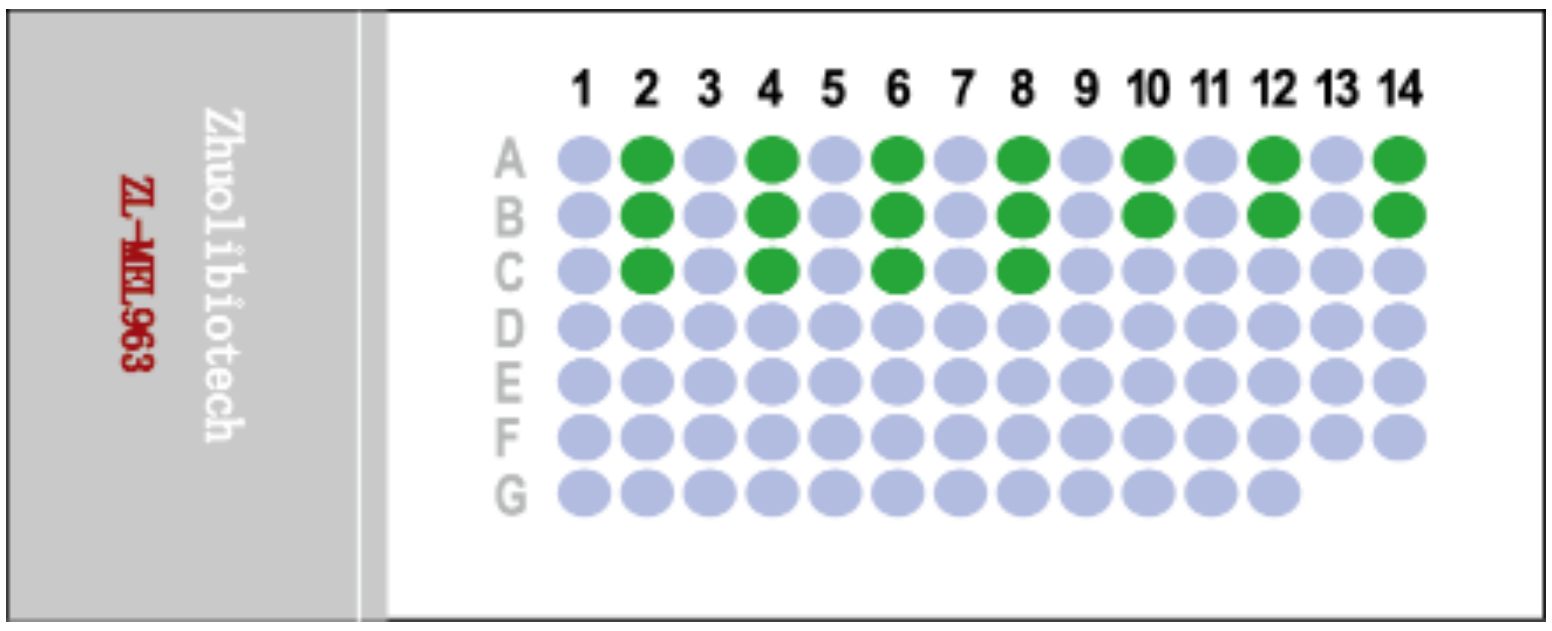

Position	Type	Gender	Age	Path. No.	Location	Clark level	Pathological Diagnosis
A01	Carcinoma	Male	70	202200516	Plantar surface of foot	4	Malignant melanoma
A02	Adjacent normal						
A03	Carcinoma	Female	63	202220146	Plantar surface of foot	4	Malignant melanoma
A04	Adjacent normal						
A05	Carcinoma	Male	69	202220852	Gingiva	5	Malignant melanoma
A06	Adjacent normal						
A07	Carcinoma	Male	67	202235163	Plantar surface of foot	5	Malignant melanoma
A08	Adjacent normal						
A09	Carcinoma	Male	67	202245794	Plantar surface of foot	4	Malignant melanoma
A10	Adjacent normal						
A11	Carcinoma	Female	40	202252791	Plantar surface of foot	4	Malignant melanoma
A12	Adjacent normal						
A13	Carcinoma	Male	79	202257426	Rectum		Malignant melanoma
A14	Adjacent normal						
B01	Carcinoma	Female	75	202259606	Rectum		Malignant melanoma
B02	Adjacent normal						
B03	Carcinoma	Female	83	202261141	Plantar surface of foot	4	Malignant melanoma
B04	Adjacent normal						
B05	Carcinoma	Female	69	202267688	Plantar surface of foot	4	Malignant melanoma
B06	Adjacent normal						
B07	Carcinoma	Male	67	202268939	Plantar surface of foot	4	Malignant melanoma
B08	Adjacent normal						
B09	Carcinoma	Male	60	202310325	Plantar surface of foot	5	Malignant melanoma
B10	Adjacent normal						
B11	Carcinoma	Female	54	202317497	Vulva	4	Malignant melanoma
B12	Adjacent normal						
B13	Carcinoma	Male	71	202323640	skin	5	Malignant melanoma
B14	Adjacent normal						
C01	Carcinoma	Male	62	202365557	Plantar surface of foot	4	Malignant melanoma
C02	Adjacent normal						
C03	Carcinoma	Female	59	202366279	Plantar surface of foot	4	Malignant melanoma
C04	Adjacent normal						
C05	Carcinoma	Male	66	202374172	Plantar surface of foot	4	Malignant melanoma
C06	Adjacent normal						
C07	Carcinoma	Female	69	202376286	skin	4	Malignant melanoma
C08	Adjacent normal						
C09	Carcinoma	Female	75	201706809	skin	5	Malignant melanoma
C10							
C11	Carcinoma	Male	61	201711448	skin	5	Malignant melanoma
C12							
C13	Carcinoma	Male	81	201711576	Plantar surface of foot		Malignant melanoma
C14							
D01	Carcinoma	Male	48	201724506	Plantar surface of foot	2	Malignant melanoma
D02							
D03	Carcinoma	Female	79	201742171	Rectum		Malignant melanoma
D04							
D05	Carcinoma	Female	55	201747725	Axilla		Malignant melanoma
D06							

D07<br>D08	Carcinoma	Male	58	201750529	Plantar surface of foot		Malignant melanoma
D09<br>D10	Carcinoma	Female	76	201755904	Inguinal region		Malignant melanoma
D11<br>D12	Carcinoma	Male	70	202147547	skin	5	Malignant melanoma
D13<br>D14	Carcinoma	Female	32	202156172	Plantar surface of foot	3	Malignant melanoma
E01<br>E02	Carcinoma	Male	69	202160001	skin	4	Malignant melanoma
E03<br>E04	Carcinoma	Male	63	202174043	Plantar surface of foot	4	Malignant melanoma
E05<br>E06	Carcinoma	Female	56	202175607	skin	4	Malignant melanoma
E07<br>E08	Carcinoma	Female	71	202176404	skin	4	Malignant melanoma
E09<br>E10	Carcinoma	Female	67	202181549	Plantar surface of foot		Malignant melanoma
E11<br>E12	Carcinoma	Male	71	202182921	skin	3	Malignant melanoma
E13<br>E14	Carcinoma	Female	71	202184327	skin	4	Malignant melanoma
F01<br>F02	Carcinoma	Female	74	202206443	Rectum		Malignant melanoma
F03<br>F04	Carcinoma	Male	68	202208588	skin		Malignant melanoma
F05<br>F06	Carcinoma	Female	77	202212301	Plantar surface of foot	4	Malignant melanoma
F07<br>F08	Carcinoma	Female	69	202243578	Plantar surface of foot	5	Malignant melanoma
F09<br>F10	Carcinoma	Female	71	202245883	Plantar surface of foot	4	Malignant melanoma
F11<br>F12	Carcinoma	Male	68	202262725	Axilla		Malignant melanoma
F13<br>F14	Carcinoma	Female	63	202278326	Rectum		Malignant melanoma
G01<br>G02	Carcinoma	Female	71	202280362	Gingiva		Malignant melanoma
G03<br>G04	Carcinoma	Male	61	202280617	skin	5	Malignant melanoma
G05<br>G06	Carcinoma	Male	62	202309504	skin	4	Malignant melanoma
G07<br>G08	Carcinoma	Male	68	202345585	Nasal cavity		Malignant melanoma
G09<br>G10	Carcinoma	Male	76	202367556	Nasal cavity		Malignant melanoma
G11<br>G12	Carcinoma	Male	51	202378208	Plantar surface of foot	4	Malignant melanoma

Table S7 Replication stress response signature gene set.

KRAS	CCND2	NPM1	MYC	CCND1	MDM2
STAT5A	HRAS	CCNE1	CCND3	NRAS	DEK
CCNE2	CDC6	CDCA5	AURKA	MYBL2	E2F1
