# Supplementary material for: ATR-dependent phosphorylation of the histone acetyltransferase HBO1 suppresses chromatin binding and promotes replication stress responses
Source: J Biol Chem. 2026 Aug 10;302(10):113419. doi: 10.1016/j.jbc.2026.113419 (PMC13570308; doi:10.1016/j.jbc.2026.113419)
Supplement: Supplementary file 3 [file mmc4.pdf]

Uncropped original western blot images

Fig.1D

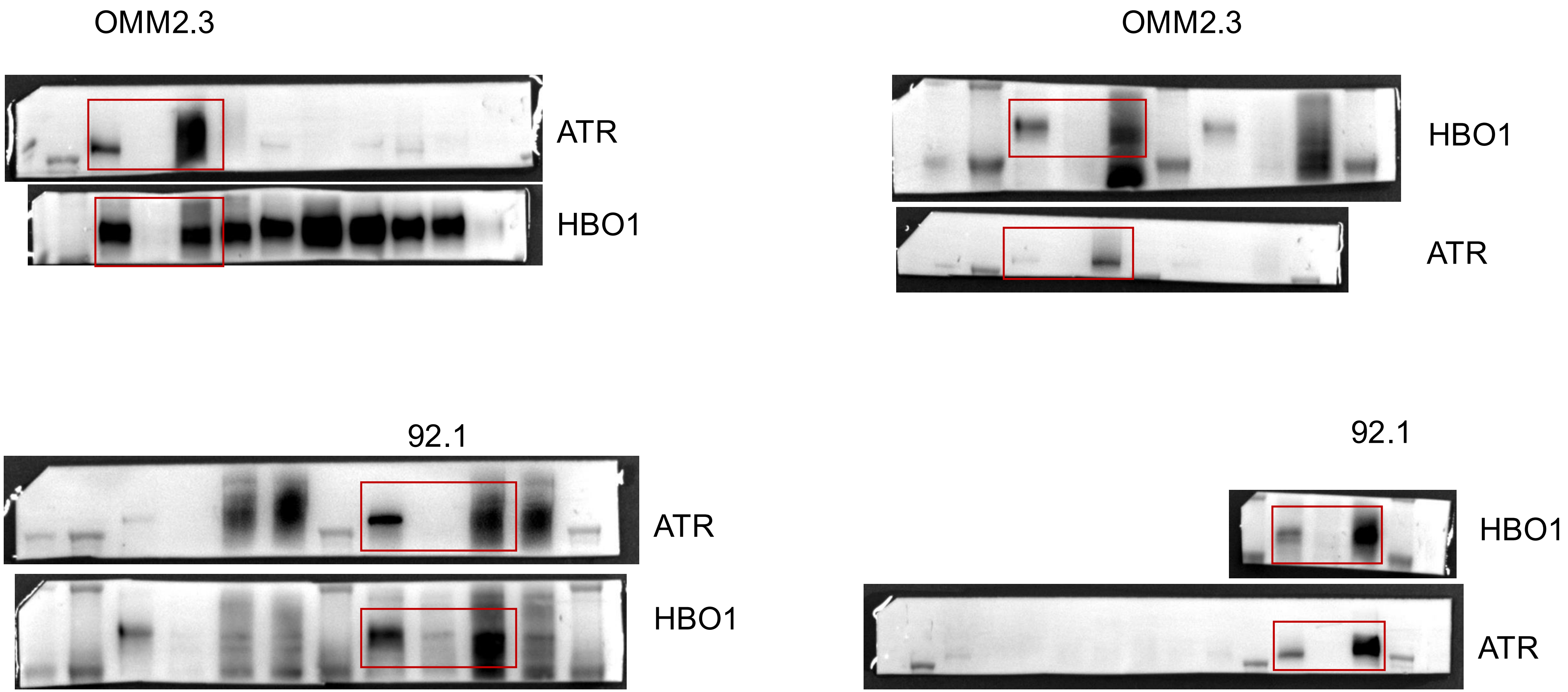

Fig.1E

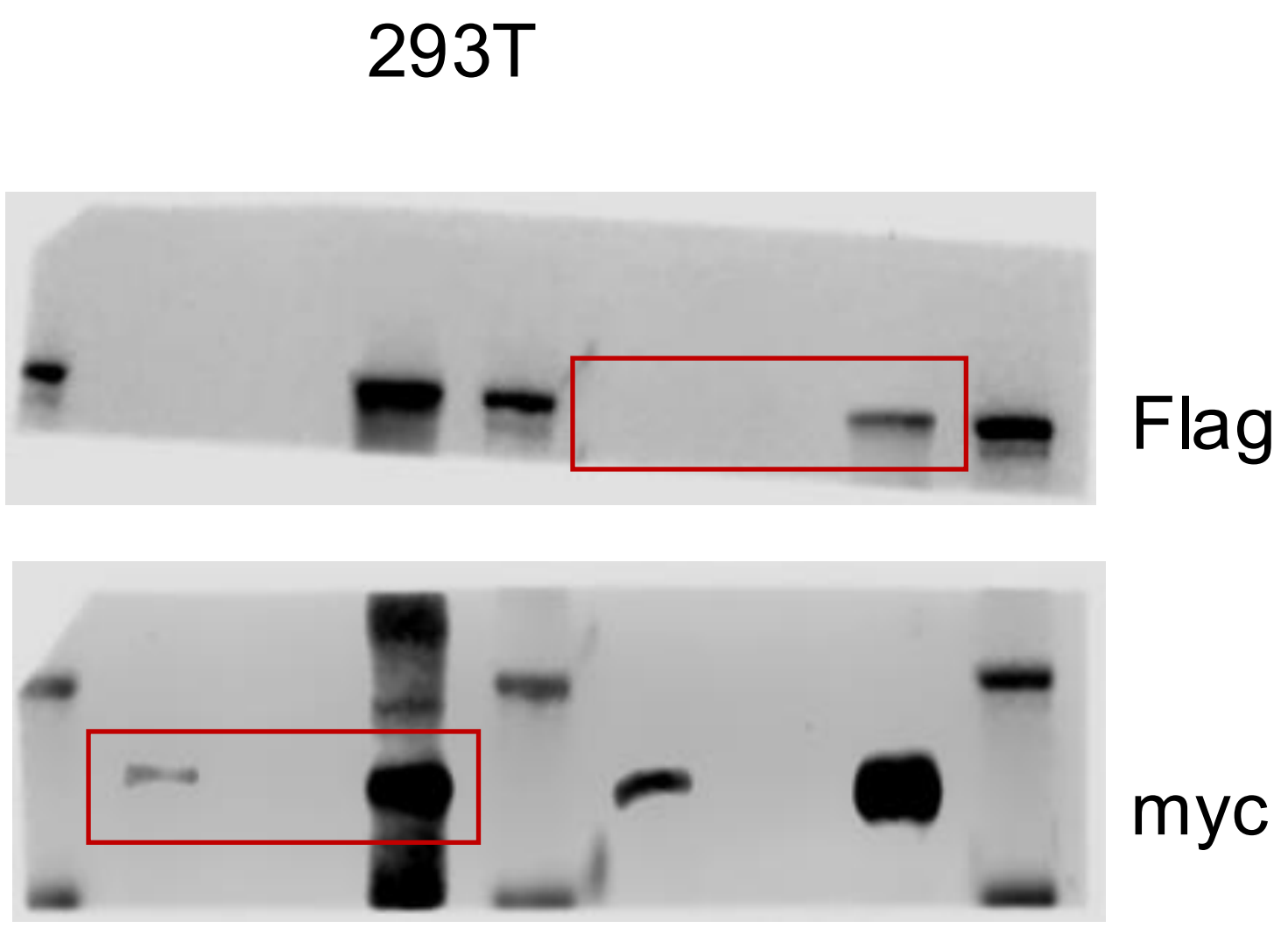

Fig.1F

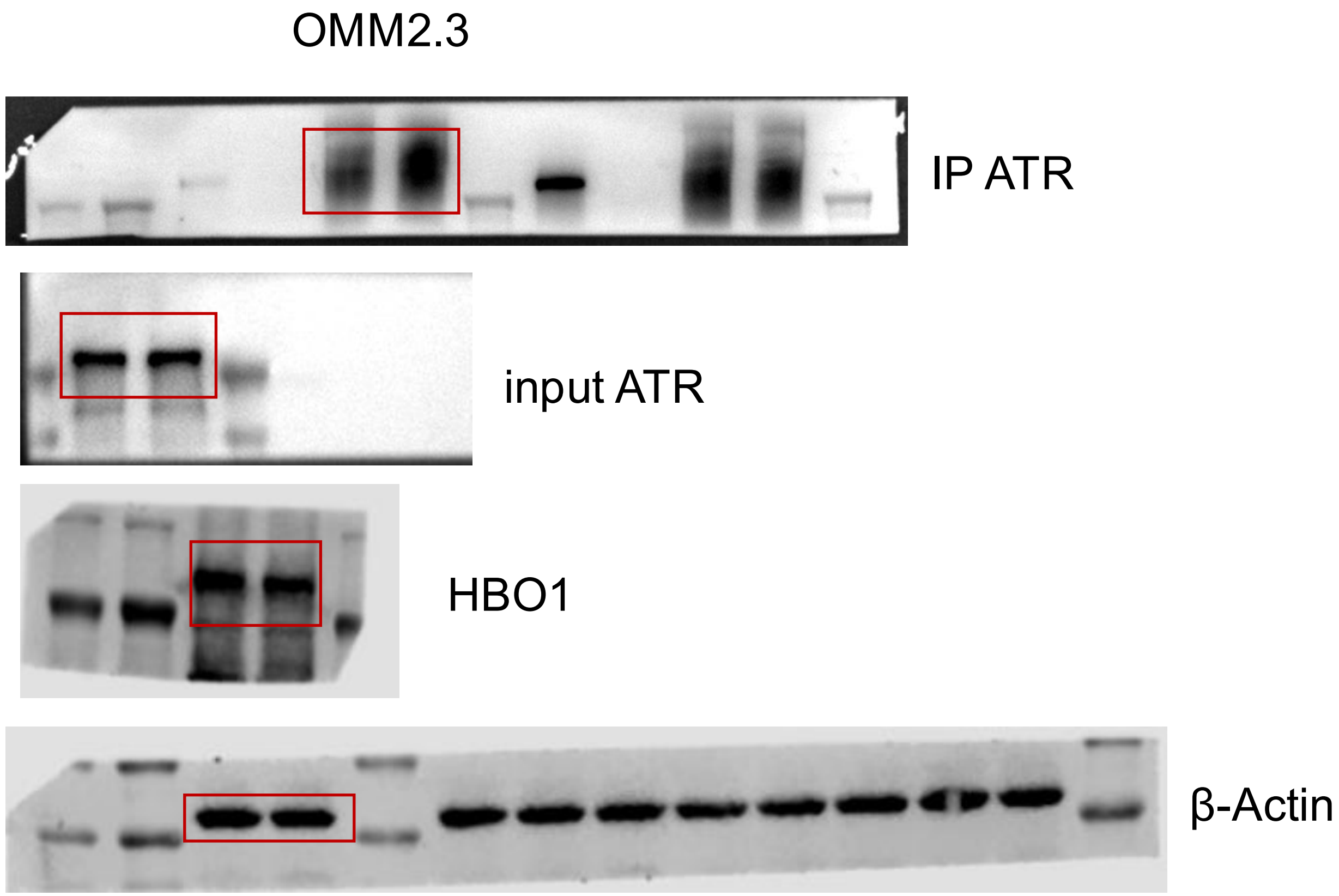

Fig.1F

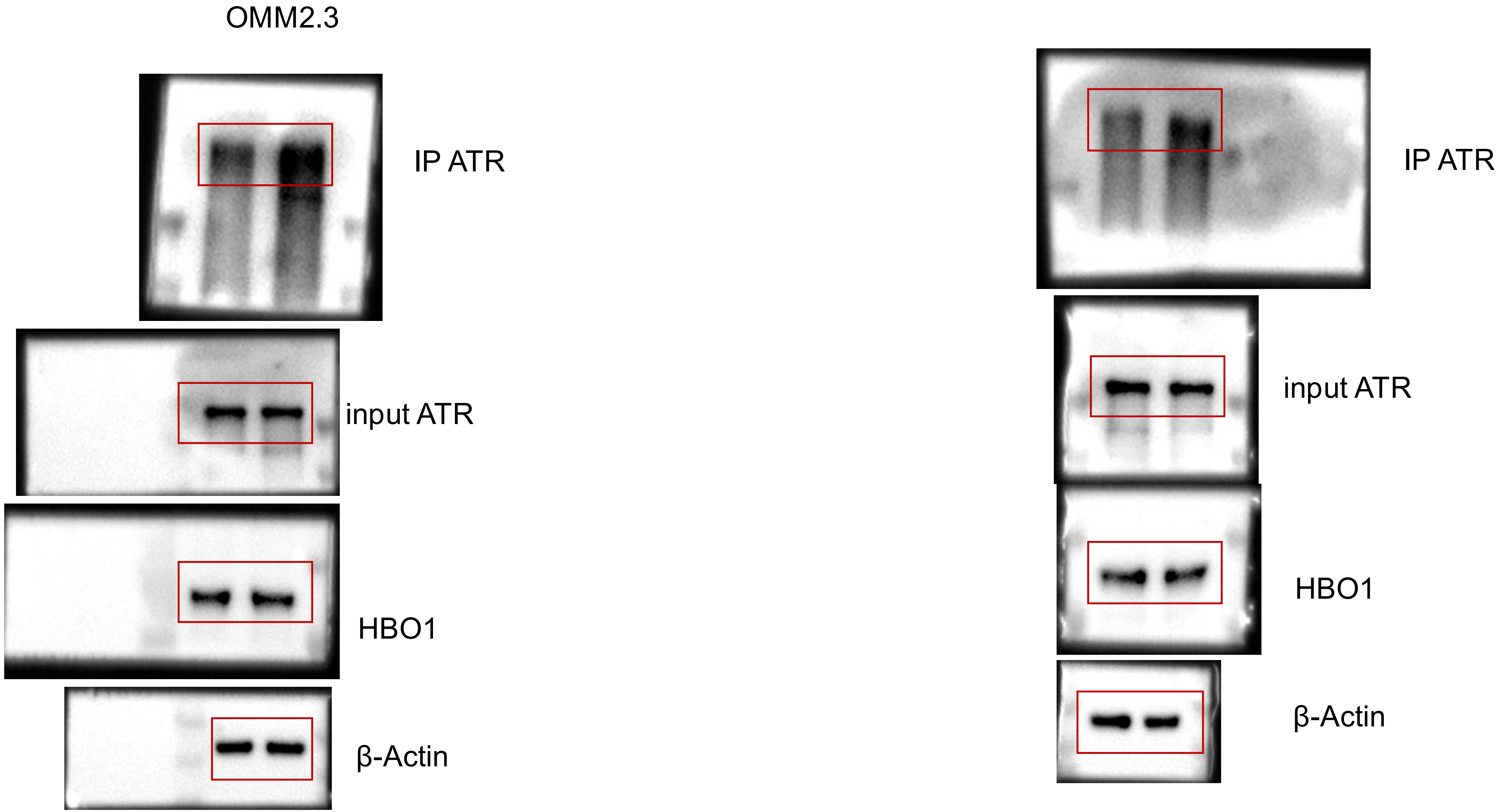
OMM2.3
IP ATR
input ATR
HBO1
β-Actin
OMM2.3
IP ATR
input ATR
HBO1
β-Actin

Fig.2B

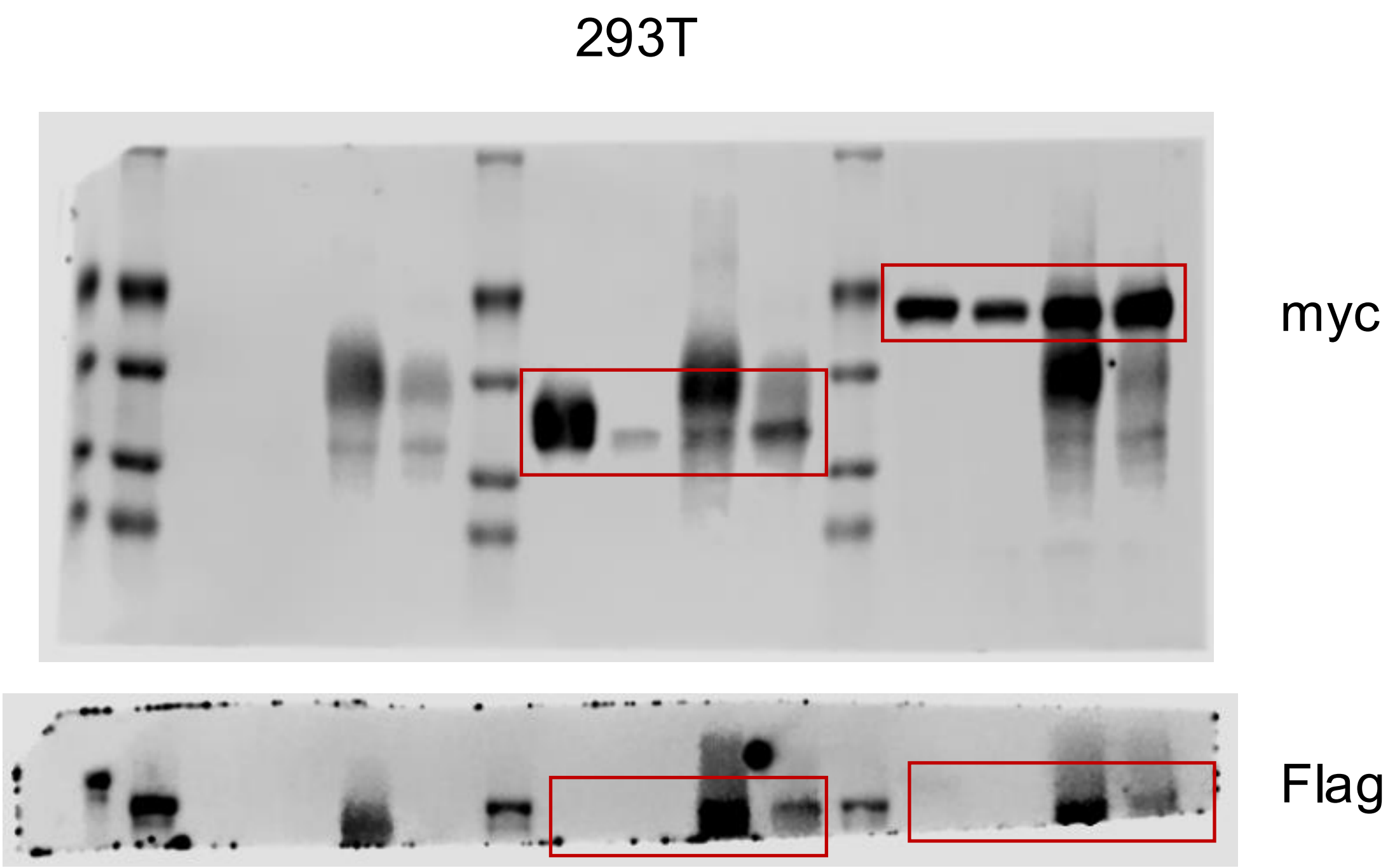

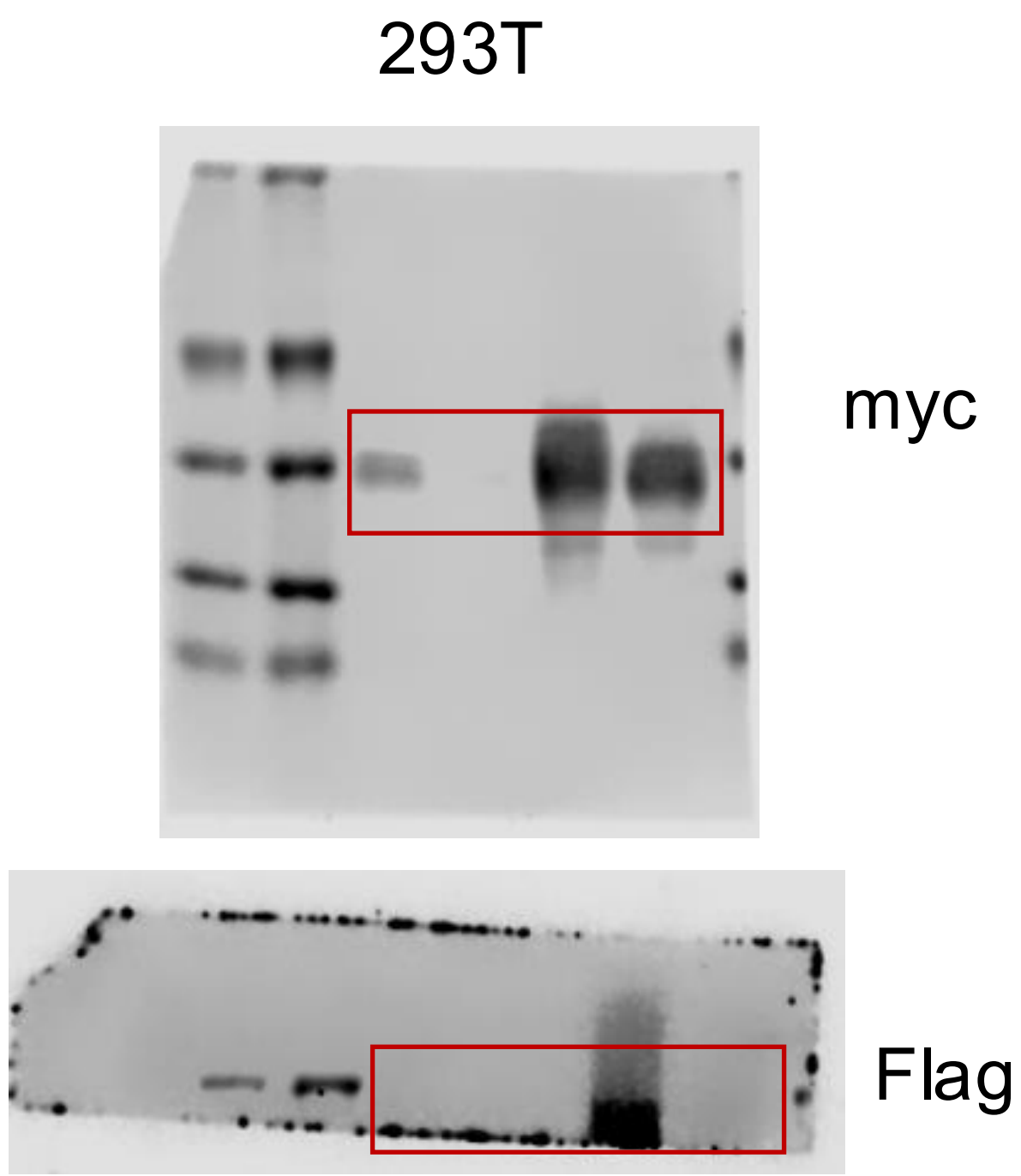

Fig.2C

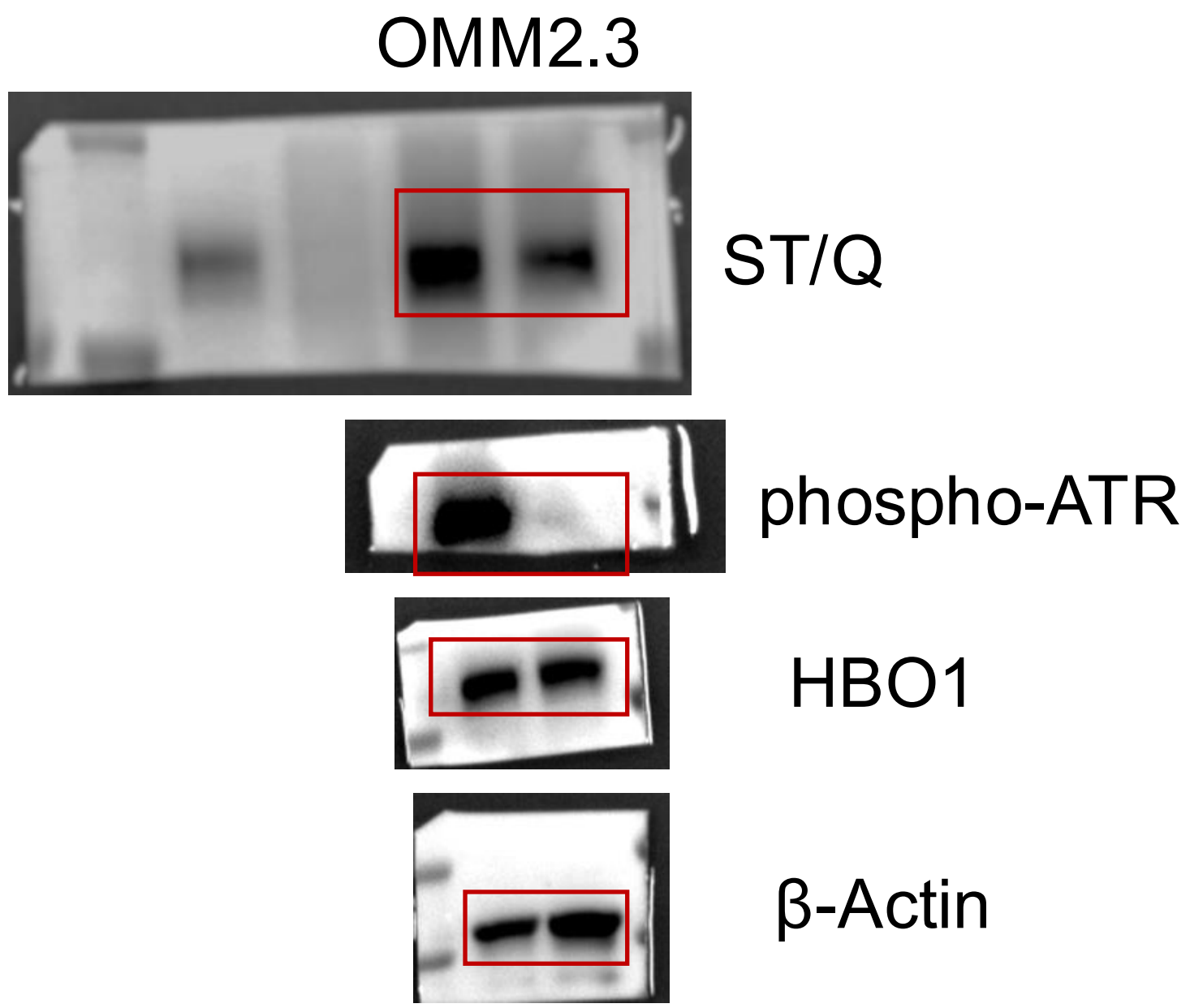

Fig.2D

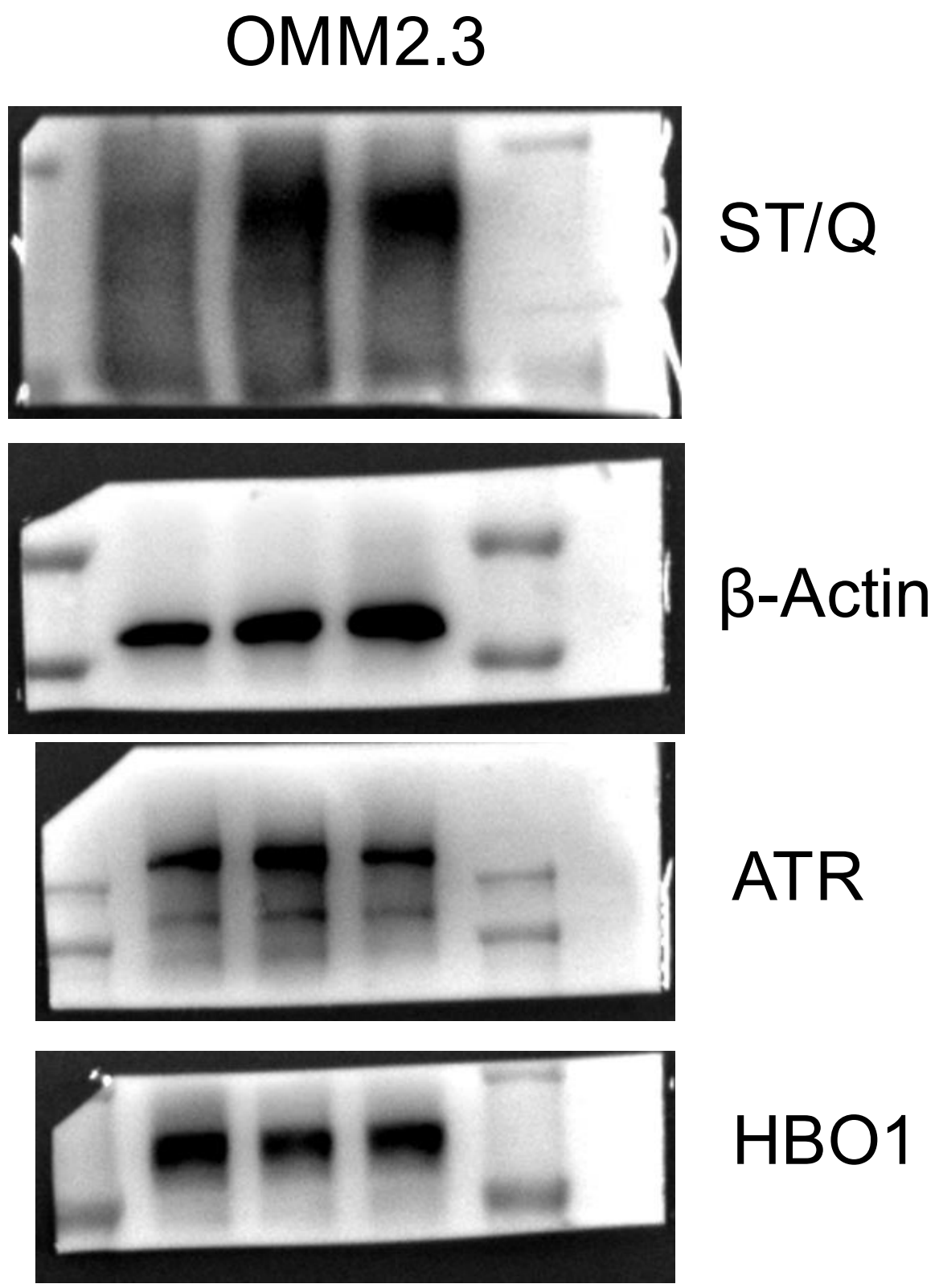

Fig.2F

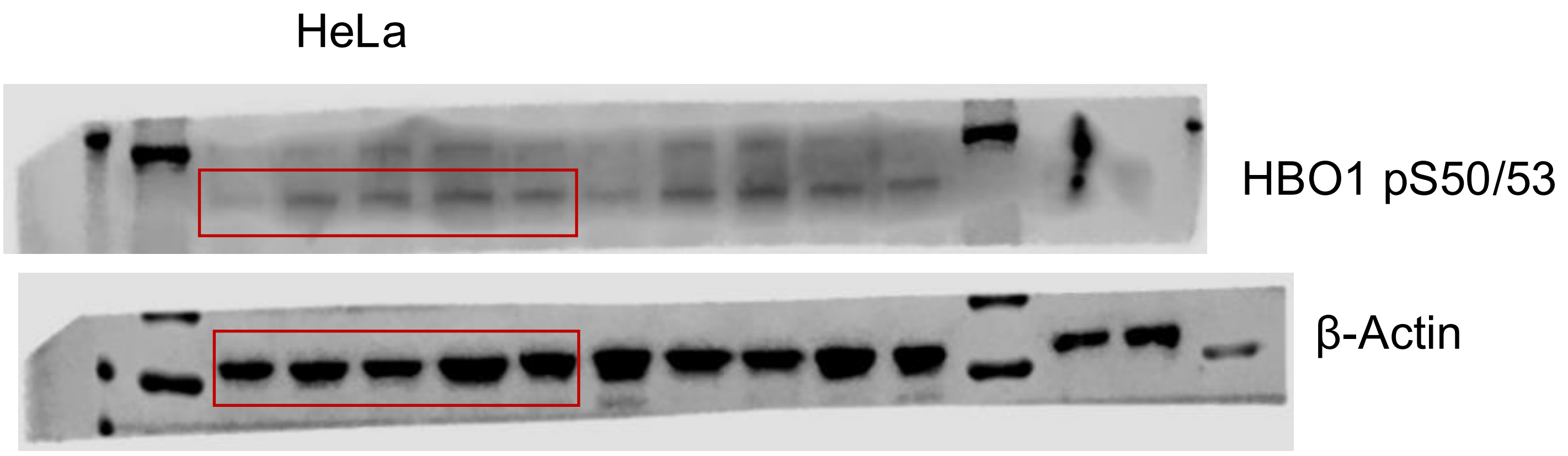

Fig.2H

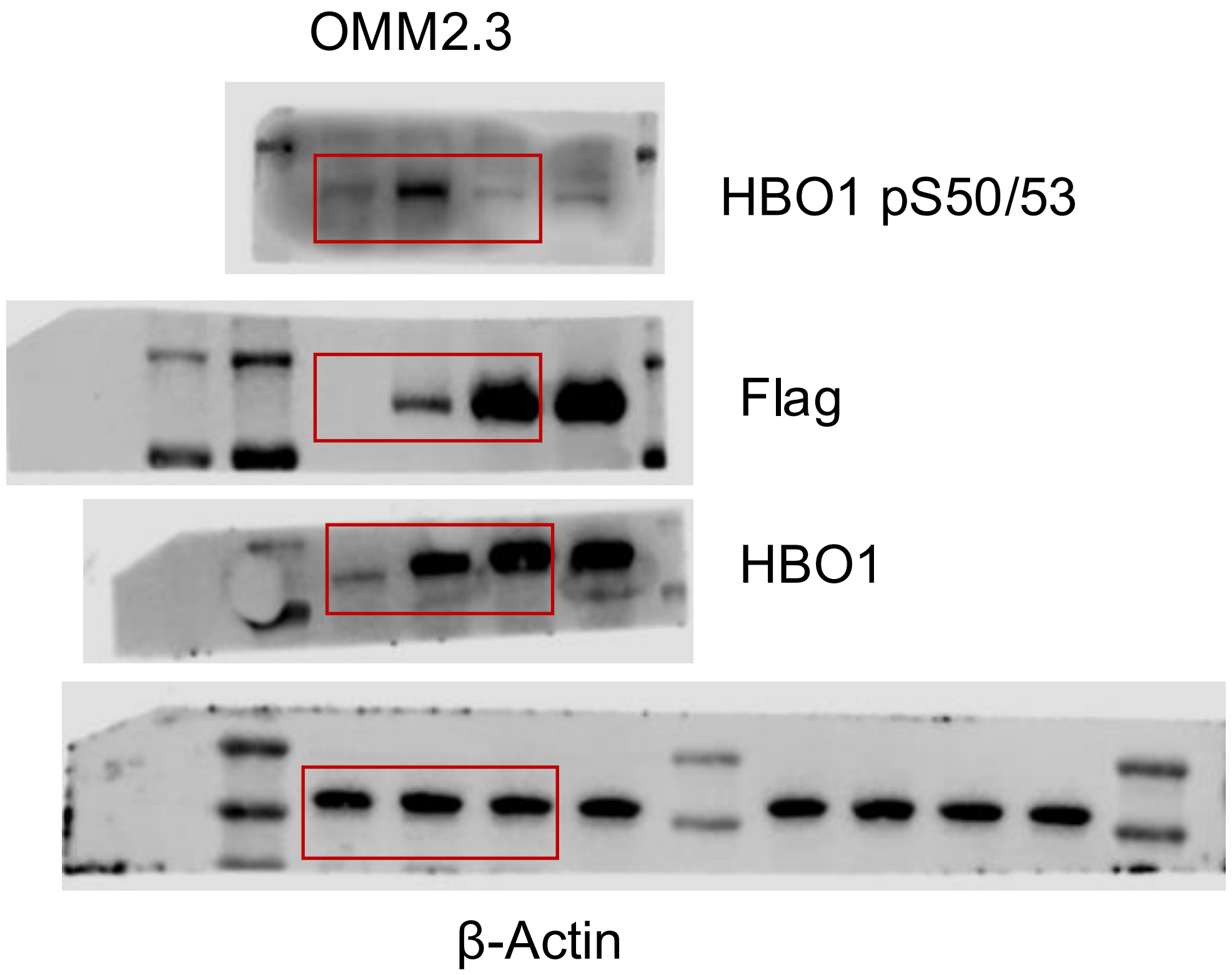

Fig.4F

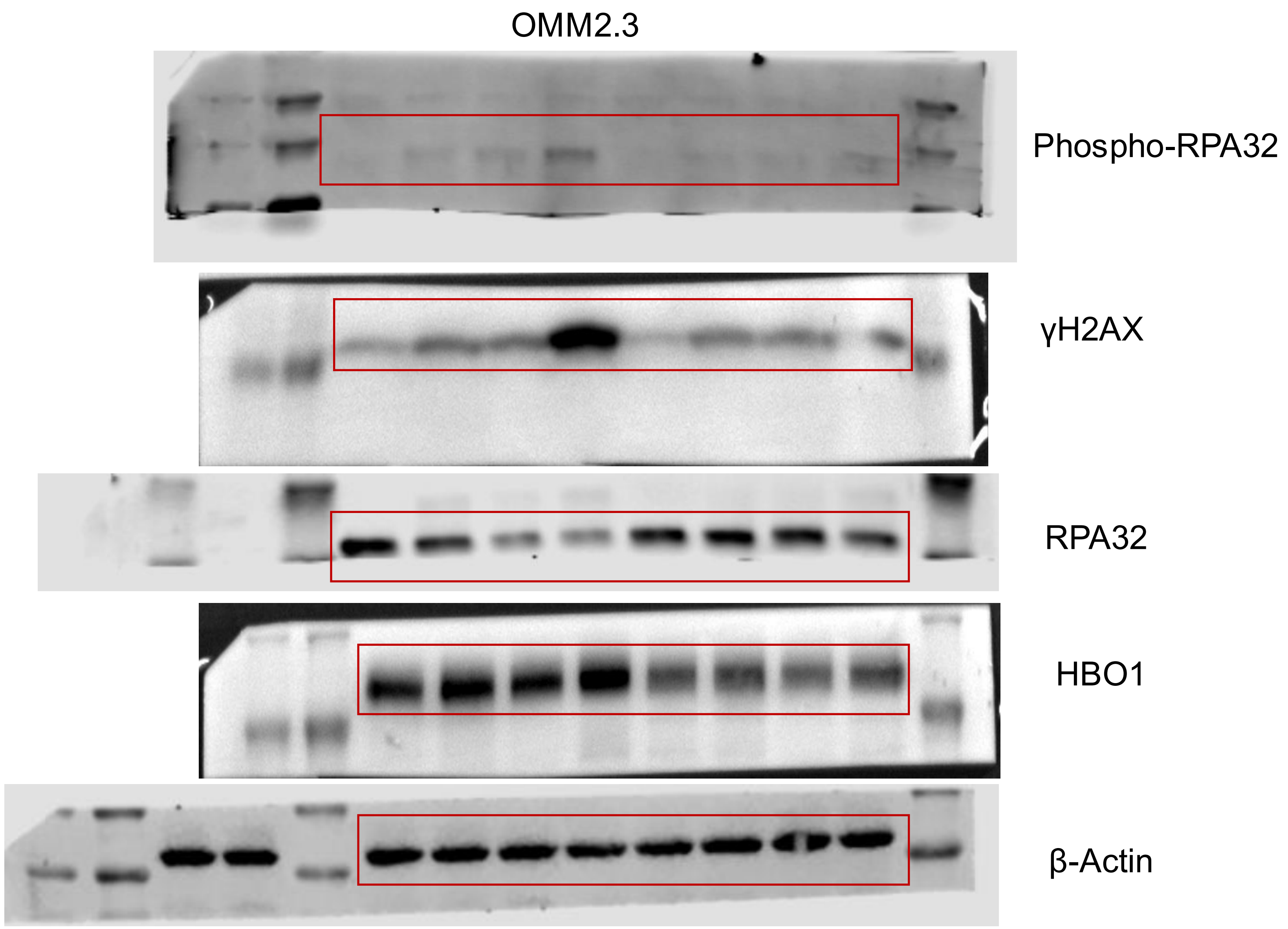

Fig.4G

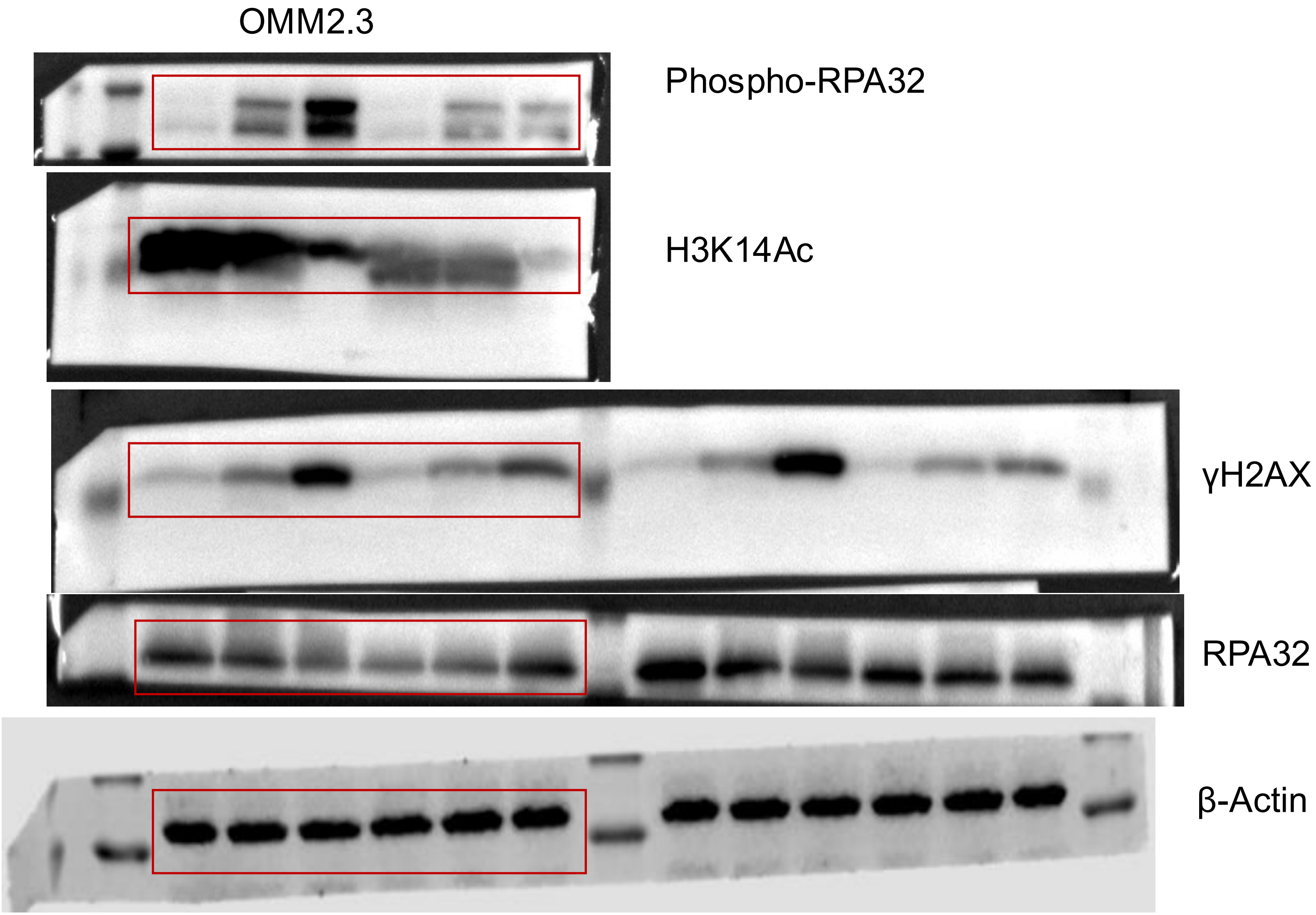

Fig.5D

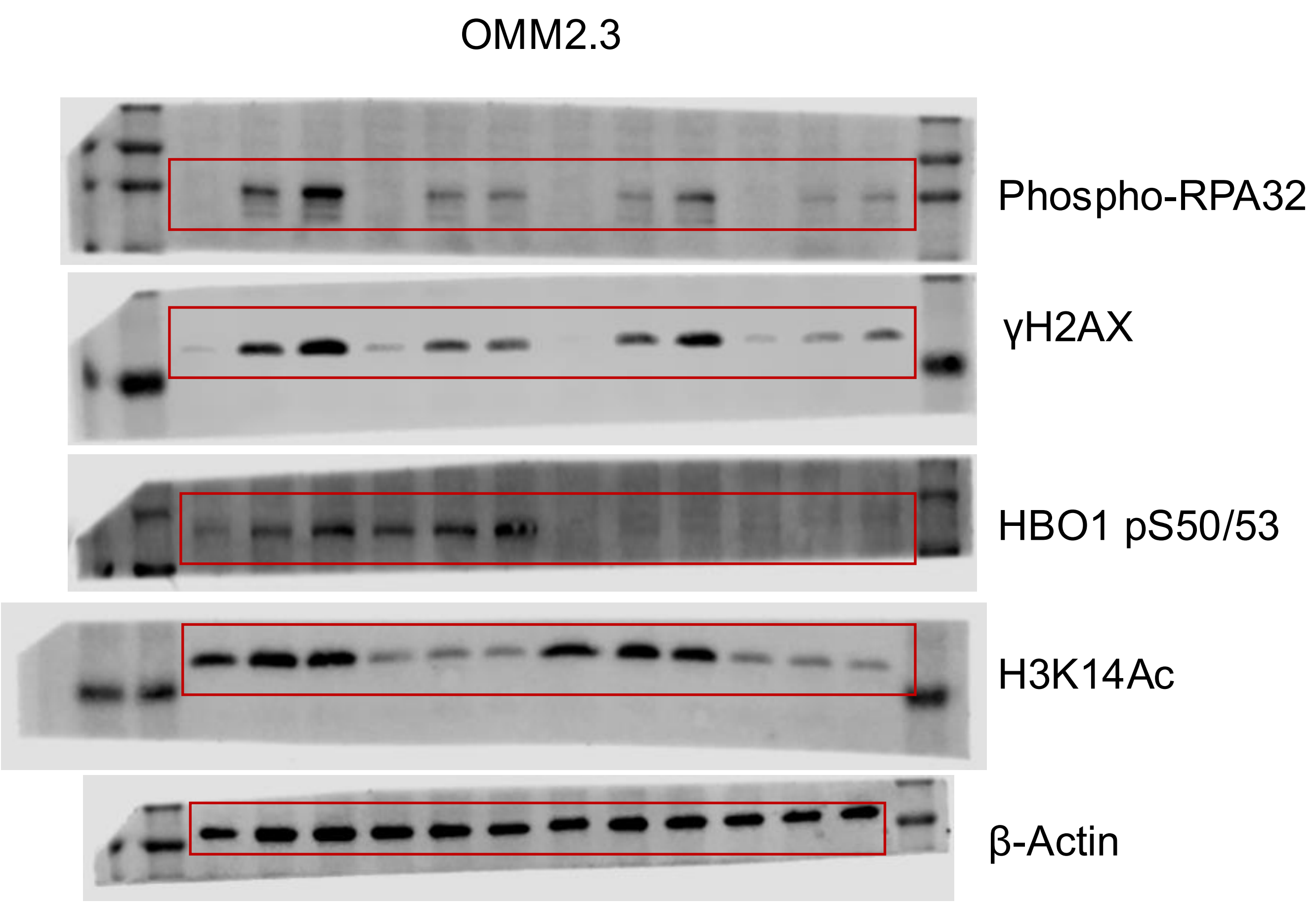

Fig.6D

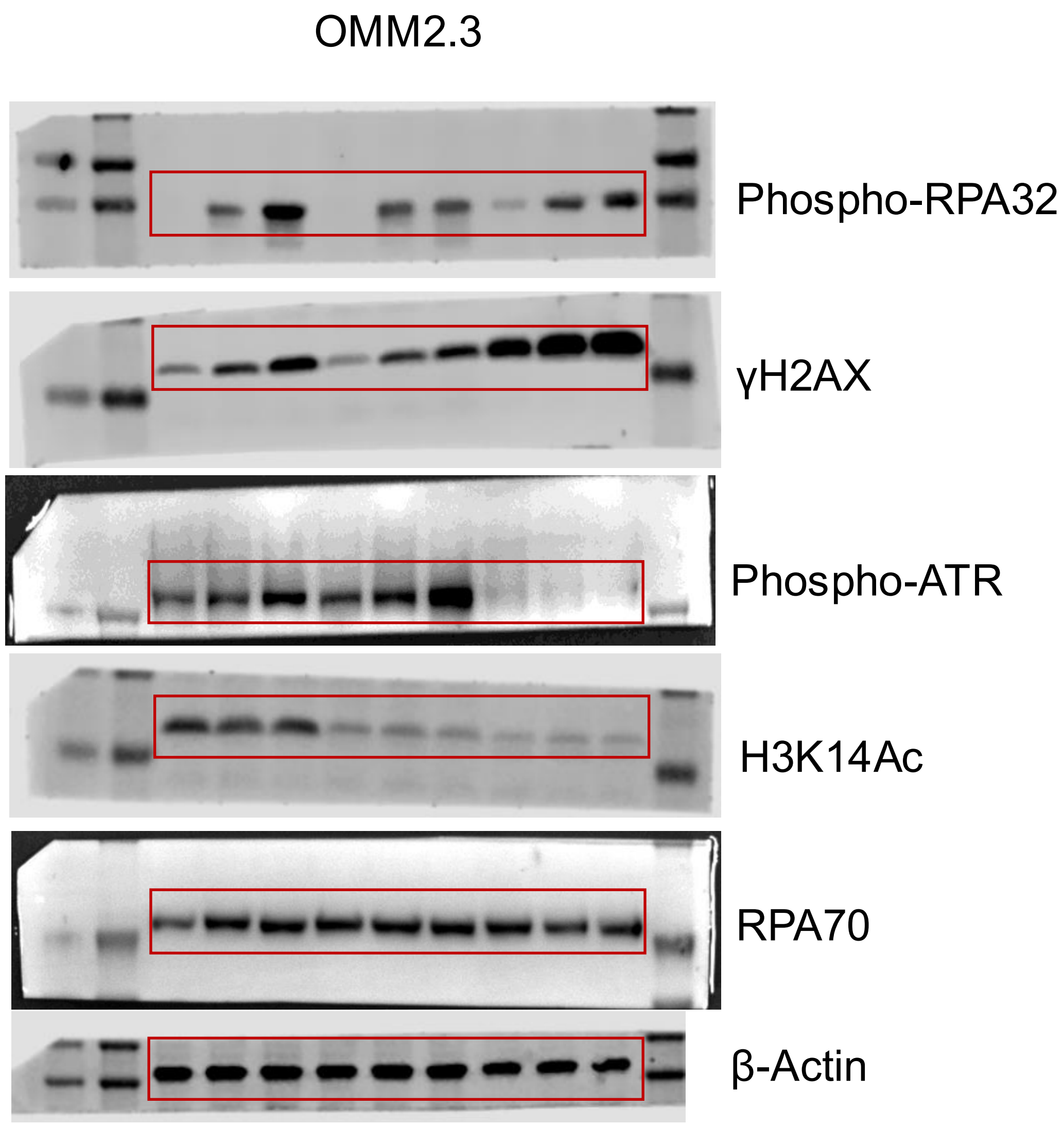
OMM2.3
Phospho-RPA32
γH2AX
Phospho-ATR
H3K14Ac
RPA70
β-Actin

Fig.S4A

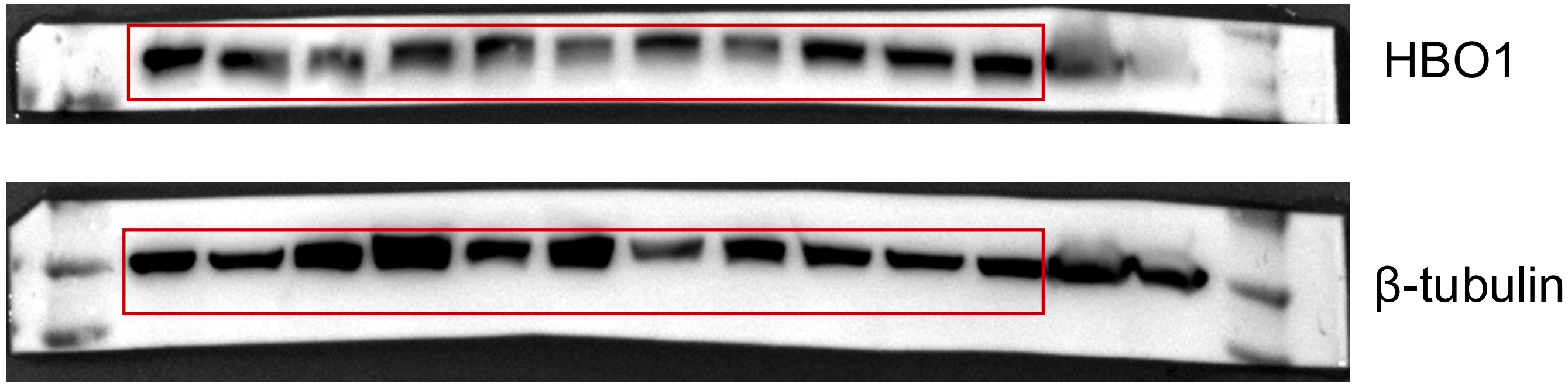

Fig.S4C

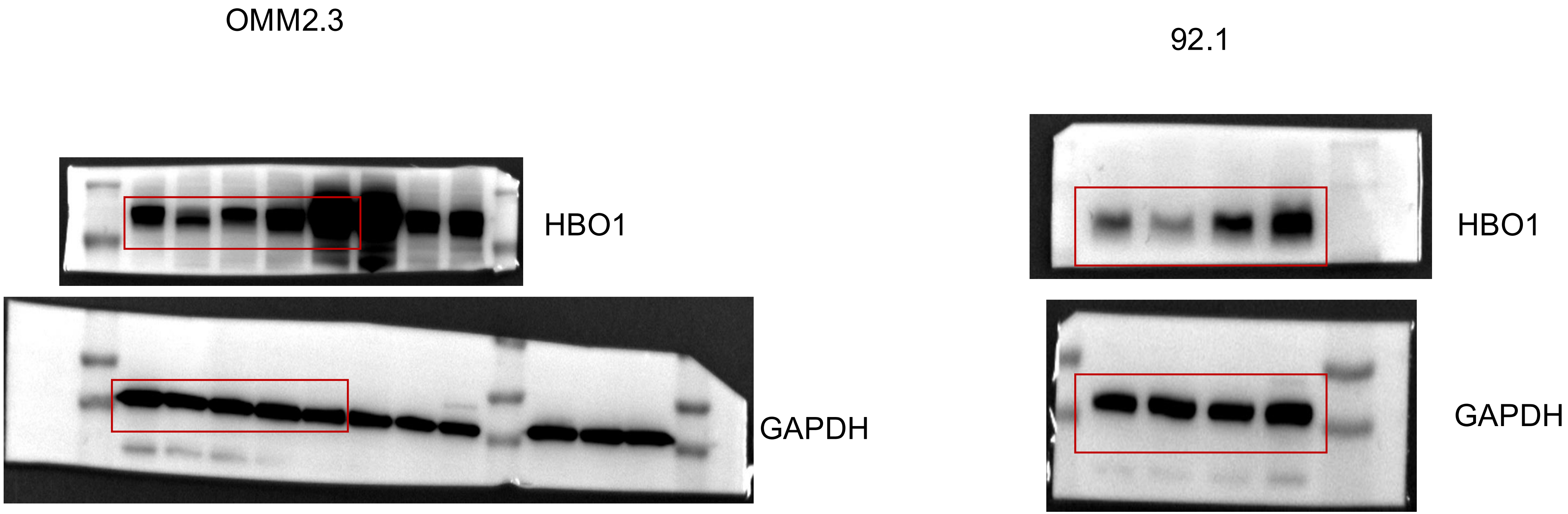

Fig.S6B

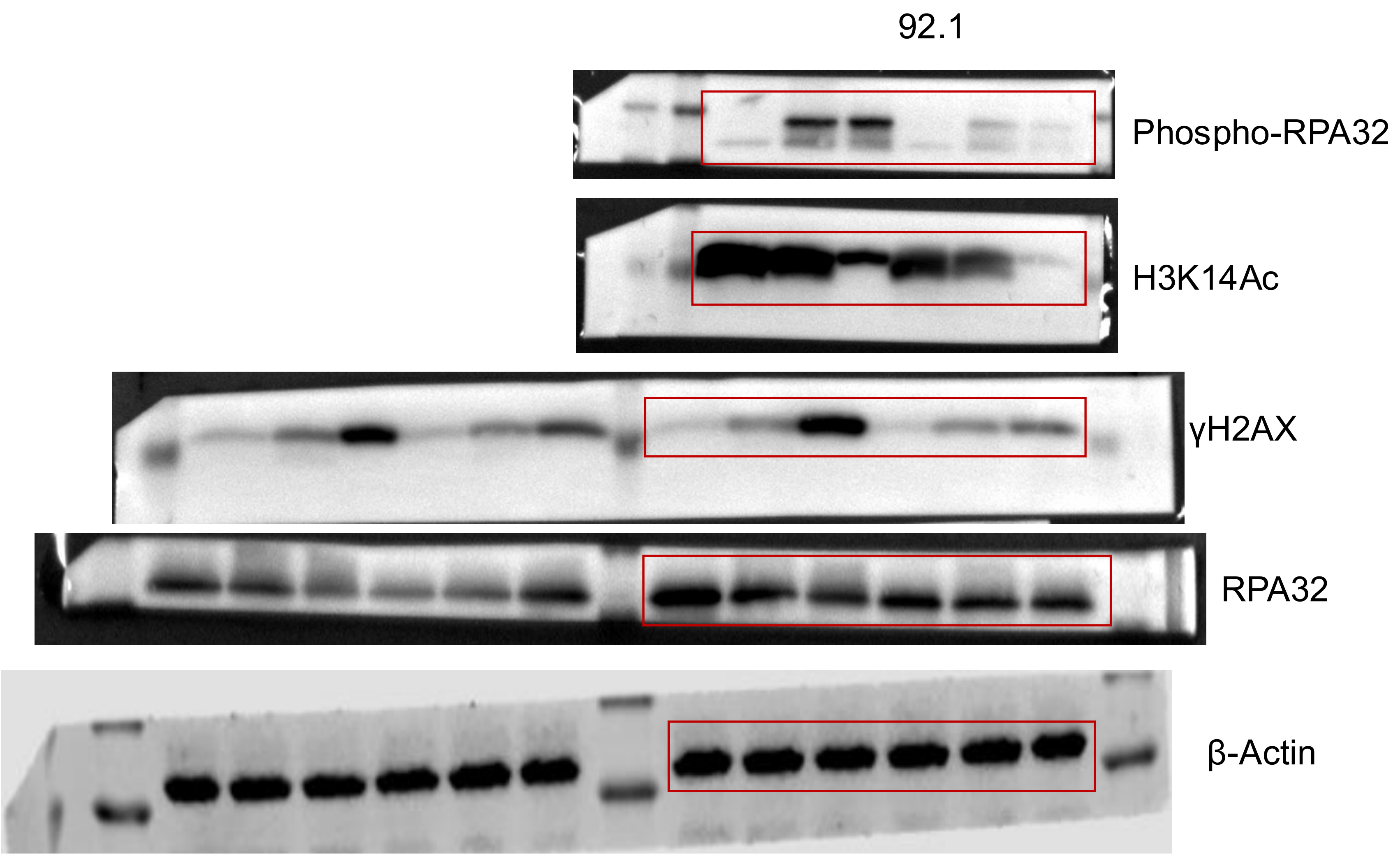

CT value Fig.6B

92.1		ATR	
shCtrl	20.28	19.76	20.22
shATR_1	20.94	20.97	20.64
shATR_2	20.97	21.44	20.99

92.1		β-Actin	
shCtrl	11.89	11.81	11.55
shATR_1	12.51	12.26	12.39
shATR_2	12.68	12.53	12.76

CT value Fig.S3E

OMM2.3

Sample	primer	CT value	primer	CT value
DMSO	actin	14.33	CNTRL	24.42
DMSO	actin	14.46	CNTRL	24.51
DMSO	actin	14.54	CNTRL	23.96
WT+WM-3835	actin	14.37	CNTRL	24.47
WT+WM-3835	actin	13.93	CNTRL	24.63
WT+WM-3835	actin	14.38	CNTRL	24.4
F_HBO1	actin	14.23	CNTRL	23.63
F_HBO1	actin	14.29	CNTRL	23.76
F_HBO1	actin	13.92	CNTRL	23.68
F_HBO1 SA	actin	14.44	CNTRL	24.56
F_HBO1 SA	actin	14.46	CNTRL	24.45
F_HBO1 SA	actin	14.48	CNTRL	-

Sample	primer	CT value	primer	CT value	primer
DMSO	actin	16.53	RIF1	22.44	CLOCK
DMSO	actin	16.58	RIF1	22.32	CLOCK
DMSO	actin	16.29	RIF1	22.46	CLOCK
WT+WM-3835	actin	16.54	RIF1	22.8	CLOCK
WT+WM-3835	actin	16.56	RIF1	23.33	CLOCK
WT+WM-3835	actin	16.26	RIF1	23.16	CLOCK
F_HBO1	actin	15.99	RIF1	21.87	CLOCK
F_HBO1	actin	16.41	RIF1	21.98	CLOCK
F_HBO1	actin	16.34	RIF1	22.52	CLOCK
F_HBO1 SA	actin	16.54	RIF1	22.51	CLOCK
F_HBO1 SA	actin	16.54	RIF1	22.53	CLOCK
F_HBO1 SA	actin	16.42	RIF1	22.49	CLOCK

CT value	primer	CT value	primer	CT value
22.91	TLK1	22	POLQ	24.93
22.81	TLK1	22.46	POLQ	24.51
22.83	TLK1	22.94	POLQ	24.63
23.43	TLK1	22.98	POLQ	26.42
23.34	TLK1	22.97	POLQ	25.74
23.68	TLK1	22.97	POLQ	26.28
22.6	TLK1	22.34	POLQ	24.63
22.95	TLK1	22.58	POLQ	25.36
22.56	TLK1	22.49	POLQ	24.85
23.42	TLK1	23.49	POLQ	25.56
23.39	TLK1	22.88	POLQ	25.58
23.43	TLK1	22.81	POLQ	25.31

CT value Fig.S4B

OMM2.3		HBO1	
Control	19.46	19.61	19.68
shHBO1_1	21.93	21.61	21.67
shHBO1_2	21.59	21.58	21.6
HBO1 OE	12.37	12.69	12.74

OMM2.3		β-Actin	
Control	11.88	12.62	12.48
shHBO1_1	12.89	12.87	12.89
shHBO1_2	11.54	11.9	11.94
HBO1 OE	12.71	12.55	12.35

Fig.2J

		HBO1 pS50/53 foci number			
	cell number	F_HBO1	F_HBO1 UV	F_HBO1 SA	F_HBO1 SA UV
Mean		9.075	15.64	1.318	6.809
95% CI Lower		7.904	14.34	0.7488	5.697
95% CI Upper		10.25	16.94	1.888	7.92
	1	13	20	10	3
	2	2	50	18	11
	3	17	26	16	4
	4	10	28	10	7
	5	8	19	7	15
	6	12	18	8	15
	7	10	14	12	13
	8	7	18	4	11
	9	6	5	24	7
	10	9	19	14	11
	11	10	21	8	8
	12	14	24	5	6
	13	22	25	6	10
	14	11	49	4	15
	15	10	44	5	17
	16	12	17	2	10
	17	12	16	4	31
	18	18	26	1	20
	19	17	24	0	23
	20	5	12	0	4
	21	0	18	0	4
	22	0	15	0	7
	23	0	10	0	2
	24	0	11	0	0
	25	0	6	0	0
	26	0	28	0	0
	27	0	13	0	0
	28	0	13	0	0
	29	0	9	0	0
	30	0	6	0	0
	31	0	11	0	0
	32	0	21	0	0
	33	0	18	0	0
	34	0	18	0	0
	35	0	20	0	0
	36	15	7	0	11
	37	20	11	0	2
	38	6	23	0	4
	39	16	20	0	3
	40	6	23	0	3
	41	11	27	0	10
	42	16	18	0	12

43	16	38	0	5
44	5	8	0	19
45	15	34	0	17
46	14	16	0	5
47	1	10	0	4
48	13	16	0	10
49	6	15	0	7
50	8	22	0	12
51	11	19	0	8
52	10	8	0	9
53	13	22	0	12
54	15	23	0	6
55	5	22	0	4
56	13	15	0	10
57	24	24	0	20
58	16	0	0	6
59	15	0	0	17
60	13	5	0	0
61	10	23	0	0
62	7	11	0	0
63	3	18	0	0
64	5	19	0	0
65	4	7	0	0
66	5	21	0	0
67	8	19	0	0
68	8	14	0	0
69	23	14	0	5
70	18	19	0	14
71	10	26	0	9
72	16	21	0	15
73	13	10	0	10
74	7	11	0	4
75	1	16	0	13
76	1	16	0	13
77	0	12	0	10
78	0	14	1	16
79	0	12	2	16
80	0	18	1	4
81	0	16	3	0
82	0	13	3	0
83	0	19	3	0
84	21	13	8	0
85	18	7	4	0
86	12	16	4	0
87	11	16	8	0
88	3	9	3	12
89	18	10	1	8
90	17	8	3	10

91	12	11	1	13
92	28	9	0	11
93	7	14	0	8
94	14	7	0	12
95	12	7	0	9
96	7	9	0	6
97	19	11	0	11
98	14	16	0	8
99	11	3	0	6
100	9	13	0	5
101	13	8	0	8
102	14	19	0	10
103	8	28	0	0
104	9	21	0	0
105	14	11	0	0
106	14	25	0	6
107	17	15	0	15
108	10	11	0	7
109	7	10	0	9
110	20	19	0	5
111	19	15	0	9
112	17	19	0	9
113	25	31	0	9
114	13	25	0	16
115	14	14	0	31
116	17	13	0	21
117	24	16	0	19
118	8	23	0	5
119	7	15	0	4
120	8	15	0	4
121	8	5	0	4
122	16	6	0	7
123	8	11	0	8
124	15	13	0	7
125	20	18	0	7
126	9	13	0	20
127	16	15	0	2
128	16	16	0	0
129	22	17	0	0
130	14	20	0	0
131	7	19	0	0
132	5	13	0	0
133	0	18	0	0
134	0	9	0	0
135	0	17	0	0
136	0	16	0	0
137	0	15	0	0
138	0	18	0	0

139	0	10	0	0
140	0	19	0	0
141	0	12	0	0
142	0	17	0	
143	0	25	0	
144	0	16	0	
145	0	11	0	
146	0	11	0	
147	0	11	0	
148		12	0	
149		9	0	
150		10	0	
151		9	0	
152		6	0	
153		0	0	
154		0	0	
155		0		
156		0		
157		0		

Fig.2L

	cell number	HBO1 pS50/53 foci number			
		DMSO	BAY1895344	WM3835 24h	WM3835 48h
Mean		11.18	6.828	4.542	1.644
95% CI Lower		10.2	5.593	3.857	1.095
95% CI Upper		12.16	8.062	5.226	2.193
	1	4	3	1	5
	2	15	11	3	5
	3	21	9	11	6
	4	14	6	3	5
	5	21	4	1	7
	6	20	9	8	10
	7	14	28	8	11
	8	14	21	10	4
	9	1	11	2	8
	10	23	9	9	7
	11	7	5	18	18
	12	4	6	8	1
	13	7	18	7	4
	14	3	14	2	5
	15	3	11	13	5
	16	17	17	12	9
	17	11	2	5	3
	18	5	6	1	4
	19	7	5	8	0
	20	11	11	12	0
	21	14	12	12	0
	22	6	25	14	0
	23	8	4	8	0
	24	11	8	7	0
	25	22	9	7	0
	26	20	0	13	0
	27	9	0	9	0
	28	9	0	5	0
	29	13	0	2	0
	30	10	0	6	0
	31	13	0	8	0
	32	5	0	11	0
	33	14	0	4	0
	34	21	0	11	0
	35	23	0	6	0
	36	23	0	7	0
	37	26	0	5	0
	38	12	0	11	0
	39	13	0	10	0
	40	25	0	10	0
	41	18	0	7	0
	42	5	0	5	0

43	7	0	17	0
44	7	0	12	0
45	27	0	12	0
46	26	0	13	0
47	15	0	8	0
48	17	0	12	0
49	17	14	14	0
50	21	14	8	0
51	16	16	9	0
52	17	14	10	0
53	17	10	9	0
54	5	13	5	0
55	13	13	3	0
56	22	12	7	0
57	17	5	10	0
58	1	7	5	0
59	3	8	15	0
60	8	2	5	0
61	31	5	7	0
62	9	4	7	0
63	7	23	4	0
64	17	15	11	0
65	10	25	11	0
66	20	2	16	0
67	36	19	8	0
68	24	9	11	0
69	15	13	5	0
70	8	7	4	0
71	9	22	8	3
72	6	21	9	3
73	6	11	9	2
74	7	3	7	4
75	16	2	0	4
76	13	17	0	5
77	9	14	0	6
78	3	15	0	7
79	3	14	0	7
80	7	17	0	8
81	7	14	0	1
82	5	7	0	12
83	9	4	0	13
84	4	14	0	2
85	1	4	0	3
86	8	23	0	2
87	10	1	0	6
88	3	13	0	3
89	0	13	0	2
90	0	17	0	4

91	0	12	0	3
92	30	8	0	0
93	9	12	0	0
94	2	2	0	0
95	22	3	0	0
96	11	9	0	0
97	15	18	0	0
98	6	12	0	0
99	11	11	0	0
100	9	15	0	0
101	18	16	0	0
102	5	16	0	0
103	1	26	0	0
104	6	11	0	0
105	7	11	0	0
106	10	3	0	0
107	11	14	0	0
108	14	24	0	0
109	17	2	0	0
110	3	2	0	0
111	21	3	0	0
112	12	0	0	0
113	14	0	0	0
114	12	0	0	0
115	22	0	0	0
116	16	0	4	0
117	14	0	6	0
118	17	0	12	0
119	15	0	4	0
120	6	0	8	0
121	19	0	12	0
122	1	0	7	0
123	21	0	3	0
124	8	0	3	0
125	19	0	4	0
126	16	0	7	0
127	19	0	5	0
128	11	0	4	0
129	24	0	8	0
130	12	0	3	0
131	7	0	7	0
132	18	0	14	0
133	2	0	24	
134	13	0	14	
135	4	0	7	
136	12	0	17	
137	10	0	10	
138	12	0	9	

139	7	0	16
140	6	0	6
141	20	0	9
142	20	0	11
143	13	0	5
144	3	0	10
145	1	0	4
146	18		6
147	25		3
148	8		7
149	7		11
150	14		8
151	19		5
152	12		15
153	20		7
154	10		13
155	13		7
156	14		7
157	10		6
158	13		13
159	17		2
160	15		2
161	9		5
162	22		0
163	3		0
164	10		0
165	13		0
166	15		0
167	12		0
168	16		0
169	12		0
170	11		0
171	7		0
172	7		0
173	20		0
174	8		0
175	12		0
176	14		0
177	9		0
178	10		0
179	15		0
180	12		0
181	12		0
182	12		0
183	10		0
184	8		0
185	17		0
186	11		0

187	14	0
188	9	0
189	4	0
190	1	0
191	12	0
192	12	0
193	23	0
194	14	0
195	8	0
196	3	0
197	16	0
198	11	0
199	0	0
200	0	0
201	0	0
202	0	0
203	0	0
204	0	0
205	0	0
206	0	0
207	0	0
208	0	0
209	0	0
210	0	0
211	0	0
212	0	0
213	0	0
214	0	0
215	0	0
216		0

Fig.4I

	cell number	phospho-RPA32 foci number WM-3835(0μM)	WM-3835(10μM)	WM-3835(20μM)
Mean		3.585	1.426	0.5172
95% CI Lower		2.824	1.043	0.2599
95% CI Upper		4.345	1.808	0.7746
	1	3	8	19
	2	1	4	10
	3	2	2	1
	4	4	8	2
	5	8	2	1
	6	4	1	1
	7	1	1	3
	8	20	5	4
	9	1	1	1
	10	9	1	1
	11	8	2	1
	12	7	6	1
	13	2	7	1
	14	4	8	0
	15	1	3	0
	16	16	5	0
	17	2	3	0
	18	3	1	0
	19	9	1	0
	20	20	2	0
	21	1	1	0
	22	1	1	0
	23	3	2	0
	24	5	2	0
	25	1	1	0
	26	12	1	0
	27	8	4	0
	28	1	2	0
	29	1	5	0
	30	1	0	0
	31	1	0	0
	32	7	0	0
	33	1	0	0
	34	0	0	0
	35	0	0	0
	36	0	0	0
	37	0	0	0
	38	0	0	0
	39	0	0	0
	40	0	0	0
	41	0	0	0
	42	0	0	0

43	0	0	0
44	0	0	0
45	0	0	0
46	0	0	0
47	0	0	0
48	0	0	1
49	0	0	1
50	0	0	1
51	0	0	1
52	0	0	1
53	0	0	2
54	0	0	0
55	0	0	0
56	4	0	0
57	1	0	0
58	3	0	0
59	2	0	0
60	3	0	0
61	2	0	0
62	4	0	0
63	7	0	0
64	4	0	0
65	1	0	0
66	1	0	0
67	2	0	0
68	5	1	0
69	3	1	0
70	3	1	0
71	5	1	0
72	2	1	0
73	6	3	0
74	1	6	0
75	9	1	0
76	10	6	0
77	10	2	0
78	12	4	0
79	10	6	0
80	1	1	0
81	13	1	0
82	6	12	0
83	14	23	0
84	1	1	0
85	3	5	0
86	1	2	0
87	3	1	0
88	2	4	0
89	5	1	0
90	10	12	0

91	2	7	1
92	1	1	1
93	5	4	1
94	1	3	1
95	0	5	1
96	0	0	1
97	0	0	2
98	0	0	2
99	0	0	8
100	0	0	5
101	0	0	0
102	0	0	0
103	0	0	0
104	0	0	0
105	0	0	0
106	20	0	0
107	1	0	0
108	3	0	0
109	1	0	0
110	1	0	0
111	1	0	0
112	1	0	0
113	1	0	0
114	1	0	0
115	2	0	0
116	2	0	0
117	5	0	0
118	2	0	0
119	5	0	0
120	11	0	0
121	10	0	0
122	7	4	0
123	4	2	0
124	4	3	0
125	2	3	0
126	5	2	0
127	3	3	0
128	1	3	0
129	5	3	0
130	10	5	0
131	1	2	0
132	13	3	0
133	6	4	0
134	7	5	2
135	8	1	4
136	20	1	1
137	0	3	1
138	0	5	2

139	0	4	3
140	0	7	1
141	0	1	1
142	0	1	1
143		3	1
144		1	10
145		3	2
146		0	0
147		0	0
148		0	0
149		0	0
150		0	0
151		0	0
152		0	0
153		0	0
154		0	0
155		0	0
156		0	0
157		0	0
158		0	0
159		0	0
160		0	0
161		0	0
162		0	0
163		0	0
164		0	0
165		0	0
166		0	0
167		0	0
168		0	0
169		0	0
170		0	0
171		0	0
172		0	0
173		0	0
174		0	0
175		0	0
176		0	0
177		0	0
178		0	0
179		0	0
180		0	0
181		0	0
182		0	0
183		0	0
184		0	0
185		0	0
186		0	0

187	0	0
188	0	0
189	0	0
190	0	0
191	0	0
192	0	0
193	0	0
194	0	0
195	0	0
196		0
197		0
198		0
199		0
200		0
201		0
202		0
203		0

Fig.5F

	cell number	phospho-RPA32 foci number F_HBO1	F_HBO1 SA
Mean		7.776	2.906
95% CI Lower		6.315	2.202
95% CI Upper		9.236	3.61
	1	1	1
	2	1	2
	3	18	1
	4	1	15
	5	25	12
	6	20	2
	7	3	20
	8	1	2
	9	16	4
	10	18	2
	11	5	13
	12	13	9
	13	5	6
	14	2	2
	15	27	5
	16	6	8
	17	23	1
	18	4	2
	19	4	2
	20	14	2
	21	17	10
	22	4	5
	23	5	1
	24	7	1
	25	1	14
	26	13	2
	27	10	3
	28	7	1
	29	7	0
	30	22	0
	31	4	0
	32	19	0
	33	22	0
	34	16	0
	35	5	0
	36	17	0
	37	16	0
	38	21	0
	39	7	0
	40	13	0
	41	21	0
	42	14	0

43	7	0
44	4	0
45	7	0
46	1	0
47	5	0
48	3	0
49	5	0
50	3	0
51	1	0
52	1	0
53	7	0
54	5	0
55	25	0
56	7	0
57	8	0
58	3	0
59	2	0
60	7	0
61	2	0
62	1	0
63	0	0
64	0	0
65	0	0
66	0	1
67	0	1
68	0	1
69	0	1
70	0	2
71	0	1
72	0	21
73	0	9
74	0	32
75	0	1
76	0	2
77	0	3
78	0	14
79	0	3
80	0	1
81	0	11
82	0	20
83	0	4
84	0	4
85	0	8
86	0	1
87	0	2
88	0	5
89	0	3
90	0	2

91	0	11
92	0	5
93	0	2
94	35	2
95	30	1
96	30	1
97	10	7
98	4	7
99	22	3
100	1	4
101	19	8
102	2	7
103	14	0
104	1	0
105	5	0
106	6	0
107	1	0
108	8	0
109	8	0
110	9	0
111	5	0
112	11	0
113	12	0
114	34	0
115	10	0
116	20	0
117	1	0
118	26	0
119	25	0
120	20	0
121	2	0
122	3	0
123	20	1
124	1	2
125	11	3
126	33	2
127	10	1
128	1	13
129	33	4
130	6	1
131	18	3
132	16	1
133	30	10
134	4	15
135	5	16
136	1	1
137	31	1
138	1	3

139	12	27
140	5	1
141	3	1
142	4	1
143	4	1
144	3	3
145	2	3
146	1	11
147	5	14
148	0	3
149	0	8
150	0	7
151	0	8
152	0	5
153	0	2
154	0	1
155	0	2
156	0	9
157		2
158		1
159		1
160		1
161		14
162		9
163		1
164		9
165		1
166		2
167		6
168		0
169		0
170		0
171		0
172		0
173		0
174		0
175		0
176		0
177		0
178		0
179		0
180		0
181		0
182		0
183		0
184		0
185		0
186		0

187	0
188	0
189	0
190	0
191	0
192	0
193	0
194	0
195	0
196	0
197	0
198	0
199	0
200	0
201	0
202	0
203	0

	cell number	γH2AX foci number F_HBO1	F_HBO1 SA
Mean		6.118	2.696
95% CI Lower		4.569	1.714
95% CI Upper		7.667	3.679
	1	4	29
	2	33	1
	3	5	1
	4	7	2
	5	16	1
	6	55	1
	7	2	8
	8	6	26
	9	8	2
	10	11	1
	11	27	3
	12	8	2
	13	20	0
	14	10	0
	15	9	0
	16	16	0
	17	9	0
	18	3	0
	19	12	0
	20	20	0
	21	22	0
	22	1	0
	23	6	0
	24	5	0
	25	1	0
	26	9	0
	27	12	0
	28	11	0
	29	16	0
	30	14	0
	31	7	0
	32	11	0
	33	1	0
	34	2	0
	35	32	0
	36	10	0
	37	2	0
	38	21	0
	39	7	0
	40	3	0
	41	2	0
	42	55	0

43	24	0
44	27	0
45	35	0
46	0	0
47	0	0
48	0	0
49	0	0
50	0	0
51	0	0
52	0	0
53	0	2
54	0	2
55	0	2
56	0	3
57	0	8
58	0	43
59	0	43
60	0	3
61	0	4
62	0	16
63	0	4
64	0	1
65	0	11
66	0	2
67	0	3
68	0	0
69	0	0
70	0	0
71	0	0
72	0	0
73	0	0
74	0	0
75	0	0
76	0	0
77	0	0
78	0	0
79	0	0
80	0	0
81	0	0
82	0	0
83	0	0
84	0	0
85	0	0
86	0	0
87	0	0
88	0	0
89	0	0
90	0	0

91	0	0
92	0	0
93	0	0
94	30	0
95	2	5
96	1	3
97	3	27
98	6	9
99	16	6
100	17	16
101	30	8
102	18	3
103	30	6
104	4	0
105	2	0
106	1	0
107	1	0
108	1	0
109	1	0
110	18	0
111	2	0
112	4	0
113	4	0
114	21	0
115	25	0
116	1	0
117	2	0
118	5	0
119	1	0
120	4	0
121	2	4
122	3	18
123	1	9
124	1	3
125	0	1
126	0	2
127	0	19
128	0	4
129	0	3
130	0	12
131	0	5
132	0	1
133	0	12
134	0	10
135	0	0
136	0	0
137	0	0
138	0	0

139	0	0
140	0	0
141	0	0
142	0	0
143	0	0
144	0	0
145	0	0
146	0	0
147	28	0
148	4	0
149	4	0
150	3	0
151	8	0
152	14	0
153	7	2
154	1	2
155	2	36
156	6	4
157	22	4
158	8	4
159	2	3
160	2	2
161	1	20
162	1	2
163	1	17
164	2	3
165	44	2
166	40	4
167	44	0
168	12	0
169	4	0
170	4	0
171	0	0
172	0	0
173	0	0
174	0	0
175	0	0
176	0	0
177	0	0
178	0	0
179	0	0
180	0	0
181	0	0
182	0	0
183	0	0
184	0	0
185	0	0
186	0	0

187	0
188	0
189	0
190	0
191	0

Fig.6E

	Percentage of cells with			
	WT	WT+HU	WM-3835	WM3835+HU
	0.005348	0.802083	0.016129032	0.594936709
	0.005952	0.913978	0	0.462962963
	0	0.854369	0	0.316326531
Mean	0.003767	0.8568	0.005376	0.4581
95% CI Lower	-0.004371	0.7177	-0.01776	0.1119
95% CI Upper	0.0119	0.9959	0.02851	0.8043

	Percentage of cells witl			
	WT	WT+HU	WM-3835	WM3835+HU
	0	0.422535	0	0.320754717
	0	0.520548	0	0.206349206
	0	0.46729	0	0.240963855
Mean	0	0.4701	0	0.256
95% CI Lower	0	0.3482	0	0.1103
95% CI Upper	0	0.592	0	0.4018

pRPA32 foci number > 10

WM3835+BAY1895344	WM3835+BAY1895344+HU
0.155844156	0.807692308
0.2	0.727272727
0.258426966	0.700854701
0.2048	0.7453
0.07693	0.607
0.3326	0.8835

n γH2AX foci number > 10

WM3835+BAY1895344	WM3835+BAY1895344+HU
0.178571429	0.879310345
0.112903226	0.846153846
0.13253012	0.85
0.1413	0.8585
0.0576	0.8134
0.2251	0.9035

Fig.S1

	cell number	Flag nuclear mean intensity(AU)			
		F_HBO1	F_HBO1 UV	F_HBO1 SA	F_HBO1 SA UV
Mean		37.16	27.23	33.68	29.98
95% CI Lower		31.71	23.48	28.47	25.66
95% CI Upper		42.61	30.98	38.89	34.29
	1	53.785	15.686	13.272	62.475
	2	110.3	42.684	18.603	80.651
	3	0	63.023	4.825	49.359
	4	90.268	53.473	36.857	10.968
	5	53.785	10.19	27.889	18.356
	6	110.3	28.506	102.488	19.312
	7	0	19.305	104.345	36.848
	8	49.364	7.859	14.632	13.031
	9	53.317	16.798	30.475	21.878
	10	75.973	71.412	34.51	26.163
	11	133.991	80.842	100.015	28.516
	12	68.308	36.044	3.33	53.784
	13	0	35.812	38.211	49.775
	14	0	16.426	21.74	7.425
	15	52.057	32.949	22.525	34.312
	16	49	25.324	55.062	24.494
	17	57.715	32.041	88.688	88.49
	18	57.17	18.171	145.215	13.165
	19	54	25.127	1.684	18.169
	20	51.229	22.364	0.811	48.202
	21	92.256	38.847	20.304	21.275
	22	121.152	15.526	55.81	54.741
	23	23.004	44.971	10.531	21.737
	24	19.326	51.477	33.713	26.182
	25	23.004	58.971	3.551	44.597
	26	19.326	13.954	88.739	34.847
	27	36.64	13.137	1.623	51.285
	28	18.91	26.052	18.006	77.718
	29	16.919	27.267	8.086	4.892
	30	16.418	2.564	11.146	17.25
	31	24.458	9.17	10.493	6.17
	32	49.503	54.888	19.335	14.527
	33	24.834	15.139	7.331	11.241
	34	45.789	48.602	5.307	3.007
	35	148.122	15.806	7.616	7.249
	36	106.169	26.225	4.671	4.563
	37	55.43	3.6	24.434	1.644
	38	38.339	99.248	25.6	23.1
	39	34.943	16.89	21.005	91.015
	40	14.79	8.023	69.969	16.645
	41	20.957	10.546	23.826	28.596
	42	6.244	16.722	17.754	23.979

43	9.718	53.477	30.332	28.5
44	6.86	35.852	1.565	13.293
45	8.547	15.513	19.895	51.306
46	5.13	10.881	39.614	19.624
47	2.523	12.174	17.919	24.038
48	13.572	10.26	49.34	13.799
49	36.983	5.676	73.527	6.724
50	17.438	1.21	31.822	18.375
51	3.227	31.371	68.173	4.94
52	4.401	25.211	46.255	2.132
53	26.996	12.647	19.329	0.923
54	35.08	32.062	50.68	2.733
55	28.625	5.968	47.078	29.32
56	25.39	43.706	19.069	11.631
57	7.296	0.893	25.977	0.911
58	17.524	6.389	43.776	0.981
59	18.967	3.168	41.322	4.073
60	11.175	3.536	52.902	54.105
61	15.243	0.97	18.913	3.492
62	29.789	1.745	10.213	13.632
63	11.89	13.079	36.014	4.265
64	57.726	8.353	46.188	3.658
65	14.805	15.757	40.015	29.906
66	32.888	19.459	53.89	83.387
67	39.578	7.616	36.209	56.3
68	33.454	53.396	42.43	6.019
69	46.095	37.032	35.109	35.832
70	41.465	65.48	35.486	31.202
71	32.564	47.76	7.732	26.337
72	62.483	64.223	9.845	15.693
73	2.869	38.665	5.066	27.347
74	85.168	32.196	8.461	1.638
75	65.421	12.792	7.629	1.196
76	70.242	18.726	11.976	4.496
77	21.826	18.411	9.119	14.354
78	38.126	11.506	23.513	12.465
79	23.844	19.089	50.64	8.657
80	25.803	83.017	28.462	4.11
81	80.569	71.394	29.346	29.113
82	19.909	12.002	44.61	22.502
83	22.723	5.194	5.719	34.537
84	25.129	9.453	51	13.696
85	17.127	20.074	70.719	45.594
86	42.753	29.923	127.624	15.802
87	4.216	58.268	84.153	40.285
88	4.684	9.447	37.448	34.823
89	31.353	10.394	38.082	89.933
90	3.508	23.271	120.093	15.108

91	73.505	20.407	75.305	30.802
92	4.154	9.175	53.754	102.761
93	46.194	9.351	76.573	85.925
94	40.649	60.529	26.627	17.972
95	60.776	1.538	48.421	38.001
96	56.416	38.828	14.82	49.206
97	1.493	4.621	29.189	17.635
98	0.744	3.416	33.792	2.076
99	6.737	1.552	26.949	8.056
100	18.816	48.179	24.998	15.626
101	8.36	20.313	5.2	22.651
102	24.761	2.668	32.232	39.895
103	59.668	7.339	44.376	22.67
104	61.146	5.006	28.704	6.592
105	69.603	66.999	11.744	31.458
106	80.58	61.352	3.389	11.652
107	52.029	25.423	31.788	6.305
108	25.932	72.182	36.102	16.826
109	55.717	32.338	9.274	26.158
110	42.405	57.225	1.758	28.874
111	19.363	21.856	1.701	29.589
112	19.519	21.086	1.589	26.955
113	48.943	15.395	15.584	44.66
114	101.054	14.717	43.187	36.891
115	39.325	10.742	44.928	46.539
116	29.573	9.864	30.592	26.519
117	23.107	14.28	29.469	58.894
118	10.009	38.236		59.571
119	24.044	41.241		44.91
120	4.336	19.365		162.813
121	13.1	26.397		91.942
122	51.911	29.909		37.807
123		88.401		44.985
124		38.869		97.191
125		23.94		26.653
126		40.861		21.195
127		21.489		59.557
128		50.772		31.684
129		36.511		46.317
130				83.132
131				63.859
132				63.971
133				12.117
134				9.047
135				10.221
136				8.147
137				44.462
138				20.868

139	19.952
140	11.412
141	20.81
142	14.691
143	23.598

	cell number	DMSO	BAY-1895344	WM-3835 24h	WM-3835 48h
		HBO1 nuclear mean intensity(AU)			
Mean		39.29	46.34	41	37.02
95% CI Lower		36.34	42.02	36.99	34.58
95% CI Upper		42.24	50.67	45.02	39.45
	1	42.637	139.27	41.402	57.508
	2	36.771	89.061	36.123	32.012
	3	43.631	90.587	91.098	56.653
	4	35.7	71.549	23.918	62.974
	5	21.235	60.691	20.976	40.219
	6	21.914	83.258	37.3	18.351
	7	14.861	122.508	46.185	29.242
	8	9.29	75.191	29.297	18.943
	9	27.017	121.466	12.471	67.864
	10	29.655	59.494	12.065	77.756
	11	22.763	17.367	2.277	42.476
	12	24.321	36.037	2.349	28.324
	13	23.123	56.189	13.417	59.241
	14	45.622	30.975	36.456	31.699
	15	24.833	45.455	26.073	35.766
	16	44.414	45.774	37.099	33.642
	17	19.325	51.991	28.099	47.147
	18	79.124	18.633	23.867	33.772
	19	95.423	74.817	17.808	37.057
	20	30.989	58.036	32.532	28.656
	21	28.509	40.709	46.399	41.734
	22	42.182	40.009	31.651	49.211
	23	71.448	59.935	36.947	35.816
	24	43.706	59.413	44.134	46.645
	25	47.471	67.661	35.214	50.385
	26	42.329	52.111	25.962	43.439
	27	16.889	41.658	34.821	38.296
	28	44.961	67.26	32.763	42.131
	29	37.375	106.551	31.799	26.63
	30	67.341	50.042	21.012	18.337
	31	34.571	68.661	26.164	19.334
	32	36.844	90.992	20.136	21.311
	33	55.443	54.471	22.482	55.584
	34	27.668	40.769	40.285	17.323
	35	38.856	55.805	31.133	58.914
	36	31.749	35.44	18.091	63.802
	37	33.868	81.785	16.007	43.889
	38	34.018	106.322	10.882	32.855
	39	25.658	75.103	40.656	34.91
	40	36.933	56.207	42.006	35.955
	41	30.856	53.82	43.712	23.148
	42	44.132	74.817	35.901	37.058

43	34.792	22.388	36.674	33.279
44	55.442	57.851	28.099	31.566
45	23.328	29.362	9.534	61.957
46	29.969	100.571	41.924	33.555
47	47.536	51.557	65.624	33.907
48	16.122	23.968	52.563	36.565
49	15.055	26.3	48.666	21.217
50	56.836	24.864	39.946	29.496
51	54.003	38.862	56.279	15.158
52	84.345	81.463	30.275	15.088
53	76.422	34.237	27.366	23.222
54	48.812	11.347	37.082	23.573
55	48.136	36.389	83.22	39.063
56	51.11	13.225	30.938	32.57
57	35.648	72.02	41.779	54.091
58	11.127	99.073	64.391	36.049
59	26.346	71.837	50.922	96.229
60	22.855	56.556	32.986	53.357
61	41.221	30.993	73.903	38.404
62	50.098	29.026	66.604	43.563
63	22.876	55.965	34.357	44.001
64	44.931	61.044	60.876	35.47
65	42.716	41.602	36.755	38.27
66	37.865	50.467	37.534	24.527
67	39.886	33.595	32.937	16.574
68	38.737	42.461	35.377	42.508
69	54.627	35.921	20.791	43.213
70	30.235	86.784	26.73	34.96
71	43.679	55.343	41.024	37.001
72	40.371	42.377	35.001	33.295
73	45.014	47.288	47.684	45.973
74	44.182	20.692	27.471	84.852
75	56.249	22.408	26.887	46.676
76	47.206	33.361	35.276	56.323
77	13.233	65.723	25.892	29.101
78	21.03	61.208	29.661	29.452
79	13.649	47.572	44.748	24.793
80	9.279	58.578	35.73	31.487
81	12.334	46.962	19.579	51.425
82	9.621	47.459	30.564	46.108
83	7.452	46.619	22.525	32.973
84	6.609	79.969	23.807	32.733
85	10.14	33.926	78.39	36.899
86	12.124	26.041	23.164	33.977
87	19.888	51.223	11.637	25.988
88	40.565	11.041	2.581	22.2
89	51.044	27.398	7.64	33.293
90	19.445	31.432	39.797	16.913

91	44.158	29.526	18.593	15.528
92	16.702	16.798	14.151	57.424
93	20.848	39.647	27.313	22.907
94	41.674	40.582	30.043	9.223
95	38.563	42.752	42.336	16.763
96	51.933	21.862	42.257	14.342
97	19.066	59.936	54.004	11.276
98	23.132	49.775	28.793	53.145
99	63.137	34.763	32.68	28.229
100	78.378	55.179	18.748	44.93
101	43.377	38.374	10.509	50.935
102	76.339	12.354	21.86	51.146
103	47.521	31.177	15.245	33.512
104	54.788	41.945	10.784	27.812
105	91.319	26.054	23.537	25.016
106	67.561	29.354	39.291	42.086
107	75.927	27.886	44.878	14.791
108	47.205	36.732	19.628	28.484
109	23.791	25.863	25.395	40.309
110	40.182	23.491	22.823	36.782
111	27.06	16.903	31.748	29.311
112	61.782	34.608	21.942	27.392
113	56.932	32.427	23.117	21.747
114	34.914	23.633	27.714	19.856
115	65.898	29.328	26.336	30.112
116	64.397	12.461	16.62	39.979
117	85.947	19.386	10.908	28.351
118	72.8	29.464	13.55	32.805
119	68.442	18.156	67.841	21.523
120	46.732	28.098	63.805	22.619
121	49.046	13.711	91.515	20.751
122	69.332	18.147	91.931	37.181
123	31.731	27.177	83.122	34.646
124	79.13	41.28	72.353	21.681
125	97.647	29.414	68.373	39.456
126	50.511	23.767	113.353	39.498
127	37.445	21.321	152.475	46.69
128	35.661	26.557	76.591	50.517
129	43.754	27.589	77.179	74.778
130	34.807	37.637	42.026	86.482
131	19.669	26.85	75.006	39.541
132	26.153	16.2	56.575	50.868
133	33.515	21.355	62.581	49.624
134	51.084		92.817	31.047
135	41.357		95.075	41.041
136	36.382		108.328	44.76
137	48.108		97.752	50.005
138	37.537		67.227	32.311

139	43.348	74.325	36.298
140	42.465	73.393	23.146
141	40.808	66.034	33.432
142	37.528	73.852	34.996
143	42.748	33.82	62.175
144	29.382	61.97	36.02
145	33.671	65.993	42.725
146	33.525	46.013	33.527
147	27.309	64.467	32.436
148	9.404	57.199	30.246
149	22.153	26.378	23.335
150	27.841	63.789	15.826
151	48.337		
152	21.308		
153	27.201		
154	15.379		
155	18.009		
156	1.595		
157	1.667		
158	58.196		
159	32.117		
160	66.463		
161	63.873		
162	46.471		
163	41.05		
164	18.979		
165	31.729		
166	34.828		
167	40.352		
